# ATF4 coordinates amino acid and nucleotide synthesis with selective protein translation to ensure proper DNA replication timing in leukemia cells

**DOI:** 10.1038/s41467-026-74324-1

**Published:** 2026-06-20

**Authors:** Jacklyn M. Huhn, Liana Valin, Mary Basse, Judith Sokei, Nishanth Gabriel, Esteban Martinez, Joice S. Kanefsky, Stephanie Stransky, Adrienne Dorrance, Ramiro Garzon, Daniela Di Marcantonio, Tomasz Skorski, Aaron R. Goldman, Hsin-Yao Tang, Samuele Cortellazzi, Orsola di Martino, John Krais, Simone Sidoli, Francesca Ferraro, David L. Wiest, Stephen M. Sykes

**Affiliations:** 1 Fox Chase Cancer Center, Philadelphia, PA USA; 2 Temple University Lewis Katz School of Medicine, Philadelphia, PA USA; 3 Washington University School of Medicine, Saint Louis, MO USA; 4 Albert Einstein College of Medicine, New York, NY USA; 5 Huntsman Cancer Institute at the University of Utah, Salt Lake City, UT USA; 6 Wistar Institute, Philadelphia, PA USA; 7 University of Parma, Parma, Italy

**Keywords:** Checkpoints, Cancer metabolism, DNA replication, Translation

## Abstract

Proper timing of DNA replication relies on sufficient nucleotide pools and replication machinery. The upstream regulatory programs that support the biomass production needed for DNA replication, particularly in the accelerated growth setting of cancer, remain incompletely defined. Here we show that the transcription factor ATF4 coordinates amino acid and nucleotide metabolism with selective protein synthesis to ensure proper DNA replication initiation and timing in acute leukemia. Specifically, ATF4 promotes the expression of enzymes that biosynthesize amino acids required for nucleotide production and drive the transcription of tRNA charging enzymes that sustain translation of a subset of proteins involved in replication origin firing. Consequently, ATF4 inhibition limits nucleotide biosynthesis and replication machinery, thereby disrupting DNA replication timing and leading to leukemia cell differentiation and death. Our findings indicate that ATF4 coordinates metabolic and translational programs to maintain DNA replication fidelity and the differentiation blockade in leukemia cells.

## Abstract (web summary)

Cancer cells require accelerated biomass production to fuel DNA replication. Here, the authors show that ATF4 couples amino acid and nucleotide synthesis with translation of replication machinery to ensure proper DNA replication timing in leukemia.

## Introduction

The mammalian cell cycle is a tightly regulated, unidirectional process that requires the precise coordination of several processes, including the synthesis of select metabolites and proteins. In particular, as eukaryotic cells transition from the G1- to S-phase of the cell cycle, they undergo extensive metabolic and proteomic remodeling to accumulate the biomass needed for DNA replication and subsequent cellular duplication^1–3^. For instance, de novo nucleotide synthesis increases during late G1 to support RNA synthesis and DNA replication during S-phase^4,5^. Rates of protein synthesis also escalate in late G1, impart to translate mRNAs encoding proteins necessary for DNA replication^2,6,7^. Central to both nucleotide and protein synthesis, as well as many other anabolic processes, non-essential amino acid (NEAA) production and glutamine anaplerosis also ramp up during the G1/S transition^4,5,8^. While cell cycle-dependent changes in protein translation and cellular metabolism have been well studied, our understanding of the upstream factors, particularly transcriptional regulators, that coordinate the complex interplay of these biosynthetic processes remains incomplete.

The basic leucine zipper (bZIP)-containing transcription factor ATF4 has been implicated in the regulation of both metabolic adaptation and protein synthesis. The intracellular relationship between ATF4 and metabolism was initially observed under conditions of nutrient deprivation. Specifically, the withdrawal of glucose or certain amino acids leads to ATF4 accumulation, and mouse embryonic fibroblasts (MEFs) lacking *Atf4* are unable to survive in the absence of NEAA^9,10^. ATF4 also drives the transcription of genes that promote the synthesis, import, and tRNA aminoacylation (also referred to as tRNA charging) of several amino acids in response to a variety of stimuli, including amino acid withdrawal, endoplasmic reticulum (ER) stressors, tricarboxylic acid (TCA) cycle perturbations, and insulin treatment^10–15^. By promoting amino acid import and synthesis, as well as tRNA charging, ATF4 contributes to redox homeostasis, de novo purine synthesis, and mTORC1-driven protein translation^10,14–18^.

As a mediator of metabolic programming, ATF4 is often co-opted in cancer. For example, experimental models of colorectal cancer (CRC), fibrosarcoma, and KRAS-driven non-small cell lung carcinoma (NSCLC) rely on ATF4 to support their asparagine demands in vivo^19,20^. Many NSCLC cell lines also depend on ATF4 to support glutathione and nucleotide production through transactivation of the serine synthesis pathway (SSP) and *SHMT2*^21^. ATF4 also plays a pro-tumorigenic role in aggressive subtypes of acute myeloid leukemia (AML), in part through transcriptional activation of the SSP enzymes. Specifically, the expression and activity of ATF4 is elevated in leukemia-initiating cells^22,23^, and inhibition of ATF4 significantly impedes disease progression in mouse models of AML driven by KMT2A-rearrangements^24^. Moreover, ATF4 inhibition leads to decreased expression of both basal and stress-activated expression of the SSP enzymes^25,26^. However, the precise molecular role of ATF4 in AML remains largely undefined.

We report that ATF4 transcriptionally regulates basal amino acid biosynthesis and tRNA charging to coordinate de novo nucleotide synthesis with the preferential translation of proteins needed for the initiation and proper timing of DNA replication in leukemia cells. Using both DNA fiber assays and Repli-seq analyses, we show that the disruptions in ATF4-driven intracellular metabolism and selective protein synthesis decrease leukemia cell DNA replication origin firing and speed. Our comprehensive study provides a detailed map of how canonical ATF4 transcriptional programs, encoding multiple biosynthetic processes, are precisely coordinated to facilitate the accurate timing and speed of DNA replication in leukemia cells.

## Results

### ATF4 supports AML cell cycling and survival

**Fig. 1:**
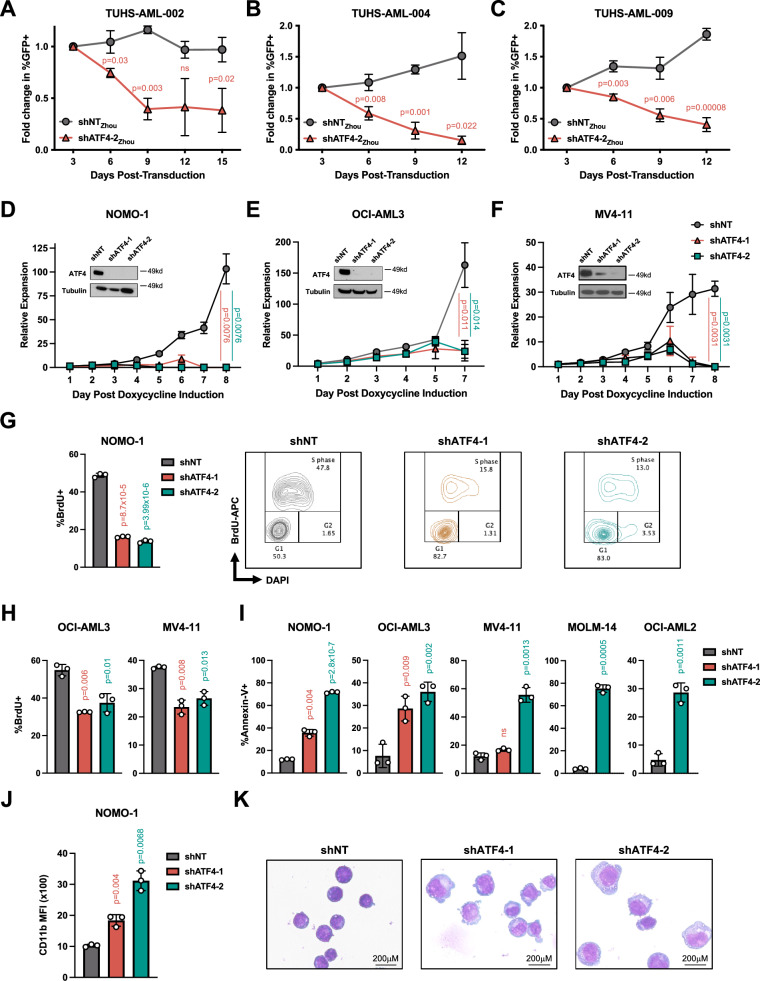
ATF4 supports AML cell cycling and survival. **A**–**C** Cryopreserved patient-derived samples were transduced with lentiviruses co-expressing GFP and shNT or shATF4 and then monitored for changes in %GFP by flow cytometry over the indicated time points post-transduction. Data represent the mean ± SD of three technical replicates from a single biological replicate performed per patient sample. Statistics are derived from a two-sided, unpaired *t*-test using Welch’s correction (95% CI) comparing the shNT control and shATF4-2_Zhou_ conditions at each time point. Multiple comparisons were carried out using a two-stage linear Benjamini–Krieger–Yekutieli (BKY) procedure (exact p- and q- values are listed in Source Data File). **D**–**F** NOMO-1 (**D**), OCI-AML3 (**E**), and MV4-11 (**F**) cells expressing DOX-inducible shNT, shATF4-1, or shATF4-2 were administered DOX and then analyzed for proliferation using an MTS assay at the indicated time points. Data are plotted as the fold change in MTS signal at each time point relative to that prior to DOX treatment. Inset, western blot analysis of ATF4 protein expression at 24 (NOMO-1 and MV4-11) or 48 h (OCI-AML3) post-DOX. For panels **D**–**F**, data represent the mean ± SD of three technical replicates. Each experiment was repeated in three independent, biological replicates. Statistics are derived from a two-sided, unpaired *t*-test using Welch’s correction (95% CI) comparing the shNT control with the shATF4-1 (pink *p*-values) or shATF4-2 (teal *p*-values) conditions. Multiple comparisons were carried out using a two-stage linear BKY procedure (exact p- and q- values are listed in Source Data File). **G** DOX-induced NOMO-1 cells expressing shNT, shATF4-1, or shATF4-2 were assessed for BrdU incorporation and DNA content by flow cytometry. *Left*, histogram analysis of flow data acquired using the contour plots presented to the *Right*. **H** Percentage of S-phase (i.e., BrdU+) of OCI-AML3 (*left*) and MV4-11 (*right*) for each shRNA condition. **I** NOMO-1 (*far left*), OCI-AML3 (*middle left*), MV4-11 (*middle*), MOLM-14 (*middle right*), and OCI-AML2 (*far right*) were analyzed for percent Annexin V positivity by flow cytometry. **J** DOX-induced NOMO-1 cells expressing shNT, shATF4-1, or shATF4-2 were analyzed by flow cytometry for CD11b expression (MFI, median fluorescence intensity) at 7 days post doxycycline induction. For panels **G**–**J**, data represent the mean ± SD of three technical replicates. Each experiment was repeated in three independent, biological replicates. Statistics are derived from a two-sided, unpaired *t*-test (95% CI) comparing the shNT control with the shATF4-1 (pink *p*-values) or shATF4-2 (teal *p*-values) conditions. Adjustments for multiple comparisons were not made. Exact *p*-values are listed in the Source Data File. **K** DOX-induced NOMO-1 expressing shNT, shATF4-1, or shATF4-2 were analyzed by light microscopy for morphological changes at 4 days post doxycycline induction (40× mag.; 200 µM bar). Images represent one slide from a replicate of three independent, biological replicates. Source data for (**A**–**J**) are provided in the Source Data file.

ATF4 is activated in primary human AML patient samples and is associated with leukemia-initiating cell potential^22,23^. Consistent with these observations, steady-state levels of *ATF4* are significantly higher in multiple AML subtypes, including KMT2A-rearranged and normal karyotype-AML (NK-AML), relative to healthy hematopoietic cells in two independent AML patient-derived (PD-AML) gene expression profiles (Supplementary Fig. 1A, B). We previously showed that ATF4 functionally supports leukemia cell survival and disease initiation in a mouse model of AML driven by the KMT2A-MLLT3 rearrangement^24^. To assess a potential functional role of ATF4 in patient-derived AML (Supplementary Data 1), we employed a previously validated shRNA-mediated approach^24^. Compared to non-targeting shRNA (shNT) controls, inhibition of ATF4 (shATF4) reduced the ability of patient-derived AML cells to persist in co-culture with HS-27 stromal cells (Fig. 1A–C and Supplementary Fig. 1C–F)^26–28^. Next, a doxycycline-inducible shRNA system targeting ATF4 was utilized to assess the time-dependent effects of ATF4 knockdown on leukemia cell growth dynamics. Doxycycline (DOX)-mediated induction of ATF4-targeting shRNAs substantially reduced ATF4 expression within 16–24 h in the human AML cell lines, NOMO-1, OCI-AML3, MV4-11, K-562, MOLM-14, and OCI-AML2 (Fig. 1D–F and Supplementary Fig. 1G–I). ATF4 inhibition significantly reduced the expansion of all six cell lines beginning at different time points post DOX-induction of shRNAs (Fig. 1D–F and Supplementary Fig. 1G–I).

To assess the impact of ATF4 inhibition on AML cell biology, DOX-treated cells from each shRNA condition were assessed for changes in cell cycling, survival, and maturation state. Compared to controls, NOMO-1, OCI-AML3, and MV4-11 cells expressing ATF4-targeting shRNAs displayed a significant reduction in BrdU incorporation with a concomitant accumulation in G1 at 72-h post-DOX, indicating that ATF4-inhibition causes these cells to arrest within the G1/S transition (Fig. 1G, H). At later time points post-DOX treatment, shATF4-expressing cells displayed a significant decline in cell survival, as measured by Annexin-V staining (Fig. 1I). Several AML cell lines expressing ATF4-targeting shRNAs also displayed increased expression of surface markers associated with myeloid maturation, such as CD11b and/or CD16, compared to controls (Fig. 1J and Supplementary Fig. 1J–L). Furthermore, ATF4 knockdown caused cells to acquire characteristics of myeloid differentiation, such as increased asymmetry and cytoplasmic volume (Supplementary Fig. 1K and Supplementary Fig. 1M–O). Collectively, these data suggest that ATF4 supports cell cycling, differentiation blockade, and survival of AML cells.

### ATF4 regulates transcriptional programs associated with amino acid biosynthesis, import, and tRNA charging

**Fig. 2:**
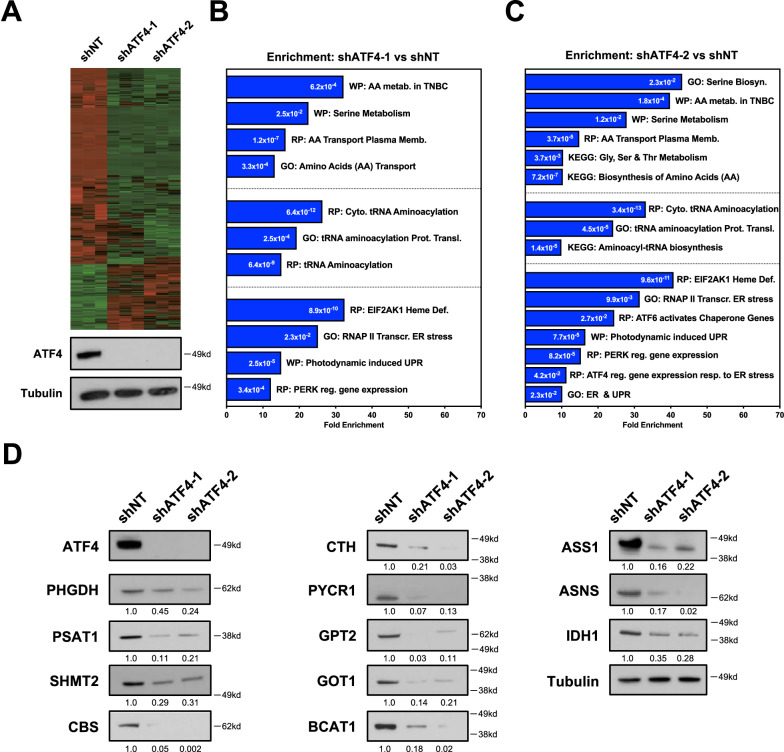
ATF4 regulates transcriptional programs associated with amino acid synthesis, import, and tRNA aminoacylation. **A** *Top*, Heatmap of differentially expressed genes between NT- and ATF4-shRNA-expressing NOMO-1 cells. *Bottom*, Western blot analysis of FACS-purified NOMO-1-RFP+ cells expressing DOX-inducible shNT, shATF4-1, or shATF4-2 was conducted for each replicate. RNA-seq data represent 3 replicates of the only biological replicate. **B**, **C** Pathway enrichment analysis comparing RNA-seq profiles of shNT vs shATF4-1 (**B**) or shNT vs shATF4-2 (**C**). The analysis was performed using GO_BP (GO), KEGG, Reactome (RP), and Wiki Pathways (WP). Benjamini scores are shown in white within each bar graph. **D** Following 48 h of DOX-treatment, NOMO-1 cells from each shRNA condition were subjected to western blot with the indicated antibodies. Quantified protein levels were normalized to tubulin. Images represent one replicate of three independent, biological replicates. Source data for all panels are provided in the Source Data file.

To identify the downstream molecular pathways through which ATF4 supports AML cell biology, RNA-sequencing (RNA-seq) was performed on NOMO-1 cells from each shRNA condition, 24 h post-DOX treatment (Fig. 2A). A comparison of the transcriptomes using a statistical cut-off of adjusted *p* < 0.05 and fold change (log2) > 1.0 and <−1.0 revealed that inhibition of ATF4 led to a significant decrease in the expression of 480 and 358 transcripts in shATF4-1 and shATF4-2 conditions, respectively, and an increase in the expression of 119 transcripts in both conditions when compared to the control (Supplementary Fig. 2A). To identify molecular pathways affected by ATF4 inhibition, a pathway enrichment analysis was carried out using the Database for Annotation, Visualization and Integrated Discovery (DAVID) focusing on the Gene Ontology Biological Processes (GO_BP), Kyoto Encyclopedia of Genes and Genomes (KEGG), Reactome, and Wiki Pathways^29^. An enrichment analysis of genes downregulated by ATF4 inhibition, identified 25 and 32 pathways significantly different (Benjamini–Hochberg, *q* < 0.05) between shNT and shATF4-1 or shATF4-2, respectively (Supplementary Data 2). Many of the highly enriched pathways (particularly those with an enrichment score > 10) segregated into three major categories: amino acid metabolism, tRNA aminoacylation/charging, and UPR signaling (Fig. 2B, C). Of note, these effects from ATF4 inhibition were independent of *KMT2A*-driven transcriptional programs, as only 1.19% of significantly downregulated genes overlapped with previously published *KMT2A* gene expression signatures (Supplementary Data 3)^30^. These findings were further supported by an expression analysis of shNT- versus shAtf4-expressing murine MLL-AF9 leukemia cells (Supplementary Fig. 2B), and confirmed through an independent gene set enrichment analysis utilizing the Molecular Signatures Database (MSigDb) (Supplementary Data 4).

An individual gene analysis showed that ATF4 inhibition resulted in decreased expression of key genes encoding enzymatic regulators of the SSP, one-carbon metabolism, cysteine synthesis, glutamate and aspartate metabolism, alanine synthesis, branched chain amino acid (BCAA) catabolism, arginine and proline synthesis, the TCA cycle, as well as multiple amino acid transporters (e.g., SLC1A4, SLC1A5, SLC6A9, SLC7A1, SLC7A2, SLC7A5, SLC7A11, SLC38A1, SLC38A2, SLC43A1). Quantitative-PCR (qPCR) validation confirmed that ATF4 inhibition resulted in decreased steady-state levels of many of these enzymes in DOX-treated NOMO-1, OCI-AML3, and MV4-11 cells (Supplementary Fig. 2C–E). AML cells expressing ATF4-targeting shRNAs also showed decreased protein levels of all three SSP enzymes, PHGDH, PSAT1, and PSPH (Fig. 2D and Supplementary Fig. 3A and 3B). We also observed a reduction in several other enzymes involved in de novo amino acid synthesis, including CBS, CTH, PYCR1, GPT2, BCAT1, ASNS, and ASS1 (Fig. 2D and Supplementary Fig. 3A and B). ATF4 inhibition also resulted in decreased expression of GOT1 and IDH1 in NOMO-1 and OCI-AML3 cells (Fig. 2D and Supplementary Fig. 3A). Consistent with other studies^15,16,21,31,32^, ATF4 knockdown resulted in decreased steady-state levels of *SHMT1*, *SHMT2*, *MTHFD2*, *MTHFD1L*, and *ALDH1L2* across multiple AML cell lines (Supplementary Fig. 2C–E). However, the impact of ATF4 inhibition on protein levels of these one-carbon metabolism enzymes varied across cell lines. For example, ATF4 inhibition did not impact SHMT1 or MTHFD1L expression across multiple cell lines (Supplementary Fig. 3B and C). On the other hand, ATF4 inhibition led to decreased (though to varying degrees) SHMT2 protein levels across all cell lines analyzed (Fig. 2D and Supplementary Fig. 3A, B). While ALDH1L2 protein expression was not detected in NOMO1 cells, ATF4 knockdown resulted in decreased ALDH1L2 expression in OCI-AML3, MOLM-14, and OCI-AML2 cells (Supplementary Fig. 3B). MTHFD2 is a well-established transcriptional target of ATF4^15,16,21,31,32^, and MTHFD2 levels decrease upon ATF4 knockdown in NOMO-1 and OCI-AML3 (Supplementary Fig. 2C, D). While MTHFD2 protein levels were decreased in MOLM-14 and OCI-AML3 cells upon ATF4 knockdown, MTHFD2 levels were minimally impacted by ATF4 inhibition in NOMO-1 and OCI-AML2 (Supplementary Fig. 3B).

To assess the functional relevance of these metabolic genes, we queried the dependency of each gene in AML, hematological malignancy (Heme), solid cancer (Solid), and non-cancerous (NC) cell lines using the Cancer Dependency Map (Depmap) database^33^. Consistent with our functional data, AML and Heme cell lines displayed average Chronos scores of −0.79 and −0.82, respectively, indicating that these cell lines are highly dependent on ATF4 (Supplementary Fig. 4). In addition to ATF4, AML cell lines were highly dependent on several amino acid transporters such as SLC38A2 (−0.57), SLC7A1 (−0.54), SLC7A5 (−0.42), as well as the amino acid biosynthesis enzymes PSAT1 (−0.26) and ASS1 (−0.24) (Supplementary Fig. 4). Although Solid and NC cell lines are also largely dependent on ATF4, PSAT1, and ASS1, AML and Heme cell lines appear to be selectively dependent on SLC38A2, SLC7A1, and SLC7A5 compared to solid and NC cell lines (Supplementary Fig. 4).

### Metabolites associated with amino acid and nucleotide synthesis are reduced by ATF4 inhibition

**Fig. 3:**
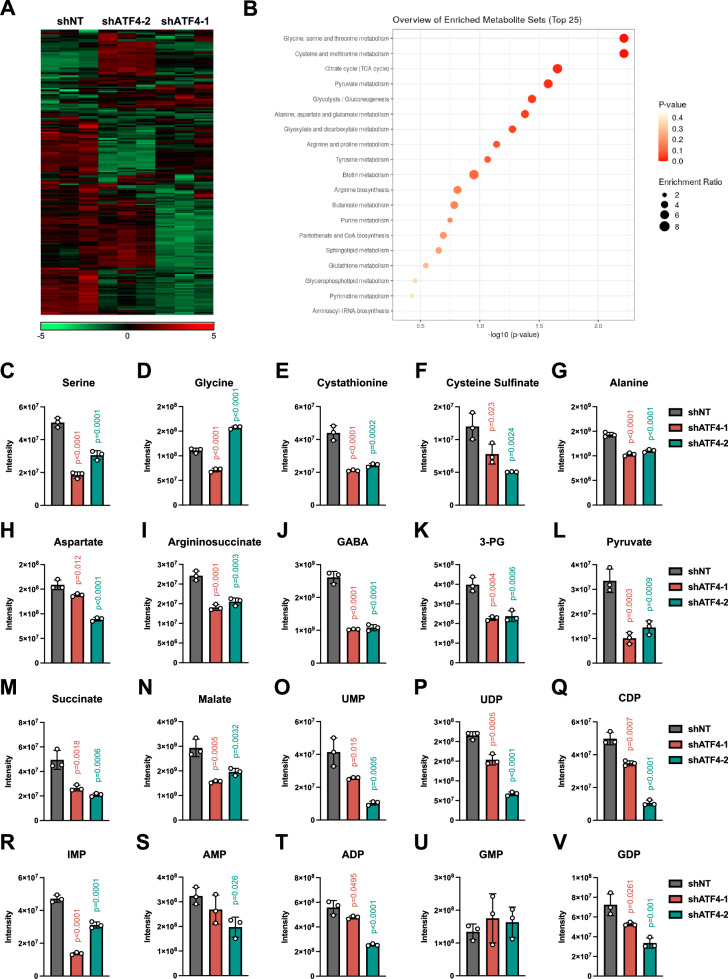
ATF4 inhibition reduces steady-state levels of amino acid and nucleotide intermediates. TPMs extracted from NOMO-1 cells expressing shNT, shATF4-1, or shATF4-2 and analyzed by HILIC-MS. **A** Heatmap of steady-state polar metabolite profiles from DOX-treated NOMO-1 cells expressing shNT, shATF4-1, or shATF4-2. **B** Pathway enrichment analysis of metabolites that were down-regulated by shATF4-2 using the MetaboAnalyst v5.0. **C**–**V** Quantification of the following steady-state TPM levels in NOMO-1 expressing shNT, shATF4-1, or shATF4-2: **C** serine, **D** glycine, **E** cystathionine, **F** cysteine sulfinate, **G** alanine, **H** aspartate, **I** argininosuccinate, **J** GABA, **K** 3-PG, **L** pyruvate, **M** succinate, **N** malate, **O** UMP, **P** UDP, **Q** CDP, **R** IMP, **S** AMP, **T** ADP, **U** GMP, and **V** GDP. For **C**–**V**, all graphed data represent the mean ± SD of three technical replicates from one biological replicate. Two independent, biological replicates were carried out. Statistics are derived from a one-way ANOVA comparing the shNT control and the shATF4-1 (pink *p*-values) and shATF4-2 (teal *p*-values) conditions. A Dunnett’s multiple comparisons test (95% CI) was also performed. Exact adjusted *p*-values are listed in the Source Data File. Source data for (**A**), as well as **C**–**V** are provided in the Source Data file.

To assess whether the reductions in metabolic enzyme expression mediated by ATF4 inhibition resulted in changes to the cellular metabolome, hydrophilic interaction liquid chromatography mass spectrometry (HILIC-MS) was carried out on NOMO-1 cells from each shRNA condition at 48 h post-DOX treatment. This time point was selected to ensure that total polar metabolite (TPMs) profiles reflected a reduction in ATF4 expression and not an alteration in cell biology (e.g., cell cycle arrest or cell death). Additionally, the expression of several ATF4-regulated metabolic enzymes were reduced by 40 h post-DOX (Supplementary Fig. 5A). The steady-state levels of numerous TPMs were significantly different between control and shATF4-expressing cells (Fig. 3A). A comparison of the TPM profiles using MetaboAnalyst^34^ between shNT- and shATF4-2-expressing NOMO-1 cells indicated that several interconnected metabolic pathways are affected by ATF4 inhibition including: (1) Glycine, serine, and threonine metabolism; (2) Cysteine and methionine metabolism; (3) Alanine, aspartate, and glutamate metabolism; and (4) Arginine and proline biosynthesis (Fig. 3B and Supplementary Fig. 5B, C). The TCA cycle and pyruvate metabolism, as well as the production of purines and pyrimidines, were also altered by ATF4 inhibition (Fig. 3B and Supplementary Fig. 5C).

Many of the changes in amino acid levels mediated by ATF4 inhibition aligned with the decreases in metabolic enzyme expression found in shATF4-expressing cells. For example, steady-state levels of serine were decreased in shATF4-expressing cells (Fig. 3C), which is consistent with reduced expression of the SSP enzymes (Fig. 2D and Supplementary Fig. 3A, B). However, levels of glycine, which can be synthesized from serine or imported^35^, varied among the two ATF4-targeting shRNAs (Fig. 3D). In the transsulfuration pathway, serine is utilized by cystathionine beta synthase (CBS) to generate cystathionine, which can be subsequently converted into cysteine by cystathionine gamma-lyase (CTH) (Supplementary Fig. 5B)^36^. NOMO-1 cells expressing ATF4-targeting shRNAs, not only displayed reduced expression of CBS and CTH (Fig. 2D), but also exhibited a decrease in cystathionine, as well as the downstream cysteine metabolite, cysteine sulfinate (Fig. 3E, F). Consistent with the decrease in glutamic--pyruvic transaminase 2 (GPT2) and glutamic-oxaloacetic transaminase 1 (GOT1) expression, shATF4-expressing NOMO-1 cells displayed significantly lower steady-state levels of alanine and aspartate relative to control cells (Fig. 3G, H). In accordance with reduced argininosuccinate synthase 1 (ASS1) expression, shATF4-expressing NOMO-1 cells also displayed a significant decrease in the urea cycle intermediate, argininosuccinate (Fig. 3I). However, steady-state levels of arginine and proline, both of which are potential downstream products of the urea cycle, were not significantly different between shNT- and shATF4-expressing NOMO-1 cells (Supplementary Fig. 5D, E). Despite reduced expression of the BCAA transaminase, BCAT1, levels of isoleucine, leucine, and valine were not significantly altered by ATF4 inhibition (Supplementary Fig. 5F–H). Steady-state levels of glutamate (Supplementary Fig. 5I) were largely unaffected by ATF4 inhibition, but compared to control cells, shATF4-expressing cells displayed significantly lower levels of the glutamate-derivative, GABA (γ-aminobutyric acid) (Fig. 3J). Lastly, ATF4 inhibition did not result in substantial alterations in steady-state levels of glutamine (Supplementary Fig. 5J).

Intermediates in several other metabolic processes were also significantly altered by ATF4 inhibition. Specifically, steady-state levels of fructose-1,6-bisphosphate were significantly increased by ATF4 knockdown, whereas other glycolytic intermediates such as 3-phosphoglycerate (3-PG) and pyruvate were decreased (Fig. 3K, L and Supplementary Fig. 5K). The TCA intermediates succinate and malate were also significantly reduced in shATF4-expressing NOMO-1 cells compared to control cells (Fig. 3M, N). Multiple metabolites related to nucleotide synthesis were also significantly altered by ATF4 inhibition, such as the pyrimidine synthesis intermediates uridine mono- and di-phosphate (UMP and UDP, respectively), as well as cytidine diphosphate (CDP) (Fig. 3O–Q). Steady-state levels of inosine monophosphate (IMP), adenosine mono- and di-phosphate (AMP and ADP, respectively), as well as guanosine diphosphate (GDP) were reduced upon ATF4 inhibition, but not guanosine monophosphate (GMP) (Fig. 3R–V). Lastly, sedoheptulose-7-phosphate (S7P), an intermediate in the pentose phosphate pathway (PPP), was significantly higher in shATF4-expressing NOMO-1 cells compared to control cells (Supplementary Fig. 5L). Collectively, these data indicate that ATF4 is required to maintain pools of multiple amino acids, TCA intermediates, and nucleotides.

### ATF4 inhibition impedes glucose-derived amino acid biosynthesis

**Fig. 4:**
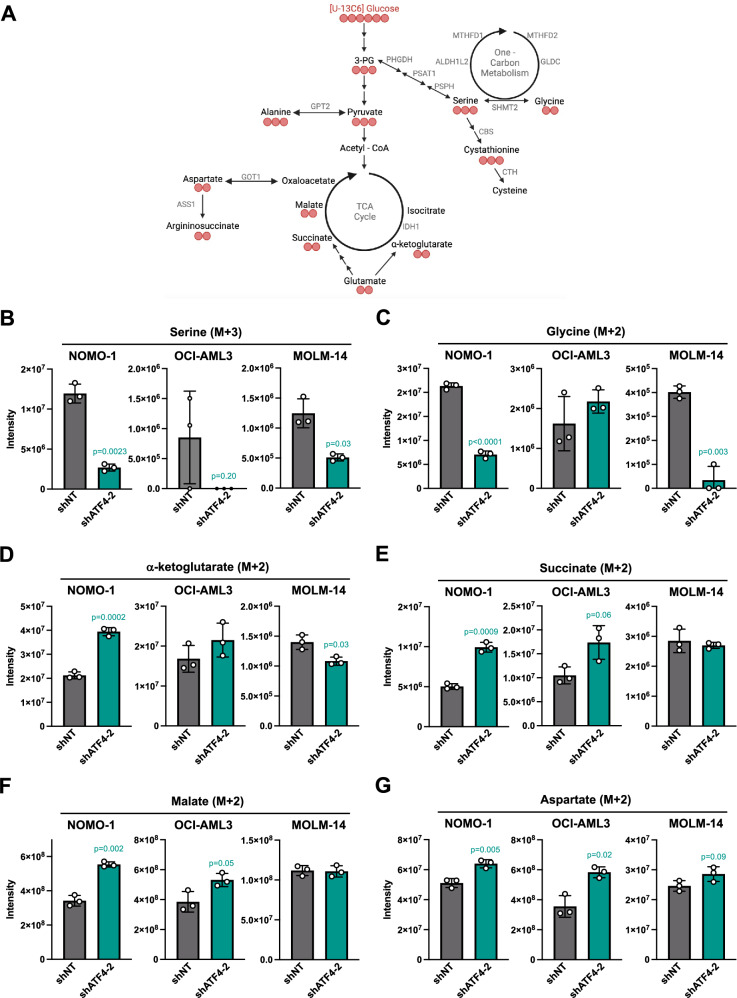
ATF4 disrupts serine metabolism and carbon flux to the TCA cycle. **A** Schematic of the interconnected metabolic pathways of U-^13^C-Glucose catabolism. Red circles represent ^13^C atoms in each metabolite that are derived from U-^13^C-Glucose (Created in BioRender. Basse, M. (2026) https://BioRender.com/ux53qtx). **B**–**G** Quantification of total levels of the indicated glucose-derived metabolites extracted from NOMO-1, OCI-AML3 or MOLM-14 cells from shNT and shATF4-2 conditions: **B** M + 3 Serine, **C** M + 2 glycine, **D** M + 2 α-KG, **E** M + 2 succinate, **F** M + 2 malate, and **G** M + 2 aspartate. Data represent the mean ± SD of three technical replicates. Two independent, biological replicates were performed for NOMO-1 cells and one independent, biological replicate for each OCI-AML3 and MOLM-14 cells. Statistics are derived from a two-sided, unpaired *t*-test (95% CI) comparing the shNT control and the shATF4-2 (teal *p*-values) condition. Adjustments for multiple comparisons were not made. Exact *p*-values are listed in the Source Data File. Source data for (**B**–**G**) are provided in the Source Data file.

We next investigated the role of ATF4 in glucose metabolism by performing isotopic tracing assays with a glucose isotopologue uniformly labeled with heavy carbons (U-^13^C-glucose) (Fig. 4A). Briefly, NOMO-1, OCI-AML3, and MOLM-14 cells expressing either shNT or shATF4-2 were cultured in U-^13^C-glucose-containing media and then subjected to HILIC-MS. The flux of glucose-derived carbons through glycolysis to pyruvate, was not substantially impacted by ATF4 inhibition in all three cell lines (Supplementary Fig. 6A). However, ATF4 knockdown did result in a significant increase in glucose-derived pyruvate (i.e., M + 3) in OCI-AML3 but not MOLM-14 and NOMO-1 cells (Supplementary Fig. 6B). The utilization of glucose-derived carbons in the PPP pathway, specifically into S7P and phosphoribosyl pyrophosphate (PRPP), was largely unimpacted by ATF4 inhibition (Supplementary Fig. 6C, D). The glucose-derived glycolytic intermediate 3-phosphoglyerate (3-PG) is used in the de novo synthesis of serine. While we did not observe substantial changes in the flux of glucose-derived carbons in the synthesis of 3-PG, we did find that ATF4 inhibition impeded glucose-derived carbons from being shunted into serine synthesis (Supplementary Fig. 6E, F). We also observed a decrease in steady-state levels of glucose-derived serine (M + 3) in all three cell lines upon ATF4 knockdown (Fig. 4B). This is consistent with the decrease in the expression of all three SSP enzymes in NOMO-1, OCI-AML3, and MOLM-14 cells expressing ATF4-targeting shRNAs (Fig. 2D and Supplementary Fig. 3A and B). As shown in Fig. 4A, serine can be converted into glycine by SHMT2 in the mitochondria and SHMT1 in the cytosol. ATF4 inhibition resulted in decreased expression of SHMT2, but not SHMT1, in NOMO-1 and MOLM-14 cells and to a lesser extent in OCI-AML3 cells (Supplementary Fig. 3B). Consistent with this observation, inhibition of ATF4 expression significantly reduced the incorporation of glucose-derived carbons into glycine steady-state levels, as well as steady-state levels of glucose-derived glycine in NOMO-1 and MOLM-14 cells but not OCI-AML3 cells (Fig. 4C and Supplementary Fig. 6G).

ATF4 inhibition also impacted the utilization of glucose-derived carbons into the TCA cycle. For example, levels of glucose-derived α-ketoglutarate (α-KG), succinate, malate, as well as the amino acids aspartate and glutamate were increased in NOMO-1 and OCI-AML3 cells expressing ATF4-targeting shRNAs compared to controls (Fig. 4D–G and Supplementary Fig. 6H). However, ATF4 knockdown minimally impacted the shunting of glucose-derived carbons into the TCA cycle in MOLM-14 cells (Fig. 4D–G and Supplementary Fig. 6H). Altogether, our glucose tracing data suggest that ATF4 influences how glucose-derived carbons are utilized in the biosynthesis of multiple amino acids and in the TCA cycle.

### ATF4 inhibition reduces glutamine anaplerosis

**Fig. 5:**
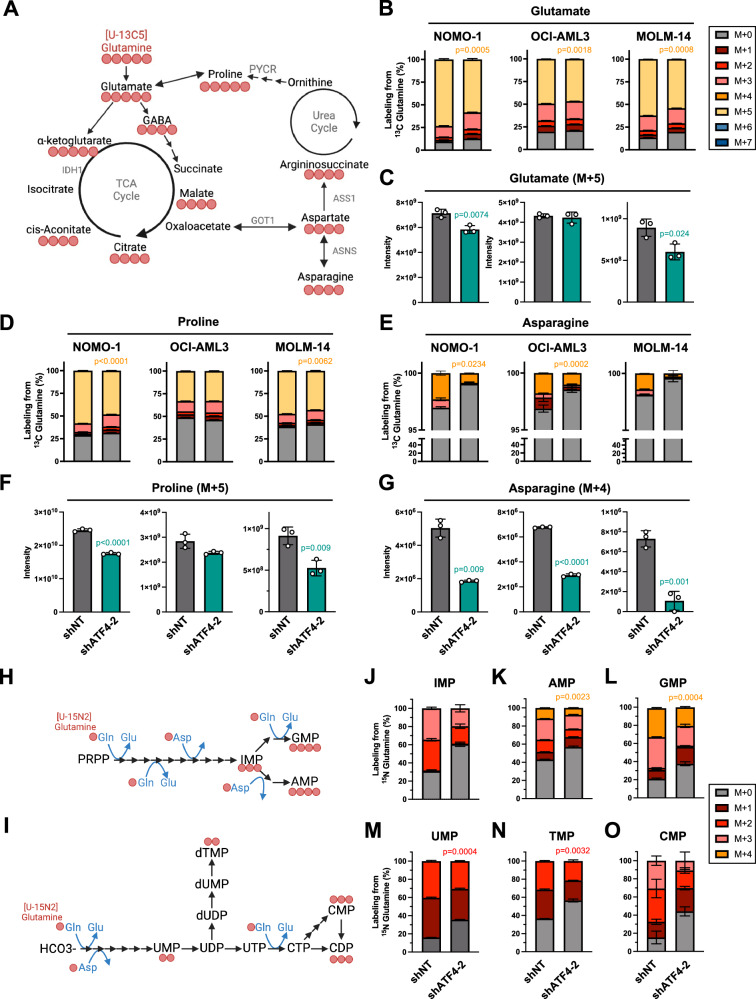
ATF4 regulates glutamine-derived amino acids and subsequent nucleotide synthesis. **A** Schematic of U-^13^C-Glutamine isotopologue catabolism. TPMs extracted from NOMO-1, OCI-AML3, or MOLM-14 cells from each shRNA condition were subjected to HILIC-MS (Created in BioRender. Basse, M. (2026) https://BioRender.com/2mdtpav). **B** Percent (%) U-^13^C-Glutamine isotopologue labeling of Glutamate. **C** Total levels of M + 5 Glutamate labeled from U-^13^C-Glutamine. **D**, **E** % U-^13^C-Glutamine isotopologue labeling of Proline (**D**) and Asparagine (**E**). **F**, **G** Total levels of M + 5 Proline (**F**) and M + 4 Asparagine (**G**) labeled from U-^13^C-Glutamine. For panels, **B**, **D**, and **E**, data represent the mean ± SD of three technical replicates. Two independent, biological replicates were performed for NOMO-1 cells and one independent, biological replicate for each OCI-AML3 and MOLM-14 cells. Statistics are derived from a two-way ANOVA (95% CI) comparison between the shNT control and shATF4-2 conditions. Šídák’s multiple comparisons test was applied to compare each label fraction between shNT and shATF4-2 conditions. *p*-values in (**B**, **D**) represent a comparison of the M + 5 label fraction between shNT and shATF4-2 conditions. *p*-values in (**E**) represent a comparison of the M + 4 label fraction between shNT and shATF4-2 conditions. Exact adjusted *p*-values are listed in the Source Data File. For panels (**C**, **F**, **G**), data represent the mean ± SD of three technical replicates. Two independent, biological replicates were performed for NOMO-1 cells and one independent, biological replicate for each OCI-AML3 and MOLM-14 cells. Statistics are derived from a two-sided, unpaired *t*-test (95% CI) comparing the shNT control and shATF4-2 (teal *p*-values) conditions. Adjustments for multiple comparisons were not made, and exact *p*-values are listed in the Source Data File. **H**, **I** Schematic of U-^15^*N*-Glutamine utilization in purine synthesis (**H**, Created in BioRender. Basse, M. (2026) https://BioRender.com/r80qvtn) and pyrimidine synthesis (**I**, Created in BioRender. Basse, M. (2026) https://BioRender.com/o5bv94e). Red circles represent ^15^N atoms derived from U-^15^*N*-Glutamine. **J**–**L** % U-^15^*N*-Glutamine isotopologue distribution of: IMP (**J**), AMP (**K**), and GMP (**L**). **M**–**O** % U-^15^*N*-Glutamine isotopologue distribution of: UMP (**M**), TMP (**N**), and CMP (**O**). For panels **J**–**L** and **M**–**O**, data represent the mean ± SD of three technical replicates of the one independent, biological replicate that was performed. Statistics are derived from a two-way ANOVA (95% CI) comparison between the shNT control and the shATF4-2 condition. Šídák’s multiple comparisons test was applied to compare each label fraction between shNT and shATF4-2 conditions. *p*-values in (**M**, **N**) represent a comparison of the M + 2 label fraction between shNT and shATF4-2 conditions. *p*-values in (**J**, **O**) represent a comparison of the M + 3 label fraction between shNT and shATF4-2 conditions. *p*-values in (**K**, **L**) represent a comparison of the M + 4 label fraction between shNT and shATF4-2 conditions. Source data for (**B**–**G**, **J**–**L**, **M**–**O**) are provided in the Source Data file.

Glutamine-derived carbons are utilized in anaplerotic reactions to replenish TCA intermediate pools and support the biosynthesis of certain amino acids (Fig. 5A)^37^. To assess the role of ATF4 in glutamine anaplerosis, we performed isotopic tracing with a uniformly labeled carbon glutamine isotopologue (U-^13^C-glutamine) in NOMO-1, OCI-AML3, and MOLM-14 cells expressing either shNT- and shATF4-2-targeting shRNAs. Although ATF4 has been previously shown to regulate glutamine uptake^38–40^, we did not observe any difference in glutamine import between control and ATF4 knockdown cells (Supplementary Fig. 7A). Glutamine conversion into glutamate was significantly impeded by ATF4 inhibition in all three cell lines, though steady-state levels of glutamine-derived glutamate (M + 5) were significantly reduced in NOMO-1 and MOLM-14 cells but not OCI-AML3 (Fig. 5B, C). Glutamate contributes to the synthesis of α-KG (M + 5). Compared to their respective controls, NOMO-1, OCI-AML3, and MOLM-14 cells expressing shATF4-2 displayed a significant reduction of glutamine-derived carbons being incorporated into either α-KG (Supplementary Fig. 7B). We also observed fewer glutamine-derived carbons being incorporated into the production of succinate in NOMO-1 cells and malate in NOMO-1 and MOLM-14 cells (Supplementary Fig. 7C, D). ATF4 knockdown also trended towards a decline of total glutamine-derived succinate and malate in NOMO-1 and MOLM-14 but an increase in both in OCI-AML3 cells (Supplementary Fig. 7E and F). ATF4 inhibition also significantly altered the biosynthesis of several glutamine-derived amino acids. For instance, the incorporation of glutamine-derived carbons into the production of proline and asparagine was reduced by ATF4 knockdown in NOMO-1, OCI-AML3, and MOLM-14 (Fig. 5D, E). ATF4 inhibition also resulted in decreased levels of proline (M + 5), asparagine (M + 4) in NOMO-1, OCI-AML3, and MOLM-14 (Fig. 5F, G). NOMO-1 and MOLM-14 cells expressing shATF2-targeting shRNAs displayed reduced incorporation of glutamine-derived carbons into aspartate synthesis, however, ATF4 knockdown only reduced aspartate (M + 4) levels in MOLM-14 cells (Supplementary Fig. 7G, H). The incorporation of glutamine-derived carbons being used to synthesize argininosuccinate, as well as total glutamine-derived argininosuccinate levels, were also significantly impeded by ATF4 knockdown in NOMO-1 but not in OCI-AML3 or MOLM-14 cells (Supplementary Fig. 7I, J).

Glutamine plays a critical role in the synthesis of nucleotides. For example, glutamine-derived carbons are used to synthesize the pyrimidine precursor orotidine, which is subsequently used to generate UDP. The incorporation of glutamine-derived carbons into orotidine and UDP synthesis was significantly reduced by ATF4 knockdown in NOMO-1 and MOLM-14 cells and a similar trend in OCI-AML3 (Supplementary Fig. 8A, B). As a result, total glutamine-derived orotidine (M + 3) and UDP (M + 3) levels were also significantly impaired by ATF4 knockdown in NOMO-1 and MOLM-14 cells (Supplementary Fig. 8C, D). Glutamine is a central source of nitrogen in both purine (Fig. 5H) and pyrimidine synthesis (Fig. 5I and Supplementary Fig. 8E). To further explore the role of ATF4 in nucleotide synthesis, we performed isotopic tracing assays in control and experimental shRNA conditions with a glutamine isotopologue containing two heavy nitrogen atoms (U-^15^*N*-glutamine). ATF4 inhibition impaired U-^15^*N*-glutamine labeling of purines AMP and GMP (but not IMP), as well as the pyrimidine UMP in NOMO-1 cells (Fig. 5J–M). UMP is subsequently converted into UDP, which is an important precursor to thymidine- and cytidine-derived pyrimidine metabolites (Fig. 5I). Although UDP was not detected in this analysis, multiple downstream derivatives were altered in shATF4-2-expressing cells compared to controls. First, ATF4 inhibition reduced (though not significantly) UDP-*N*-acetylglucosamine labeling, which obtains an additional glutamine-derived nitrogen from *N*-acetylglucosamine-1-phosphate during glucosamine synthesis (Supplementary Fig. 8E, F). Second, ATF4 inhibition reduced U-^15^*N*-glutamine labeling of thymidine monophosphate (TMP) and dTTP (Fig. 5N and Supplementary Fig. 8G). Third, ATF4 inhibition also significantly reduced U-^15^*N*-glutamine-derived labeling of CDP, but not CMP (Fig. 5O and Supplementary Fig. 8H). Despite the impact of ATF4 inhibition on the incorporation of glutamine-derived nitrogen atoms into nucleotide synthesis, shATF4-2-expressing cells showed only a marginal but significant reduction in many of the enzymes that catalyze these reactions (e.g., PPAT, PFAS, PAICS, and CAD) compared to control cells (Supplementary Fig. 8I). These combined glutamine tracing studies underscore the supportive role of ATF in nucleotide synthesis in AML cells.

### ATF4 regulates tRNA aminoacylation

We next tested whether exogenous amino acid supplementation was able to mitigate the cellular consequences of ATF4 inhibition. To this end, NOMO-1, OCI-AML3, and MV4-11 cells expressing either control or ATF4-targeting shRNAs were cultured in media containing either standard (1×), 2-fold (2×), or 5-fold (5×) higher levels of the amino acids that were significantly altered by ATF4 inhibition (e.g., glycine, arginine, asparagine, aspartate, cysteine, glutamate, glutamine, proline, and serine). Increasing concentrations of these amino acids were unable to mitigate the anti-leukemia effects of ATF4 inhibition in NOMO-1, OCI-AML3, or MV4-11 cells (Supplementary Fig. 9A–C), suggesting that ATF4 supports AML cell biology through mechanisms additional to promoting amino acid biosynthesis.

**Fig. 6:**
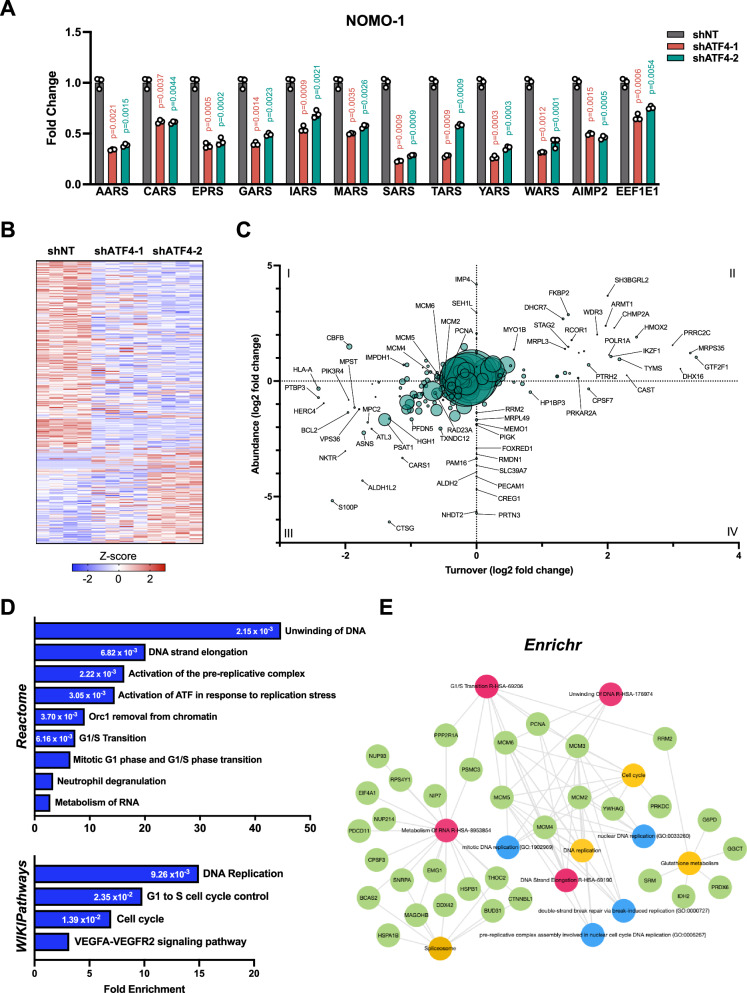
ATF4 inhibition selectively alters the translation of select proteins involved in DNA replication and AML. **A** qPCR analysis of the indicated tRNA aminoacylation mRNAs in DOX-treated NOMO-1 cells from each shRNA condition. Data represent the mean ± SD of three technical replicates from one biological replicate of two independent, biological replicates. Statistics are derived from a two-sided, unpaired *t*-test comparing the shNT control with the shATF4-1 (pink *p*-values) or shATF4-2 (teal *p*-values) conditions. Adjustments for multiple comparisons were not made, and exact *p*-values are listed in the Source Data File. **B** Heatmap of proteins that were significantly differentially labeled (*p* < 0.10) in ATF4-2-shRNA-expressing NOMO-1 cells compared with shNT controls. **C** Protein turnover and abundance correlation of significantly differentially labeled proteins in ATF4-2-shRNA-expressing NOMO-1 cells compared with shNT controls. The average abundance of each protein is represented by the size of the corresponding circle. **D** Pathway enrichment analysis of proteins with significantly decreased turnover using the DAVID. The analysis was performed using Reactome (*top panels*) and Wiki (*bottom panels*) pathways. Benjamini scores are shown in white within the bar graph. **E** Clustering analysis of proteins that displayed a significant decrease in labeling upon ATF4 inhibition using the Enrichr bioinformatic resource. Source data for all panels are provided in the Source Data file.

In response to certain stresses such as nutrient deprivation or ER stress, ATF4 trans-activates multiple cytosolic tRNA synthetases, which catalyze the aminoacylation of tRNA, often referred to as tRNA “charging” or “loading”^10,11,41^. Transcripts encoding enzymes that catalyze cytosolic tRNA aminoacylation were significantly decreased in NOMO-1 cells expressing shATF4-1 or shATF4-2 compared to control cells (Fig. 2B, C). qPCR validation confirmed that inhibition of ATF4 in NOMO-1 cells significantly decreased the expression of several aminoacyl-tRNA synthetases, as well as AIMP2 and EEF1E1, two proteins involved in the formation of the aminoacyl-tRNA synthetase complex (Fig. 6A). The gene expression of many of these aminoacyl-tRNA synthetases was also found to be decreased by ATF4 inhibition in OCI-AML3 and MV4-11 cells (Supplementary Fig. 9D, E). Several of the aminoacyl-tRNA synthetases whose expression was impacted by ATF4 inhibition corresponded to amino acids that were also decreased by ATF4 inhibition (Fig. 3B), including alanine, cysteine, glutamate, proline, glycine, and serine (Supplementary Fig. 9F). These results suggest that ATF4, through regulation of tRNA synthetase expression, may regulate protein synthesis.

### ATF4 inhibition affects the translation of select proteins

To investigate whether ATF4 inhibition impacts protein synthesis, we next employed stable isotope labeling by amino acids in cell culture (SILAC) proteomics. Specifically, NOMO-1 cells expressing shNT, shATF4-1, or shATF4-2 were treated with DOX for 24 h and subsequently cultured in media supplemented with heavy isotopologues of lysine (U-^13^C-lysine) and arginine (U-^13^C-arginine) for an additional 24 h – prior to the changes in AML cell cycling mediated by ATF4 inhibition. Protein extracts from each condition were then subjected to high-performance liquid chromatography mass spectrometry (HPLC-MS) to quantify protein abundance and heavy isotope labeling. A total of 2,918 proteins were detected regardless of heavy amino acid labeling. Principal component analysis of these proteins showed that replicates of shNT, shATF4-1, and shATF4-2 conditions clustered together, and shATF4-1 and shATF4-2 replicates clustered closer together than shNT replicates (Supplementary Fig. 10A). Inhibition of ATF4 led to a significant difference in the abundance of 111 proteins – 61 proteins increased and 50 proteins decreased – compared with shNT controls (<0.05) (Supplementary Fig. 10B). Expression of 53 of the 111 differentially expressed proteins (28 that decreased) correlated with changes in mRNA expression mediated by ATF4 inhibition suggesting that these are direct transcriptional targets of ATF4 (Supplementary Fig. 10C). In fact, many of the 28 proteins that were decreased at both the mRNA and protein level by ATF4 inhibition are involved in amino acid metabolism and tRNA aminoacylation (Supplementary Fig. 10D). Of the 50 significantly decreased proteins, only 28 (56%) of could be explained by decreased RNA expression.

A total of 2,888 of all detected proteins (99%) displayed SILAC labeling. No significant difference in total labeling was observed between shRNA conditions, indicating that ATF4 inhibition does not alter global protein turnover (Supplementary Fig. 11A). However, 331 proteins were identified to be differently labeled between shNT- and shATF4-expressing conditions using a one-tailed cut-off. Of these 331 proteins, 198 displayed decreased labeling in ATF4-shRNA-expressing NOMO-1 cells compared with shNT controls, whereas 133 proteins showed increased turnover (Fig. 6B). Next, we removed proteins whose average fold change turnover went in opposite directions between the shATF4-1 and shATF4-2 (e.g., up in one shRNA and down in the other), which trimmed the 331-protein list to 246. Further confirming that ATF4 does not regulate global protein translation, only 8.4% of detected labeled proteins displayed a significant change in protein turnover upon ATF4 inhibition.

To better understand the protein turnover alterations mediated by ATF4 inhibition, the 246 proteins were plotted as abundance versus labeling. Many of these proteins fall along a line of best fit that crosses through quadrants II and III (Supplementary Fig. 11B), indicating that these proteins are affected by a translational defect rather than an increase in stability (proteins in quadrant I) or active degradation (quadrant IV). After accounting for the average protein abundance of each protein, it was observed that many of the highly abundant proteins that were affected by ATF4 inhibition were in quadrant III, which is representative of decreased translation (Fig. 6C and Supplementary Fig. 11C). Those that fell in quadrant II, indicative of increased translation, consisted of less abundant proteins, which are more susceptible to skewed results due to low abundance. The 246-protein list was filtered to retain proteins that did not show a significant difference in RNA expression with ATF4 inhibition or had a disproportionate decrease in average turnover compared to RNA expression fold change. This reduced the number of proteins down to 172, from which 125 showed a significant decrease in translation. An enrichment analysis of the 125 proteins whose translation is selectively decreased by ATF4 inhibition, showed that these proteins are enriched in pathways associated with the cell cycle, G1/S transition, and DNA replication (Fig. 6D and Supplementary Data 5). To gain further insight into the functions of these 125 proteins, two separate clustering analyses were performed, one using StringDB^42^ and the other with Enrichr^43^. StringDB identified multiple nodes of interactions among the majority of proteins (Supplementary Fig. 11D). Enrichr found that pathways associated with the cell cycle, G1/S transition, DNA replication, unwinding of DNA, and the pre-replication complex were enriched among the 125 proteins, as well as proteins that regulate splicing and glutathione metabolism (Fig. 6E).

### DNA replication speed and timing are altered by ATF4 inhibition

**Fig. 7:**
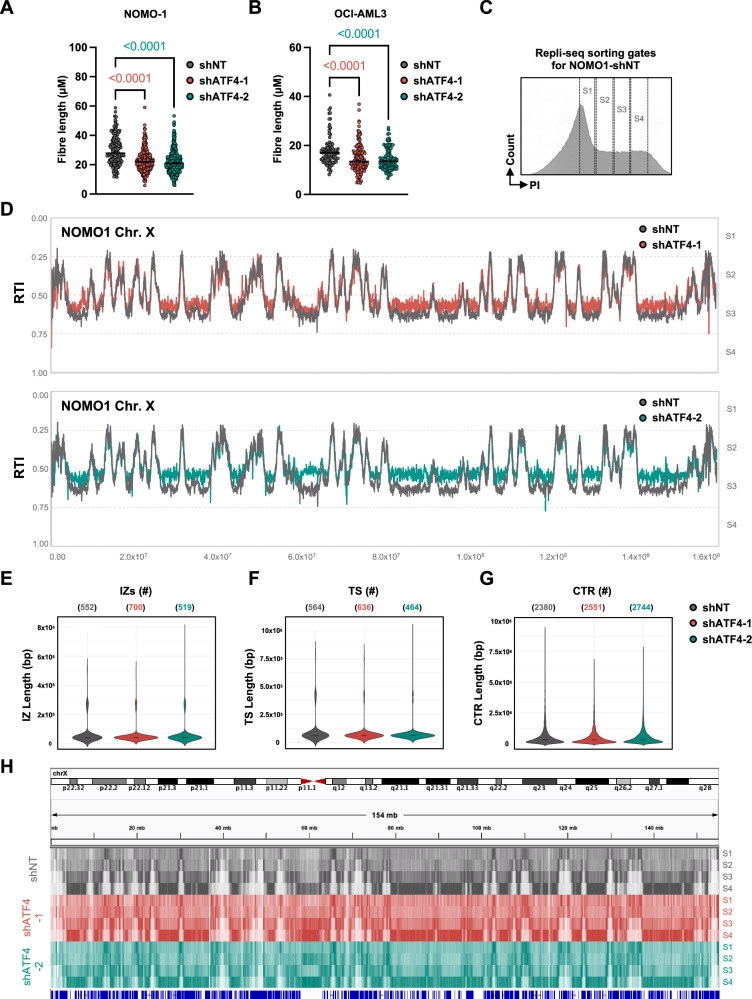
DNA replication speed and timing are altered by ATF4 inhibition. **A**, **B** Measurement of DNA fiber lengths (*n*) from DOX-treated: **A** NOMO-1 cells expressing shNT (*n* = 200), shATF4-1 (*n* = 364), or shATF4-2 (*n* = 436), or **B** OCI-AML3 cells expressing shNT (*n* = 108), shATF4-1 (*n* = 147), or shATF4-2 (*n* = 103). For **A**, **B** all graphed data represent the mean ± SD of three technical replicates from one biological replicate. Three independent, biological replicates were carried out. Statistics are derived from a one-way ANOVA between shNT (control), shATF4-1 (pink *p*-values), and shATF4-2 (teal *p*-values) conditions. A Dunnett’s multiple comparisons test (95% CI) was also performed, and adjusted *p*-values are listed in the figures. **C** FACS gating strategy used for isolating the various stages of S-phase (S1-S4) for Repli-seq analysis. Representative plot of NOMO1 cells expressing shNT controls. **D** Quantitative tracks of RTI (calculated from the S1-S4 Repli-seq) of the X chromosome from NOMO-1 cells expressing shNT versus those expressing shATF4-1 (*top*) or NOMO-1 cells expressing shNT versus those expressing shATF4-2 (*bottom*). **E**–**G** Lengths of IZs (**E**), TSs (**F**), and CTRs (**G**) calculated from Repli-seq signals from NOMO-1-shNT, -shATF4-1, and -shATF4-2 cells. **H** IGV-based visualization of genomic tracks of the X chromosome with S1–S4 Repli-seq signals from NOMO-1-shNT, -shATF4-1, and -shATF4-2 cells. Source data for all panels are provided in the Source Data file.

The selective decline in the synthesis of proteins involved in the initiation and firing of DNA replication prompted us to assess how ATF4 inhibition impacted DNA replication. First, we performed DNA fiber analyses^26^ on NOMO-1 and OCI-AML3 cells expressing control or ATF4 targeting shRNAs. Briefly, cells from each condition were incubated with the thymidine nucleoside-analog 5’-Chloro-2’-deoxyuridine (CldU, 50 μM) for 30 min, followed by an additional 30-min incubation with a second thymidine-analog, 5’-Ido-2’-deoxyuridine (IdU, 250 μM). Extracted DNA fibers from each condition were then assessed for length. In both OCI-AML3 and NOMO-1 cells, ATF4 inhibition resulted in significantly shorter fiber lengths, indicating a reduction in DNA replication speed (Fig. 7A, B).

To further investigate the role of ATF4 in DNA replication, we conducted a Repli-seq analysis^44,45^ on NOMO-1 and OCI-AML3 cells expressing control or ATF4-targeting shRNAs. Cells from each shRNA condition were pulsed with 5’-Bromo-2’-deoxyuridine (BrdU, 100 μM), then fixed and stained with propidium iodide (PI). Stained cells were subjected to fluorescence-activated cell sorting (FACS) to isolate cells at different stages of S-phase (S1–S4) (Fig. 7C and Supplementary Fig. 12A). Nascent BrdU-labeled DNA was isolated from each fraction and subjected to next-generation sequencing followed by alignment with the hg38 genome. Utilizing a previously established computational workflow^46^, we next calculated the replication timing index (RTI) of each condition. The most profound alterations in RTI by ATF4 inhibition, which were largely regions of premature DNA replication, occurred on the X chromosome of both NOMO-1 and OCI-AML3 cells (Fig. 7D and Supplementary Fig. 12B). Although global RTI patterns across the autosomal chromosomes were less pronounced, regional alterations of RTI were common in cells expressing ATF4-targeting shRNAs in both directions, with early replicating regions delayed and late replicating regions undergoing earlier replication (Supplementary Figs. 13–18). For example, replication timing at the 108.8 Mb region of chromosome 4 was delayed by ATF4 inhibition, whereas replication timing at the 63.5 Mb region of chromosome 8 occurred earlier in NOMO-1 cells (Supplementary Fig. 12C). In NOMO-1 cells, ATF4 inhibition altered replication timing—as defined by an RTI differential >0.05, as shown in ref. ^45^—across one-fifth of the genome (21.18% for shATF4-1 and 26.2% for shATF4-2). Of these altered RTI regions, 11.46% were consistently and similarly affected by both shRNAs, with 60.65% showing delayed replication. In OCI-AML3, 18.82% of the genome displayed differential RTI caused by ATF4 knockdown, 63.25% of which underwent delayed replication. The common loci with increased or decreased RTI caused by ATF4 knockdown shared between OCI-AML3 and NOMO-1 account for 2.7% of the genome, likely reflecting inherent differences in RTI profiles between unperturbed cell lines. Overall, these results indicate that ATF4 inhibition disrupts regional DNA replication timing by either initiating replication ahead of schedule or delaying replication firing.

We next investigated the impact of ATF4 inhibition on regions or zones where DNA replication is more likely to be initiated (i.e., initiation zones or IZs). Although the number of IZs was largely unaffected by ATF4 inhibition, NOMO-1 cells expressing ATF4-targeting shRNAs displayed shorter IZ lengths compared to control cells (shNT_mean_ = 80435 bp, shATF4-1_mean_ = 66286 bp, shATF4-2_mean_ = 62717 bp) (Fig. 7E). A similar trend of shorter IZs was observed between OCI-AML3 cells expressing control or ATF4-targeting shRNAs (shNT_mean_ = 78437 bp, shATF4-2_mean_ = 73433 bp) (Supplementary Fig. 12D). These data indicate that ATF4 inhibition is disrupting replication origin firing. Similarly, the length of DNA replication termination sites (TSs) tended to be shorter in leukemia cells expressing ATF4-targeting shRNAs compared to controls in both NOMO-1 (shNT_mean_ = 84043 bp, shATF4-1_mean_ = 68504 bp, shATF4-2_mean_ = 74353 bp) and OCI-AML3 cells (shNT_mean_ = 85989 bp, shATF4-2_mean_ = 75397 bp) (Fig. 7F and Supplementary Fig. 12E). The shorter TSs could indicate that ATF4 is required for proper termination of DNA replication possibly by regulating replication fork speed or stability. Constant timing regions (CTR) are large regions of DNA that are replicated by the coordinated firing of multiple origins of replication (i.e., IZs) and can occur in both early and late stages of DNA replication. Further highlighting the requirement of ATF4 to maintain proper DNA replication timing, ATF4 inhibition also significantly altered the length of CTR in both NOMO-1 (shNT_mean_ = 663298 bp, shATF4-1_mean_ = 550412 bp, shATF4-2_mean_ = 532598 bp) and OCI-AML3 cells (shNT_mean_ = 723622 bp, shATF4-2_mean_ = 664257 bp) (Fig. 7G and Supplementary Fig. 12F). These subtle yet widespread alterations in DNA replication zones were also visually apparent from stacking the S1–S4 genomic tracks (Fig. 7H and Supplementary Figs. 12G and 13–18). Collectively, these data indicate that ATF4 plays a critical role in coordinating the production of certain amino acids and nucleotides with selective protein synthesis to facilitate the G1-to-S-phase transition and ensure the proper timing of DNA replication in leukemia cells.

## Discussion

The eukaryotic cell cycle is a tightly orchestrated, unidirectional process that consists of discrete stages and checkpoints. The transition from the G1- to the S-phase requires the coordinated ramping up of multiple biosynthetic processes, such as amino acid, nucleotide, and protein synthesis, in order to generate the biomass needed for DNA replication. In particular, tumor cells need to amplify the output of these biosynthetic processes to maintain their accelerated growth state. To do so, cancer cells often co-opt and engage the upstream regulators that choreograph these anabolic processes. Here, we report that AML cells rely on ATF4 to engage transcriptional programs that promote amino acid biosynthesis and tRNA charging to support de novo nucleotide synthesis, glutamine anaplerosis, and the preferential translation of proteins that control the timing and rate of DNA replication.

ATF4 has been extensively studied in response to cellular stimuli, but it is often constitutively activated in many tumor settings, such as NSCLC, CRC, fibrosarcoma, and AML. ATF4 promotes the transcription of an array of target genes, several irrespective of stimuli, but many in response to a specific stress. For example, Torrence et al. demonstrated that ATF4 regulates both unique and shared transcriptional targets in cells treated with insulin versus those exposed to ER stressors^15^. ATF4 has also been shown to drive these anabolic-centric transcriptional programs in response to other cellular stresses, such as the withdrawal of individual amino acids (e.g., leucine, asparagine, histidine, serine, glutamine, and arginine), or disruption of the TCA cycle across multiple cell types, suggesting that these are canonical ATF4 target genes^9–11,13,14,47^. ATF4 also regulates the basal transcription of a few canonical ATF4-targets, including SSP enzymes and ASNS in certain tissues^19,21,25^. Here, we report that in unstressed AML cells, ATF4 regulates the basal expression of canonical ATF4-target genes that encode enzymes that regulate biosynthesis, import, and tRNA charging of numerous amino acids, as well as several previously described stress-specific ATF4 target genes (e.g., *HSPA5, HERPUD1, XBP1, ATF5, DDIT3*, and *EIF2A*)^48^.

Although several studies have shown that ATF4 regulates the transcription of multiple amino acid biosynthesis enzymes, only a handful have assessed the direct impact of ATF4 inhibition on total levels and synthesis rates of amino acids, as well as global intracellular metabolism^11,14,21^. By combining transcriptomic and metabolomic approaches, we have made several key observations regarding how ATF4 manages intracellular metabolism. First, through transcriptional regulation of serine biosynthetic enzymes, ATF4 controls total and glucose-derived levels of serine. Moreover, ATF4 promotes the transcription of the aspartate-regulatory enzyme GOT1 to maintain steady-state levels of aspartate. Second, ATF4 inhibition diverts glucose-derived carbons into TCA intermediates such as α-KG, succinate, and malate. Third, we have observed that ATF4 is required for glutamine anaplerosis, specifically supporting the utilization of glutamine to maintain the biosynthesis of amino acids such as glutamate, proline, asparagine, argininosuccinate, and aspartate. However, in contrast with previous studies^49,50^, we found that ATF4 inhibition diminishes glutamine utilization independent of substantial changes in the expression of glutamine anaplerotic enzymes or glutamine import. Whether the decline in glutamine anaplerosis is connected to the increased flux of glucose-derived carbons into the TCA cycle remains to be determined. Lastly, we find that ATF4 is required to maintain purine and pyrimidine nucleotide pools. For example, serine plays a crucial role in the IMP synthesis pathway, and cells expressing ATF4-targeting shRNAs displayed a significant reduction in both serine and IMP. Furthermore, ATF4 inhibition decreased pools of purines and pyrimidines, as a result of reduced glutamine-derived nitrogen utilization in the de novo synthesis of both purines and pyrimidines. These observations highlight the importance of ATF4 in coordinating multiple amino acid biosynthetic processes to maintain nucleotide pools. However, tumor cells display extensive metabolic plasticity that is, in part, influenced by extracellular nutrient availability; therefore, how ATF4 regulates intracellular metabolism could vary between culture media formulations, as well as between in vitro and in vivo settings.

ATF4 is a well-established transcriptional regulator of genes encoding cytosolic tRNA synthetases, which catalyze the transesterification of the appropriate amino acid onto its corresponding cognate tRNA, utilizing an ATP molecule to form an amino acyl tRNA^10,11,13^. Given the central role of charged-tRNAs in protein synthesis, a decrease in available charged tRNAs is anticipated to have global effects on protein synthesis. Han et al.^47^ demonstrated that ATF4 over-expression increased ^35^*S*-methionine/cysteine protein incorporation in MEFs, whereas Torrence et al.^15^ showed that *Atf4* null MEFs display decreased ^35^*S*-methionine-labeling of proteins. In our experimental systems, inhibition of ATF4 decreased the expression of multiple tRNA synthetases, including MARS and CARS, which catalyze the formation of methionine- and cysteine-charged amino acids, respectively. However, ATF4 knockdown did not impact the expression of lysine (i.e., KARS) or arginine (RARS) tRNA ligases. Therefore, we performed SILAC labeling experiments using heavy isotope versions of lysine and arginine, to assess how ATF4 inhibition impacted protein synthesis without a potential confounding effect of altered tRNA synthetase expression. Although ATF4 inhibition did not significantly impact global translation, we did identify a portion of proteins whose synthesis was significantly decreased (without a concomitant decrease in mRNA levels) upon ATF4 inhibition. Many of these selectively translated proteins directly influence DNA replication, particularly MCM2-6, PCNA, and RRM2. We also found an enrichment of proteins related to RNA splicing and glutathione metabolism, both important for the G1/S transition. The translation of several other notable proteins was decreased, including: (1) CBFB, a critical transcription factor that plays a key role in leukemogenesis; (2) IDH2, mutated in AML; (3) BCL-2, an anti-apoptotic target of venetoclax; and (4) multiple mitochondrial proteins. It is important to note that due to the limited capabilities of current mass spectroscopy technology, we were only able to detect about 15% of the proteome in our proteomic studies. Thus, it is possible that the translation and abundance of many other proteins, possibly not related to the G1/S transition, are also impacted by ATF4 inhibition. Nonetheless, in our experimental setting, ATF4 inhibition did not alter the global synthesis of detected proteins, but selectively impacted proteins that are largely associated with DNA replication and AML biology.

Given the decline in DNA replication initiation machinery upon ATF4 knockdown, we carried out Repli-seq and DNA fiber assays, to assess the role of ATF4 in DNA replication timing and speed. While inhibition of ATF4 did not substantially alter the number of replication origins, the rate and proper timing of DNA replication in specific regions throughout the genome were substantially altered by ATF4 knockdown. The most notable RTI alterations between control and ATF4 shRNAs were observed at the X chromosome, as well as chromosomes 7, 10, 12, 14, and 20, where ATF4 inhibition resulted in advanced replication timing in regions of late DNA replication (Fig. 7D and Supplementary Fig. 12B, 14C, 15B, 15D, 16B, and 17D), which are enriched for heterochromatin^51,52^. This is consistent with previous studies that have shown that chemically-induced replication stress advances replication timing at late-replicating, heterochromatic-rich regions of chromsomes^53^. Our proteomic analyses show that ATF4 inhibition leads to a selective decrease in the translation of multiple components of the minichromosome maintenance (MCM) complex (Fig. 6C, E). The MCM complex localizes to canonical (i.e., ORC-bound) origins of DNA replication to unwind DNA and allow for strand duplication. In mammalian cells, particularly in the setting of human cancer, excess components of the MCM complex can also initiate DNA replication from alternative (or ‘dormant’) replication origins to overcome replication stress^54–56^, such as fork stalling that can arise from nucleotide pool depletion. Therefore, leukemia cells may utilize ATF4 to initiate DNA replication from both canonical and alternative origins of replication. Different regions of the genome replicate at distinct time points throughout the S-phase, and improper replication timing can lead to genomic instability and alterations in cell fate. For example, disruption in replication timing has been shown to drive the terminal differentiation of AML cells^57^. Consistent with this observation, we found that alterations in DNA replication mediated by ATF4 inhibition cause leukemia cells to adopt expression patterns (i.e., increased CD11b expression) and morphological characteristics of myeloid maturation, indicating that ATF4 indirectly maintains the differentiation blockade in acute leukemia by ensuring proper DNA replication timing.

Our findings demonstrate that ATF4 regulates DNA replication timing and origin dynamics by coupling metabolic state with DNA synthesis. This suggests that ATF4 safeguards S-phase progression through coordinated control of amino acid biosynthesis, nucleotide biosynthesis, and replication origin architecture. It is important to note that AML is a genetically complex disease that can arise from numerous distinct genetic abnormalities or combinations thereof. Therefore, while our data and others indicate that ATF4 is frequently up-regulated across a wide spectrum of genetic subtypes of AML, it remains possible that the molecular role of ATF4 could vary among different genetic subtypes. Regardless, our data provide unique insights into the roles of ATF4 in cancer biology and highlight how transcriptional regulators can coordinate multiple biosynthetic processes to dictate cell fate.

## Methods

### Cell culture

The following human AML cell lines were obtained from the German Collection of Microorganisms and Cell Cultures (DSMZ), catalog numbers in parentheses: NOMO-1 (ACC 542), OCI-AML3 (ACC 582), OCI-AML2 (ACC 99), and MOLM-14 (ACC 777). The following human AML cell lines were obtained from the American Type Culture Collection (ATCC), catalog numbers in parentheses: K-562 (CCL-243) and MV4-11 (CRL-9591). Cells were cultured in the recommended media conditions as instructed by the distributor’s instructions and as we have done before refs. ^24,28^. Murine AML cells were cultured in RPMI-1640 supplemented with 10% FBS and penicillin/streptomycin, 10 ng/ml mSCF (Peprotech), 6 ng/ml mIL-6 (Peprotech), and 5 ng/ml mIL-3 (Peprotech), hereafter referred to as cytokine-enriched media (CEM). Cell line authentication was carried out by IDEXX using the short tandem repeat (STAR) profiling method.

### Patient-derived AML samples

The de-identified patient-derived AML samples were obtained from the Hematology Tissue Bank at the Ohio State University Comprehensive Cancer Center, and studies were approved by the Institutional Review Board (IRB) of Fox Chase Cancer Center, under the IRB protocol 16-9037. All human studies were conducted in accordance with the “Declaration of Helsinki” principles. Thawed PD-AML cells were plated on irradiated mono-layers of HS-27 cells with Stemspan (Stem Cell Technology) supplemented with 10% FBS, 100 ng/ml hSCF, 100 ng/ml hFLT3 ligand, 20 ng/ml hIL-3, 20 ng/ml hIL-6, 20 ng/ml G-CSF (cytokines from Peprotech), and 1 µM StemRegenin 1 (Stem Cell Technology)^26–28^. Transduced cells from each condition were evaluated for GFP expression every 3 days for 15 days. Due to the limited, non-renewable availability of PD-AML cells, experiments were conducted once, and all tissue samples used were destroyed during or after the analysis.

### Doxycycline-inducible lentiviral transduction

Dharmacon™ SMARTvector™ Inducible Lentiviral shRNAs co-expressing RFP in a mEF1a vector were obtained from Horizon Discovery (Catalog #: V3SH11252-228497555 for shATF4-1, V3SH11252-229558472 for shATF4-2, and VSC6575 for shNT). Lentiviruses were packaged in 293TL cells by co-transfection for 48 h with the pSPAX and pMD vectors. AML cells were then transduced with lentiviruses for 48 h, and cells expressing the appropriate vector containing puromycin resistance were selected in RPMI-1640 media supplemented with 2.0 μg/mL puromycin for 5 days. Single cell clones were then generated by seeding puromycin-selected cells in a 96-well plate at a concentration of 0.7 cells/well, allowed to grow for several weeks, and then selected for clones that expressed greater than 90% RFP efficiency after 48 h of DOX induction with RPMI-1640 media supplemented with 2.0 μg/mL DOX.

### Cell proliferation assay

Cell proliferation was assessed daily utilizing CellTiter 96® AQueous One Solution Cell Proliferation Assay (Promega) according to the manufacturer’s instructions. Cell proliferation rates were calculated by taking the optical density measurements of each day and normalizing to the Day 0 optical density measurement. Three to five independent biology replicates were conducted for each cell line, and the graphed data represent the mean ± SD of three technical replicates of one biological replicate. An unpaired, two-tailed *t*-tests were applied to compare control and experimental shRNA conditions.

### RNA-seq

Total RNA was extracted using the Quick-RNA Miniprep Kit (Zymo Research). RNA Integrity Number and purity were assessed by Agilent 2100 Bioanalyzer (Agilent) and NanoDrop™, respectively, prior to submitting samples with a RIN ≥ 9 to Novogene for eukaryotic RNA-Seq. At Novogene, messenger RNA was purified from total RNA using poly-T oligo-attached magnetic beads. After fragmentation, the first strand cDNA was synthesized using random hexamer primers, followed by the second strand cDNA synthesis using dTTP for a non-directional library. The library then underwent end repair, A-tailing, adapter ligation, size selection, amplification, and purification. The library was checked with Qubit and real-time PCR for quantification and a bioanalyzer for size distribution detection. Quantified libraries were then pooled and sequenced on Illumina HiSeq 2500 using a 150 bp paired-end sequencing strategy, according to effective library concentration and data amount. Raw data (raw reads) of fastq format were first processed through in-house Perl scripts. In this step, clean data (clean reads) were obtained by removing reads containing adapters, reads containing ploy-N, and low-quality reads from raw data. At the same time, Q20, Q30, and GC content of the clean data were calculated, which showed an average Q20 of 98.2% and Q30 of 94.8%, ensuring high-quality reads. All downstream analyses were based on the clean data with high quality. The reference genome, GRCh38, and gene model annotation files were downloaded from the genome website directly. An index of the reference genome was built using Hisat2 v2.0.5, and paired-end clean reads were aligned to the reference genome using Hisat2 v2.0.5. featureCounts v1.5.0-p3 was used to count the number of reads mapped to each gene. And then FPKM of each gene was calculated based on the length of the gene and reads count mapped to this gene. Differential expression analysis was performed using the DESeq2 R package (1.20.0). DESeq2 provides statistical routines for determining differential expression in digital gene expression data using a model based on the negative binomial distribution. The resulting *p*-values were adjusted using Benjamini and Hochberg’s approach for controlling the false discovery rate. The differentially expressed transcripts were selected using a statistical cut-off of adjusted *p*-value ≤ 0.05 and fold change (log2) > 1.0 and <−1.0. Fold enrichment analyses were performed using the DAVID bioinformatics tool (v6.8), and the criteria used to select significantly altered gene sets were Benjamini–Hochberg FDR of 5% and a *p* < 0.05. The complete fold enrichment analyses from DAVID are provided in Supplementary Data 2. While DAVID primarily employs a Fisher’s exact test for overrepresentation analysis, we also leveraged the platform’s additional Fold Enrichment option, which allows for further contextualization of pathway enrichment beyond *p*-value significance. An additional gene set enrichment analysis was performed on selected differentially expressed transcripts utilizing the Molecular Signatures Database, MSigDb (Supplementary Data 4).

### Gene expression (qPCR) analysis

RNA was extracted using Quick-RNA Miniprep Kit (Zymo Research) and converted to cDNA with SuperScript™ IV VILO™ Master Mix (ThermoFisher Scientific) according to the manufacturer’s instructions. cDNA was amplified using Power SYBR™ Green PCR Master Mix (Applied Biosystems) and respective primers that were purchased from Integrated DNA Technologies (sequences listed in Supplementary Data 6) on a 7500 Real Time PCR System (Applied Biosystems) according to the manufacturer’s instructions. Three to five independent biology replicates were conducted for each cell line, and the graphed data represent the mean ± SD of three technical replicates of one biological replicate. An unpaired, two-tailed *t*-tests were applied to compare control and experimental shRNA conditions.

### Western blot analysis

Total cell lysates were analyzed for the detection of all proteins. Total cell lysates were resolved by SDS-PAGE (4–12% Bis-Tris gels, ThermoFisher Scientific). Following protein transfer to a PVDF membrane using iBlot™ 2 (ThermoFisher Scientific), membranes were blocked with TBST + 5% nonfat milk for 1 h at room temperature and then incubated with primary antibodies (listed in Supplementary Data 7) overnight at 4 °C while rocking. Following primary antibody incubation, blots were washed and incubated with secondary antibodies (anti-rabbit-HRP, Cell Signaling Technology, Cat#: 7074 or anti-mouse-HRP, Cell Signaling Technology, Cat#: 7076) at RT for 1 h, and blots were developed with SuperSignal™ West Pico PLUS Chemiluminescent Substrate (ThermoFisher Scientific), Immobilon Crescendo Western HRP substrate (Millipore Sigma), or Immobilon Forte Western HRP substrate (Millipore Sigma), herby referred to as Pico, Crescendo, or Forte, respectively. Protein levels were quantified using Photoshop or ImageJ software.

### Metabolomic analysis

Metabolomic analyses were performed at the Proteomics and Metabolomics Facility at The Wistar Institute. NOMO-1 cells expressing DOX inducible recombinant lentiviruses co-expressing RFP and shRNAs were induced with DOX for 48 h for non-tracing, total steady-state polar metabolite analysis. For untargeted TPM cells, were lysed in 80% methanol spiked with 0.2 μM heavy amino acid mixture (Cambridge Isotopes, Cat#: MSK-A2). For U-^13^C-glucose tracing experiments in NOMO-1 and OCI-AML3, cells from each shRNA-condition were DOX induced for 32 h, followed by culture in glucose-free RPMI-1640 media supplemented with 11.1 mM uniformly labeled U-^13^C-glucose (Cambridge Isotope Laboratories Inc., D-Glucose-U-^13^C, 99%, CLM-1396) for 16 h to reach isotopic steady-state. For U-^13^C-glucose tracing experiments in MOLM-14, cells from each shRNA-condition were DOX induced for 16 h, followed by culture in glucose-free RPMI-1640 media supplemented with 11.1 mM uniformly labeled U-^13^C-glucose (Cambridge Isotope Laboratories Inc., D-Glucose-U-^13^C, 99%, CLM-1396) for 16 h to reach isotopic steady-state. For U-^13^C-glutamine (Cambridge Isotope Laboratories Inc., L-glutamine-U13C5, 99%, CLM-1822) tracing experiments in NOMO-1 and OCI-AML3, cells from each shRNA-condition were DOX induced for 44 h, followed by culture in glutamine-free RPMI-1640 media supplemented with 4.1 mM U-^13^C-glutamine. For U-^13^C-glutamine tracing experiments in MOLM-14, cells from each shRNA-condition were DOX induced for 28 h, followed by culture in glutamine-free RPMI-1640 media supplemented with 4.1 mM U-^13^C-glutamine. For U-^15^*N*-glutamine (Cambridge Isotope Laboratories Inc., L-Glutamine-15N2, 98%, NLM-1328-PK) tracing experiments in NOMO-1, cells from each shRNA-condition were DOX induced for 44 h, followed by culture in glutamine-free RPMI-1640 media supplemented with 4.1 mM U-^13^C-glutamine. Unlabeled controls were also used for each shRNA condition. Polar metabolites were extracted using an ice-cold extraction solution containing 80% MeOH, 20% water. Samples were analyzed by LC-MS/MS on a Q Exactive HF-X mass spectrometer with HESI II probe in-line with a ThermoScientific Vanquish LC System. Samples were analyzed in a pseudorandomized order. LC separation was performed under HILIC conditions using a ZIC-pHILIC column, 150 × 2.1 mm, 5 μm, maintained at 45 °C (EMD Millipore). Mobile phase A was 20 mM ammonium carbonate, 0.1% ammonium hydroxide, pH 9.2, and mobile phase B was acetonitrile. Analytical separation was performed at 0.2 ml/min flow rate using the following gradient: 0 min, 85% B; 2 min, 85% B; 17 min, 20% B; 17.1 min, 85% B; and 26 min, 85% B. Samples were analyzed by either Full MS scans with polarity switching (all samples) or Full MS/data-dependent MS/MS scans with separate acquisitions for positive and negative polarities (unlabeled samples in isotope tracing experiments; sample pool, QC, in non-tracing experiments). Relevant MS parameters include: sheath gas, 40; auxiliary gas, 10; sweep gas, 2; auxiliary gas heater temperature, 350 °C; spray voltage, 3.5/3.2 kV for positive/negative polarities; capillary temperature, 325 °C; S-lens RF, 40. Full MS scans were acquired using a scan range of 65 to 975 *m*/*z*; 120,000 resolution; automated gain control (AGC) target of 1E6; and maximum injection time (IT) of 100 ms. Data-dependent MS/MS was performed on the 10 most abundant ions; 15,000 resolutions; AGC target of 5E4, maximum IT of 50 ms, isolation width of 1.0 *m*/*z*, and stepped normalized collision energy of 20, 40, 60. Raw data were processed using Compound Discoverer 3.1 (ThermoFisher Scientific) with separate analyses for positive and negative polarities. Metabolites were identified by matching accurate mass and retention time to standards or querying MS/MS scans against the mzCloud spectral database (full match, score > 50; mzCloud.org). Identifications were transferred to labeled samples in isotope tracing experiments with consideration for all possible 13 C or 15 N isotopologues. Metabolite levels were quantified by integrated peak area using Full MS only data. Metabolite levels from non-tracing experiments were corrected for potential errors caused by instrument drift using peak areas from technical injections of the QC pool run at the beginning, middle, and end of the sample set. Isotope tracing data were corrected for natural 13 C or 15 N abundance and isotope tracer purity. Metabolite levels were further normalized to total protein recovered from the polar metabolite extraction pellet. The graphed data for all presented metabolomic analyses (steady-state and isotopologue tracing) represent the mean ± SD of three technical replicates of one biological replicate. An unpaired, two-tailed *t*-test was applied to compare the control with each experimental shRNA condition (shNT versus shATF4-1 or shNT versus shATF4-2).

### Amino acid rescue experiments

Modified media was prepped using RPMI-1640 Medium Modified without L-glutamine, amino acids, and glucose according to the manufacturer’s instructions (US Biological). The following modified media were made: RPMI-1640-based media supplemented with standard concentrations (1×), double the concentration (2×), or five times the standard concentration (5×) of affected amino acids (arginine, asparagine, aspartic acid, cysteine, glutamic acid, glutamine, glycine, proline, and serine) according to standard concentrations listed by the manufacturer (ThermoFisher Scientific). Cell viability was assessed daily utilizing the dye exclusion test with Trypan Blue Solution according to the manufacturer’s instructions (ThermoFisher Scientific). Three independent biology replicates were conducted for each cell line, and the graphed data represent the mean ± SD of three technical replicates of one biological replicate. An unpaired, two-tailed *t*-tests were applied to compare control and experimental shRNA conditions.

### Proteomics

Proteomic analyses were performed at The Einstein Proteomics Core at the Albert Einstein College of Medicine. For SILAC tracing experiments, NOMO-1 cells from each shRNA-condition were DOX induced for 12 h, followed by culture in lysine- and arginine-free RPMI-1640 media supplemented with 0.22 mM uniformly labeled U-^13^C-^15^N-lysine (ThermoFisher Scientific, L-Lysine-U13C6, U15N2, 88209) and 1.15 mM uniformly labeled U-^13^C-^15^N-arginine (ThermoFisher Scientific, L-Arginine-U13C6, U15N4, 89990) for 36 h to reach isotopic steady-state. For each replicate, proteins were extracted from 3 million cells using protein lysis buffer solution containing 6 M urea, 2 M thiourea, 50 mM ammonium bicarbonate, and 1× Halt Protease Inhibitor Cocktail (ThermoFisher Scientific, 78445) at pH 8. All protein samples were then digested with 5 mM dithiothreitol (DTT) for 1 h at room temperature and 20 mM iodoacetamide for 30 min in the dark, followed by sequencing grade trypsin (Promega, V5111) at an approximate concentration of 1:50 enzyme:sample (*w*:*w*) overnight at room temperature. Prior to mass spectrometry analysis, samples were desalted using a 96-well plate filter (Orochem) packed with 1 mg of Oasis HLB (Hydrophilic-Lipophilic-Balanced) C-18 resin (Waters). Briefly, the samples were resuspended in 100 µl of 0.1% trifluoroacetic acid (TFA) and loaded onto the HLB resin, which was previously equilibrated using 100 µl of the same buffer. After washing with 100 µl of 0.1% TFA, the samples were eluted with a buffer containing 70 µl of 60% acetonitrile and 0.1% TFA and then dried in a vacuum centrifuge. Samples were resuspended in 10 µl of 0.1% TFA and loaded onto a Dionex RSLC Ultimate 300 (ThermoFisher Scientific), coupled online with an Orbitrap Fusion Lumos (ThermoFisher Scientific). Chromatographic separation was performed with a two-column system, consisting of a C-18 trap cartridge (300 µm ID, 5 mm length) and a picofrit analytical column (75 µm ID, 25 cm length) packed in-house with reversed-phase Repro-Sil Pur C18-AQ 3 µm resin. Peptides were separated using a 120 min gradient from 4-30% buffer B (buffer A: 0.1% formic acid, buffer B: 80% acetonitrile + 0.1% formic acid) at a flow rate of 300 nl/min. The mass spectrometer was set to acquire spectra in a data-dependent acquisition (DDA) mode. Briefly, the full MS scan was set to 300–1200 *m*/*z* in the Orbitrap with a resolution of 120,000 (at 200 *m*/*z*) and an automatic gain control (AGC) target of 5 × 10e5. MS/MS was performed in the ion trap using the top speed mode (2 secs), an AGC target of 1 × 10e4, and an HCD (higher-energy C-trap dissociation) collision energy of 35. Proteome raw files were searched using MaxQuant (v2.0) using the SwissProt human database (updated June 2021)^58^. Search settings were kept as standard, with quantification multiplicity set as 2 (standard amino acids vs heavy labeled lysine and arginine using ^13^C and ^15^N). The search for the total proteome included variable modification of N-terminal acetylation, and fixed modification of carbamidomethyl cysteine. Trypsin was specified as the digestive enzyme with up to 2 missed cleavages allowed. Mass tolerance was set to 10 pm for precursor ions and 0.2 Da for products. The peptide and protein false discovery rate was set to 1%. Following the search, data were processed, and proteins were log2 transformed, normalized by the average value of each sample, and missing values were imputed using a normal distribution 2 standard deviations lower than the mean^59^. Statistical regulation was assessed using a heteroscedastic *t*-test (if *p*-value < 0.05). Data distribution was assumed to be normal, but this was not formally tested.

### DNA fiber assays

DOX-treated NOMO-1 or OCI-AML3 cells expressing shNT, shATF4-1, or shATF4-2 were incubated for 30 min with 50 µM CldU, washed with PBS, and subsequently incubated for 30 min with 250 µM IdU. Cells were washed with PBS, resuspended at 4000 cells/µl, and lysed on slides (Superfrost Plus microscope slides, Fisher Scientific) with lysis buffer containing 200 mM Tris–HCl, 50 mM ethylenediaminetetraacetic acid, 0.5% SDS, pH 7.4 buffer. Slides were tilted 60˚ to spread DNA fibers, air dried, fixed in 3:1 methanol:acetic acid for 10 min, and stored overnight at −20 °C. DNA fibers were denatured in 2.5 M HCl for 2.5 h, washed with PBS, and then blocked with 2% bovine serum albumin (BSA) in PBS for 40 min at RT. Slides were incubated with primary antibodies recognizing CldU (Abcam, catalog# ab6326, dilution 1:300) and IdU (BD Biosciences, catalog# 347580, dilution 1:100) for 2.5 h at RT, washed, and then incubated with AlexaFluor488 and AlexaFluor594 conjugated secondary antibodies (ThermoFisher Scientific, catalog# A11062 and A21470, dilutions 1:300) for 1 h at RT. Images were acquired with a Leica Stellaris 8 microscope, and fiber lengths were measured using ImageJ software. Three to five independent biology replicates were conducted for each cell line, and the graphed data represent the mean ± SD of three technical replicates of one biological replicate. An unpaired, two-tailed *t*-tests were applied to compare control and experimental shRNA conditions.

### Repli-seq analysis

Repli-seq assays were adapted from refs. ^48,49^. Briefly, DOX-treated NOMO-1 or OCI-AML3 cells expressing control or ATF4 targeting shRNAs were incubated for 2 h with 100 µM BrdU, washed with PBS, and fixed in 70% ethanol. Cells were then stained with FX cycle/RNase solution (ThermoFisher Scientific) and subjected to fluorescence active cell sorting to isolate the cell cycle fractions S1–S4 as shown in Fig. 7C and Supplementary Fig. 10A. DNA was extracted from each fraction, precipitated, and then sonicated to an average length between 200 and 300 bp using a Covaris S220. DNA was then incubated with anti-BrdU (B44, BD Pharmingen) overnight, followed by immunoprecipitation and Protein G Dynabeads^TM^ (ThermoFisher Scientific). Ten nanograms of eluted and cleaned DNA were amplified by PCR and then subjected to library preparation using the xGen^TM^ MethylSeq DNA library kit (Integrated DNA Technologies). Prepared libraries were sequenced using a NovaSeq X Plus targeting approximately 30 M 2 × 150 paired-end reads per condition. Bioinformatic analysis, including normalization, calculation of RTI from 4 S-phase fractions, and quantification of IZs, TSs, and CTRs were carried out^50^. Briefly, to evaluate differences in region lengths across experimental conditions, we extracted length distributions from BED files corresponding to CTRs (control), IZs, and TSs regions for each sample. Region lengths were visualized using violin plots grouped by sample within each region type.

### Ethics and inclusion statement

Local researchers have been included throughout the entire research process, including but not limited to study design, study implementation, data ownership, intellectual property, and authorship of publications. This research does not cause any stigmatization, incrimination, discrimination, or any other personal risk to participants. All research was conducted under the guidance and surveillance of the institutional biohazard committees of both Fox Chase Cancer Center and Washington University in Saint Louis to ensure researcher safety and minimize risk to researchers.

### Statistics and reproducibility

Graphpad Prism 11 and Microsoft Excel were used to perform statistical data analysis. Continuous outcome variables were compared using a two-sided, two-sample *t*-test (paired). For multiple comparisons, one-way ANOVA with Dunnett’s, Sidak’s multiple comparison test, or a two-stage linear Benjamini–Krieger–Yekutieli (BKY) procedure was used. No statistical method was used to predetermine sample size. No data were excluded from the analyses. Data collection was conducted blindly for Western blotting and qPCR analysis. Measurement for cell viability, flow cytometry, and microscopy, were performed by different individuals who were blinded to the experimental groups. Nucleotide sequencing (i.e., RNA-seq and Repli-seq) and mass spectrometry analysis (i.e., metabolomics and proteomics) samples were blinded prior to acquisition.

### Reporting summary

Further information on research design is available in the Nature Portfolio Reporting Summary linked to this article.

## Supplementary information

Supplementary Information

Description of Additional Supplementary Files

Supplementary Data 1

Supplementary Data 2

Supplementary Data 3

Supplementary Data 4

Supplementary Data 5

Supplementary Data 6

Supplementary Data 7

Reporting Summary

Transparent Peer Review file

## Source data

Source data

## Data Availability

The RNA-seq data and the Repli-seq data generated in this study have been deposited with links under the BioProject accession number PRJNA1462062. Both the processed RNA-seq and Repli-seq data generated in this study are also provided in the Source Data file. The Metabolomics data generated in this study have been deposited in the Metabolomics Workbench database under accession code PR002979. The processed metabolomics data generated in this study are provided in the Source Data file. The Proteomics data generated in this study have been deposited on the ProteomeXchange via the PRIDE database under accession code PXD074631. The processed proteomics data generated in this study are provided in the Source Data file. Source data are provided with this paper.
