# Supplementary material for: ATF4 coordinates amino acid and nucleotide synthesis with selective protein translation to ensure proper DNA replication timing in leukemia cells
Source: Nat Commun. 2026 Jun 20;17:7760. doi: 10.1038/s41467-026-74324-1 (PMC13434596; doi:10.1038/s41467-026-74324-1)
Supplement: Supplementary file 1 [file 41467_2026_74324_MOESM10_ESM.pdf]

nature portfolio

Corresponding author(s): Stephen Sykes
Last updated by author(s): 05/05/2026

# Reporting Summary

Nature Portfolio wishes to improve the reproducibility of the work that we publish. This form provides structure for consistency and transparency in reporting. For further information on Nature Portfolio policies, see our Editorial Policies and the Editorial Policy Checklist.

## Statistics

For all statistical analyses, confirm that the following items are present in the figure legend, table legend, main text, or Methods section.

| n/a | Confirmed | |
|---|---|---|
| ☐ | ☒ | The exact sample size (*n*) for each experimental group/condition, given as a discrete number and unit of measurement |
| ☐ | ☒ | A statement on whether measurements were taken from distinct samples or whether the same sample was measured repeatedly |
| ☐ | ☒ | The statistical test(s) used AND whether they are one- or two-sided<br>*Only common tests should be described solely by name; describe more complex techniques in the Methods section.* |
| ☒ | ☐ | A description of all covariates tested |
| ☐ | ☒ | A description of any assumptions or corrections, such as tests of normality and adjustment for multiple comparisons |
| ☐ | ☒ | A full description of the statistical parameters including central tendency (e.g. means) or other basic estimates (e.g. regression coefficient) AND variation (e.g. standard deviation) or associated estimates of uncertainty (e.g. confidence intervals) |
| ☐ | ☒ | For null hypothesis testing, the test statistic (e.g. *F*, *t*, *r*) with confidence intervals, effect sizes, degrees of freedom and *P* value noted<br>*Give P values as exact values whenever suitable.* |
| ☒ | ☐ | For Bayesian analysis, information on the choice of priors and Markov chain Monte Carlo settings |
| ☒ | ☐ | For hierarchical and complex designs, identification of the appropriate level for tests and full reporting of outcomes |
| ☒ | ☐ | Estimates of effect sizes (e.g. Cohen's *d*, Pearson's *r*), indicating how they were calculated |

*Our web collection on statistics for biologists contains articles on many of the points above.*

## Software and code

Policy information about availability of computer code

Data collection

For flow cytometry, either FACSDiva or CytExpert were used for collection.

BioRad Image Lab Software or ImageJ was used to image western blot data.

Applied Biosystems 7500 Software v2.x was used in the collection of qPCR data.

Illumina based HiSeq Control Software (HCS) was used in the sequencing of RNA and DNA (i.e. Repli-seq)

Thermo Scientific™ Xcalibur was used in the acquisition of metabolomic and proteomic datasets.

Leica Application Suite X software was used in the acquisition of DNA fibre images.

Data analysis

For flow cytometry, either Flowjo or CytExpert was used for analysis.

BioRad Image Lab Software or ImageJ was used to quantify western blot data.

RNA-seq Differential expression analysis was performed using the DESeq2 R package (1.20.0) followed by enrichment analyses using either open source DAVID, MigDB and Enrichr.

The source code used to analyze Repli-seq data has been published: 50) Ji F, Van Rechem C, Whetstine JR, Sadreyev RI. Computational workflow for integrative analyses of DNA replication timing, epigenomic, and transcriptomic data. STAR Protoc. 2022 Nov 8;3(4):101827. doi: 10.1016/j.xpro.2022.101827. PMID: 36386876; PMCID: PMC9647704.

Proteome raw files were searched using MaxQuant (v2.0) using the SwissProt human database.

Graphing and statistical analyses were carried out using either Graphpad Prism 11 or Microsoft Excel.

For manuscripts utilizing custom algorithms or software that are central to the research but not yet described in published literature, software must be made available to editors and reviewers. We strongly encourage code deposition in a community repository (e.g. GitHub). See the Nature Portfolio guidelines for submitting code & software for further information.

## Data

Policy information about availability of data

All manuscripts must include a data availability statement. This statement should provide the following information, where applicable:

- Accession codes, unique identifiers, or web links for publicly available datasets
- A description of any restrictions on data availability
- For clinical datasets or third party data, please ensure that the statement adheres to our policy

For expression data of ATF4 in AML, the open source search databases Bloodspot.edu and Vizome.org was used.

The RNA-seq and Repli-seq data have been deposited in Sequence Read Archive (SRA) database of the National Center for Biotechnology Information (NCBI) under one accession code, PRJNA1462062.

The Metabolomics data generated in this study have been deposited in the Metabolomics Workbench database under accession code PR002979.

The Proteomics data generated in this study have been deposited in the ProteomeXchange via the PRIDE database under accession code PXD074631.

## Research involving human participants, their data, or biological material

Policy information about studies with human participants or human data. See also policy information about sex, gender (identity/presentation), and sexual orientation and race, ethnicity and racism.

| | |
|---|---|
| Reporting on sex and gender | Due to the limited availability of PD-AML samples, samples were selected based on availability and genetic subtype. Sex and gender based assays were not considered because the samples sizes of samples used were not large enough to draw meaningful conclusions. Furthermore, no animal studies were performed in this study. |
| Reporting on race, ethnicity, or other socially relevant groupings | Due to the limited availability of PD-AML samples, samples were selected based on availability and genetic subtype. Race and ethnicity were not considered because the samples sizes of samples used were not large enough to draw meaningful conclusions. |
| Population characteristics | See above |
| Recruitment | Patient samples were recovered from a biobank, where all patients are offered to provide sample under informed consent. Only those patients that volunteered samples and gave consent were collected. |
| Ethics oversight | The de-identified patient-derived AML sample studies were approved by the Institutional Review Board (IRB) of Fox Chase Cancer Center, under the IRB protocol 16-9037. All human studies were conducted in accordance with the "Declaration of Helsinki" principles. |

Note that full information on the approval of the study protocol must also be provided in the manuscript.

# Field-specific reporting

Please select the one below that is the best fit for your research. If you are not sure, read the appropriate sections before making your selection.

☒ Life sciences ☐ Behavioural & social sciences ☐ Ecological, evolutionary & environmental sciences

For a reference copy of the document with all sections, see nature.com/documents/nr-reporting-summary-flat.pdf

# Life sciences study design

All studies must disclose on these points even when the disclosure is negative.

| | |
|---|---|
| Sample size | Given that AML is comprised of numerous distinct genetic subtypes we used multiple AML cell lines and patient-derived AML samples to ensure that our observations were not cell line specific or restricted to a single genetic subtype.<br><br>No statistical method was used to predetermine sample size because the study was discovery-based and therefore the effect size was not |

| | |
|---|---|
| | known prior to the study. Therefore, sample size was based on industry standards for discovery, which apply 3-5 replicates per biological experiment and then conducting multiple biological replicates to confirm reproducibility. |
| Data exclusions | No data exclusions were made |
| Replication | Three-Five Biological replicates per experiment and each biological contained 3-5 technical replicates. All attempts were successful and showed comparable results. |
| Randomization | AML is comprised of numerous distinct genetic subtypes and sample were randomized based on genetic subtype |
| Blinding | For metabolomics, repli-seq, proteomics and RNA-seq were coded by the researchers, however, the samples were blinded to those conducting sample acquisition. Data collection was conducted blindly for western blotting and qPCR analysis. Measurement for cell viability, flow cytometry, microscopy, were performed by different individuals who were blinded to the experimental groups. |

# Reporting for specific materials, systems and methods

We require information from authors about some types of materials, experimental systems and methods used in many studies. Here, indicate whether each material, system or method listed is relevant to your study. If you are not sure if a list item applies to your research, read the appropriate section before selecting a response.

## Materials & experimental systems

| n/a | Involved in the study |
|---|---|
| ☐ | ☒ Antibodies |
| ☐ | ☒ Eukaryotic cell lines |
| ☒ | ☐ Palaeontology and archaeology |
| ☒ | ☐ Animals and other organisms |
| ☐ | ☒ Clinical data |
| ☒ | ☐ Dual use research of concern |
| ☒ | ☐ Plants |

## Methods

| n/a | Involved in the study |
|---|---|
| ☒ | ☐ ChIP-seq |
| ☐ | ☒ Flow cytometry |
| ☒ | ☐ MRI-based neuroimaging |

## Antibodies

| | |
|---|---|
| Antibodies used | The following antibodies were used: anti-ATF4 (Cell Signaling Technology, D4B8, Cat#: 11815, Lot#: 5) with the dilution 1:1000 and developed with Forte, anti-ASNS (Cell Signaling Technology, D4O4B, Cat#: 70720, Lot#: 1) with the dilution 1:1000 and developed with Forte, anti-ASS1 (Cell Signaling Technology, D4O4B, Cat#: 70720, Lot#: 1) with the dilution 1:2000 and developed with Pico, anti-BCAT1 (Cell Signaling Technology, D6D4K, Cat#: 88785, Lot#: 1) with the dilution 1:1000 and developed with Forte, anti-CBS (Cell Signaling Technology, D8F2P, Cat#: 14782, Lot#: 1) with the dilution 1:1000 and developed with Forte, anti-CTH (Cell Signaling Technology, D1N1D, Cat#: 19689, Lot#: 1) with the dilution 1:1000 and developed with Crescendo, anti-GLDC (Cell Signaling Technology, Cat#: 12794, Lot#: 1) with the dilution 1:1000 and developed with Forte, anti-GOT1 (Cell Signaling Technology, E4A4O, Cat#: 34423, Lot#: 1) with the dilution 1:1000 and developed with Crescendo, anti-GPT2 (Proteintech, Cat#: 16757-1-AP, Lot#: 00061201) with the dilution 1:1000 and developed with Crescendo, anti-IDH1 (Cell Signaling Technology, D2H1 Cat#: 8137, Lot#: 5) with the dilution 1:1000 and developed with Crescendo, anti-PHGDH (Cell Signaling Technology, Cat#: 13428, Lot#: 1) with the dilution 1:1000 and developed with Forte, anti-PSAT1 (Novus, Cat#: NBP1–32920, Lot#: 42242) with the dilution 1:1000 and developed with Crescendo, anti-PYCR1 (Cell Signaling Technology, E7P3I, Cat#: 37635, Lot#: 1) with the dilution 1:1000 and developed with Forte, anti-SHMT2 (Cell Signaling Technology, Cat#: 12762, Lot#: 1) with the dilution 1:1000 and developed with Crescendo, and anti-α-tubulin (Sigma-Aldrich, DM1A, Cat#: T9026, Lot#: 083M4847V) with the dilution 1:4000 developed with Pico. Secondary antibodies used included anti-rabbit-HRP (Cell Signaling Technology, Cat#: 7074, Lot#: 28) and anti-mouse-HRP (Cell Signaling Technology, Cat#: 7076, Lot#: 36) with the dilution 1:4000 for all proteins except for ATF4, BCAT1, CBS, GOT1, and PHGDH which was used at 1:2000. Additional antibodies used are: 1) Actin (Cell Signaling Technology, Cat#: 8457, Clone D6A8); 2) MTHFD1L (Cell Signaling Technology, Cat#: 14998, Clone D7O8E); 3) MTHFD2 (Invitrogen, Cat#: MA5-51284, Clone3C9Q8); 4) PSPH (Cell Signaling Technology, Cat#: 58258, Clone E1D2X); 5) ALDH1L2 (ProteinTech, Cat#: 21391-1-AP); and 6) SHMT1 (ProteinTech, Cat#: 30192-1-AP). |
| Validation | All antibodies used had been previously validated and is listed on the respective manufacturer's websites. |

## Eukaryotic cell lines

Policy information about cell lines and Sex and Gender in Research

| | |
|---|---|
| Cell line source(s) | The following human AML cell lines were obtained from the German Collection of Microorganism and Cell Cultures (DMSZ), catalogue numbers in parenthesis: NOMO-1 (ACC 542), OCI-AML3 (ACC 582), OCI-AML2 (ACC 99), and MOLM-14 (ACC 777). The following human AML cell lines were obtained from the American Type Culture Collection (ATCC), catalogue numbers in parenthesis:, K-562 (CCL-243) and MV4-11 (CRL-9591) |
| Authentication | Cell line authentication was carried out by IDEXX using the short tandem repeat (STAR) profiling method. |
| Mycoplasma contamination | All cells are routinely tested for mycoplasma every three months and have tested negative for mycoplasma on each occasion. |

| | |
|---|---|
| Commonly misidentified lines (See ICLAC register) | None of the cell lines used in this study are listed on the https://iclac.org/databases/cross-contaminations/ |

## Clinical data

Policy information about clinical studies

All manuscripts should comply with the ICMJE guidelines for publication of clinical research and a completed CONSORT checklist must be included with all submissions.

| | |
|---|---|
| Clinical trial registration | Not applicable. |
| Study protocol | Not applicable |
| Data collection | Not applicable |
| Outcomes | Not applicable |

## Plants

| | |
|---|---|
| Seed stocks | Not applicable |
| Novel plant genotypes | Not applicable |
| Authentication | Not applicable |

## Flow Cytometry

### Plots

Confirm that:

☒ The axis labels state the marker and fluorochrome used (e.g. CD4-FITC).

☒ The axis scales are clearly visible. Include numbers along axes only for bottom left plot of group (a 'group' is an analysis of identical markers).

☒ All plots are contour plots with outliers or pseudocolor plots.

☒ A numerical value for number of cells or percentage (with statistics) is provided.

### Methodology

| | |
|---|---|
| Sample preparation | Cells were washed three times prior to antibody or dye staining. Cells were then washed and additional three times prior to running on the cytometer |
| Instrument | BD lSR II or BD Cytoflex |
| Software | FlowJo |
| Cell population abundance | At least 10,000 events were collected for each analysis. Sorted population were run immediately after sorting to confirm 95% + purity |
| Gating strategy | We used FSC/SSC was first used to separate cells from debris. We then applied a forward scatter discriminator (FSC-H versus FSC-A to eliminate doublets. Cell viability stains such as propidium iodide or annexin V were used to distinguish dead and live cell populations. |

☒ Tick this box to confirm that a figure exemplifying the gating strategy is provided in the Supplementary Information.
