# Supplementary material for: ATF4 coordinates amino acid and nucleotide synthesis with selective protein translation to ensure proper DNA replication timing in leukemia cells
Source: Nat Commun. 2026 Jun 20;17:7760. doi: 10.1038/s41467-026-74324-1 (PMC13434596; doi:10.1038/s41467-026-74324-1)
Supplement: Supplementary file 2 [file 41467_2026_74324_MOESM11_ESM.pdf]

nature portfolio

Peer Review File

ATF4 coordinates amino acid and nucleotide synthesis with selective protein translation to ensure proper DNA replication timing in leukemia cells

Corresponding Author: Professor Stephen Sykes

This file contains all reviewer reports in order by version, followed by all author rebuttals in order by version.

Version 0:

Reviewer comments:

Reviewer #1

(Remarks to the Author)
This paper describes how loss of ATF4 impairs amino acid synthesis and metabolism and tRNA charging, leading to a defect at the G1/S transition in tumor cells. The results are supportive of the existing literature on the role of ATF4 in the proliferation of various cancer cell types.

Reviewer #2

(Remarks to the Author)
Huhn and colleagues conducted a significant study elucidating the role of ATF4 in coordinating the synthesis of amino acids and nucleotides, with a specific focus on the synthesis of proteins crucial for DNA replication and the G1/S transition. By knocking down ATF4, the authors observed a cell cycle arrest in G1, as confirmed by BrdU staining. They employed a transcriptomic approach to demonstrate that ATF4 maintains the basal transcription of enzymes that catalyze the biosynthesis and tRNA charging of various amino acids. Additionally, through comprehensive analyses involving steady-state metabolomics and stable isotope tracing experiments, the authors found that ATF4 supports the synthesis of several amino acids derived from glucose metabolism, glutamine anaplerosis, and de novo purine and pyrimidine synthesis. These results were further supported by proteomic labeling studies, which showed that inhibiting ATF4 selectively impairs the synthesis of proteins essential for DNA replication.

General comments:
It is important to note that while these findings align with previous studies that have individually reported the involvement of ATF4 in regulating tRNA metabolism, serine/glycine synthesis, and nucleotide synthesis, this study stands out due to its ability to consolidate these distinct findings into a comprehensive framework specifically in the context of acute myeloid leukemia.

1) The regulation of tRNA aminoacylation by ATF4 is well established. To add some novelty to this part, it will be essential to show that ATF4 loss can affect tRNA charging in these AML cells.

2) It is possible that ATF4 loss affects the pentose phosphate pathway; authors should present the levels of 6-phosphogluconate, ribose 5-phosphate, and PRPP derived from 13C-glucose labeling.

3) The most novel aspect of this study is the data presented in Figure 7. However, the authors fail to draw significant conclusions from it. Based on the reactome pathway and WIKIPathways analysis, DNA unwinding and replication appear to be the most significant processes regulated by ATF4. It would be beneficial for the authors to delve deeper into these findings and explore the downstream processes of DNA replication regulated by ATF4. Conducting Repli-seq analysis to measure the effects of ATF4 loss or overexpression on nascent DNA replication strands would be particularly interesting.

4) The authors should assess the impact of ATF4 on cell cycle activity at the molecular level, including evaluating pRB (retinoblastoma protein) and ETF4 (E2F transcription factor 4) activity.

Specific comments:
Figure 3D: It is observed that the knockdown of ATF4 mediated by shRNA#2 led to an increase in glycine levels, while shRNA ATF4-#1 reduced glycine levels. The authors should provide an explanation for this discrepancy.

Figure 3E: It would be valuable to present the protein abundance of PSPH, SHMT1, MTHFD1L, MTHFD2, and ALDH1L2 to support their findings further.

Reviewer #3

(Remarks to the Author)
Summary
The authors report on how transcriptional regulators are involved in regulation of cell-cycle dependent changes in protein translation and cellular metabolism, which has not been well studied so far and is novel. Exploiting the metabolism of cancer cells therapeutically is in general a particularly interesting and important field to study.
It is known from the literature that AT4 expression is increased in leukemia-initiating cells and that inhibition of AT4 leads to decreased disease progression in mouse models of AML harboring KMT2A rearrangements. Furthermore, in AML, data suggests a role of ATF4 in regulating the integrated stress response (ISR), a conserved mechanisms activated upon for instance unfold-protein or mitochondrial stress to halt global protein expression in order to restore cellular homeostasis (https://www.science.org/doi/full/10.1126/scitranslmed.aax2863).

The authors report that ATF4 regulates metabolic processes and protein synthesis to drive the cell cycle transition from the G1 to S-phase.

Minor comments:

Figure 1

I would suggest being more specific in the text as highlighted in bold: "Next, a doxycycline-inducible shRNA system was utilized to determine the kinetics of anti-leukemia cell growth mediated by genetic ATF4 inhibition."

As the authors make a statement about the impact of AT4 pertubation playing a particular important role in KMT2A-, as well as Normal Karyotype leukemias, it would be interesting to see whether the effects observed for NOMO-1 (MLL-AF9) and MV4-11 (MLL-AF4), as well as OCI-AML3 (NPM1 mutated showing a similar gene expression signature as the MLLr acute leukemias) are not evident in acute leukemia cell lines without KMT2A or NPM1 genetic changes.

The authors are validating two different types (inducible and non-inducible) of shRNA mediated genetic knockdown, and do get sufficient gene knockdown on protein level. Do these shRNAs also leads to a downregulation of AT4 on mRNA expression level by qPCR? Is there a reason why the authors only focused on shRNA mediated knockdown and no sgRNA mediated CRISPR/Cas9 mediated knockout was performed, leading to potentially less off-target effects? Please advise.

The authors mention that ATF4 inhibition hinders cell cycle progression. Is there a particular reason they have chosen not to employ well-established FACS analyses like nuclear DNA content distribution? Such analyses could help in assessing the relative distribution of cell cycle phases between shNT and shATF4

Figure label 1H reads "MV4-11 single cell clone from eeach"

Figure 2

To identify downstream pathways that play a role in ATF4 mediated leukemia the authors performed RNAseq analyses for each shRNA condition in NOMO-1 cells, the timepoint chosen was 24 hours, which should be sufficiently long for showing transcriptional changes without being too late as they would have to deal with regulatory feedback signaling effects. They chose a standard statistical cut-off and fold-chance (log2) > 1 and < -1.0. Next they performed pathway enrichment analysis using DAVID. It would be interesting and making the case stronger if using another gene set enrichment analysis platform, such as MSigDb will come up with the same enriched pathways. To statistically validate significance of the observed they performed Benjamini-Hochberg analysis, which is a common analysis performed in these kind of experiments.

The authors report that the three themes of the enriched pathways were amino acid metabolism, tRNA aminoacylation/charging and UPR signaling. It would also be of importance whether knockdown of ATF4 is also interfering with the MLL gene expression program (such as Hoxa9 and Meis1) leading to downregulation of previously published gene expression signatures (e.g. Günther et al. 2008 Genes and Dev). This would also shed light on the question whether observed effects on cell cycle and survival by knockdown of ATF4 is able to interfere with the MLL induced transcriptional program.

Various metabolic pathways were decreased upon ATF4 knockdown, especially enzymes that play a role in amino acid biosynthesis. The authors also mention a downregulation of multiple amino acid transporters e.g. SLC1A5, some of these reporters have been identified as potential targets to reduce cancer cell growth also in other solid tumors.

As metabolic pathways are important for growth of cancer cells in general it would also be of interest for the readership to

know that any of these genes (enzymes) or AA reporters account as common essential genes when you e.g. query the various cancer cell lines in the publicly available depmap dataset or are specific hits. Can you also narrow down on specific metabolic pathways where multiple of the downregulated AA transporters are involved?

Page 6/55 merged manuscript, please correct typo in sentence: “In addition to amino acid biosynthesis enzymes, RNA-seq data showed that, compared to shNT controls, NOMO-1 cells expressing ATF4-targteing shRNAs displayed lower levels of multiple amino acid transporters”

Figure 2 appears somewhat redundant. In my opinion, it might be more effective to either display only B or C, relocating the other to the supplementary material, or perhaps create a combined plot (since most of the terms are identical, you could just stack the shATF4-1 and shATF4-2 scores). Additionally, the qPCR validations in sections D and E might be better suited for the supplementary material, as they don't seem to offer much new insight. The enrichment analyses already make it quite evident that metabolic pathways are the primary terms in this context.

It would be great if authors could add some details into the RNA-seq methods section such as RIN cutoff used, Q20, Q30 percentage, the reference genome used, the design used within DESeq2 for the differential expression analysis.

I find myself somewhat unclear on the type of analysis employed to arrive at the enrichment results. To my understanding, DAVID typically utilizes a version of a Fisher-test for overrepresentation analysis, contrasting the fraction of genes from a given set (like GO BP sets) in the significantly up- or down-regulated genes against those in the background (or non-significant genes). This leads to an adjusted p-value and a set size for each set but doesn't provide an enrichment score. On the other hand, gene-set enrichment analysis is a rank-based method that accounts for all genes, not only those marked as significantly up- or down-regulated. This ranking, often by adjusted p-value, leads to something akin to a 'normalized enrichment score' (as described in https://www.pnas.org/doi/full/10.1073/pnas.0506580102). While it’s probable that the results wouldn’t vary much, some clarification in the methods section would be appreciated.

ATF4 inhibition affects selective protein translation

The quantification of 3000 proteins seems somewhat low, considering that in our experience SILAC-based proteomics in cell lines often yields results in the range of 6000 proteins. While this discrepancy might not directly affect the results presented in this study, it caught our attention. Perhaps the authors could provide some commentary or explanation for this.

Version 1:

Reviewer comments:

Reviewer #2

(Remarks to the Author)
The authors have addressed most of my previous comments; however, one issue remains unresolved. The manuscript states that ATF4 knockdown does not affect the protein levels of PSPH, MTHFD1L, MTHFD2, ALDH1L2, or SHMT1, despite apparent transcriptional downregulation. This finding appears inconsistent with multiple prior studies (e.g., PMID: 33646118, PMID: 34303878, PMID: 36051358), which have demonstrated that ATF4 is a key upstream regulator of enzymes involved in serine biosynthesis and the THF cycle. In particular, MTHFD2 protein levels are significantly reduced upon ATF4 depletion in various cellular contexts.
Given this strong precedent, the absence of protein-level changes reported here is unexpected and raises concerns about the sensitivity or execution of the western blot analysis. If the authors intend to retain this conclusion, they should include the supporting western blot data rather than stating “data not shown,” to allow readers to evaluate the findings directly. Alternatively, if the data are inconclusive or of insufficient quality, the authors should consider revising or removing the statement to avoid contradicting well-established literature.

Reviewer #3

(Remarks to the Author)
Huhn et al. identified the transcriptional regulator ATF4 as an important player in generating nucleotides and protein machinery needed for initiation and timing of DNA replication in acute leukemia. Characterization and understanding of deregulated metabolic pathways have previously been identified as a vulnerability in acute leukemia that can be exploited for leukemia therapy (e.g. IDH inhibition in AML, PEG-Asparaginase in B-ALL). This study focusses on an important and emerging topic that could play a role in understanding the mode of action of several drugs that target leukemia metabolism and suggests ATF4 inhibition as a potential novel therapeutic option.

Specific Comments:

The authors show in Figure 1 that by using a doxycycline-inducible shRNA approach they achieve a complete genetic knockout (at least in NOMO-1) on protein level. Furthermore, they observe a cell cycle arrest in G1, as well as apoptosis. In line with previous reviewer’s comments I am wondering why the authors have not additionally employed a CRISPR/Cas9 approach and whether ATF4 is also showing a reduced expression on transcript level? The authors have not fully answered this question in the rebuttal letter. Please advise.

Did the authors observe a CD11b shift and cytological changes as shown in Supplementary Fig. 1 in other cell lines as well or were experiments only performed in one cell line?
In Figure 2 the authors nicely show in a GSEA analysis of RNAseq data after shRNA mediated ATF4 knockdown that transcripts genes fell into three major categories: amino acid metabolism, tRNA aminoacylation/charging, and UPR signaling. The following experiments show that ATF4 inhibition led to a downregulation of genes involved in various pathways, e.g. one-carbon metabolism, glutamate and aspartate metabolism.
It would be helpful, assessing the impact of the individual genes, if the authors could add a column with the DepMap Score to the analyzed dependency data in Supplementary Fig. 3
What method was used for the analysis you show in Figure 3 C-V. It is not mentioned in the text nor the Figure legend. It looks like all of these low-throuput validation experiments were performed in one cell line, NOMO-1? Did you assess the impact on these metabolites in other cell lines as well to rule out that we are looking at a cell-line specific effect?
In several paragraphs the results are purely displayed in a descriptic manner. E.g. starting from line 326. It would be important that the authors could interpret their observed results in order to draw more definite conclusions.
In Figure 6 the authors mention that downregulation of ATF4 by shRNA leads to a decreased mRNA expression of AIMP2 and EEF1E1, two proteins involved in the formation of the aminoacyl-tRNA synthetase complex. It would be interesting to find out whether formation of the tRNA synthetase complex is impacted by genetic inhibiton of ATF4. As the authors have accessibility to MS, they could prove this by performing a MS-Immunoprecipitation of the complex in control and ATF4 knockdown cells to investigate whether the composition of the complex is altered after ATF4 knockdown.
Figure 7
Indeed, detection of 2,918 proteins is a low end result in comparison to our experiences with MS proteomics analyses. However, the authors have addressed this and given a possible explanation for this in their rebuttal letter.

Version 2:

Reviewer comments:

Reviewer #2

(Remarks to the Author)
The authors have addressed my comments in the revision. Congratulations on an excellent and compelling body of work, carefully executed and clearly presented.

Reviewer #3

(Remarks to the Author)
All of my remarks have been adequately addressed.

Open Access This Peer Review File is licensed under a Creative Commons Attribution 4.0 International License, which permits use, sharing, adaptation, distribution and reproduction in any medium or format, as long as you give appropriate credit to the original author(s) and the source, provide a link to the Creative Commons license, and indicate if changes were

made.
In cases where reviewers are anonymous, credit should be given to 'Anonymous Referee' and the source.
The images or other third party material in this Peer Review File are included in the article's Creative Commons license, unless indicated otherwise in a credit line to the material. If material is not included in the article's Creative Commons license and your intended use is not permitted by statutory regulation or exceeds the permitted use, you will need to obtain permission directly from the copyright holder.
To view a copy of this license, visit https://creativecommons.org/licenses/by/4.0/

Response to Reviewers: NCOMMS-23-18711-T

We thank the reviewers for their appreciation of our work and are grateful for their constructive and insightful comments. We have addressed each of the reviewer's concerns and critiques in the following point-by-point rebuttal. These suggestions have been very helpful and, in our opinion, have resulted in a much-strengthened study. Original comments are in Times New Roman blue font and responses are in Arial black font.

REVIEWER COMMENTS

Reviewer #1 (Remarks to the Author):

This paper describes how loss of ATF4 impairs amino acid synthesis and metabolism and tRNA charging, leading to a defect at the G1/S transition in tumor cells. The results are supportive of the existing literature on the role of ATF4 in the proliferation of various cancer cell types.

ANSWER: We appreciate the Reviewer's recognition that our data are consistent with existing studies, underscoring the rigor of our analyses. In addition, our study provides new insights into the role of ATF4 in cancer biology. First, our analyses examined how ATF4 contributes to leukemia biology under basal/nutrient replete conditions, whereas the vast majority of existing data pertains to the role of ATF4 under stress conditions. Second, while ATF4 has been implicated in the regulation of protein synthesis as a transcriptional activator of tRNA synthetases, very few studies have examined the impact of disrupting ATF4 expression on protein synthesis. The few studies that have done so, have suggested that ATF4 globally regulates protein synthesis. However, these studies utilized assays that rely on the incorporation of methionine/cysteine, of which the cognate tRNA synthetases are regulated by ATF4 and thus could lead to an inadvertent over-estimation of the impact of ATF4 on translation. Using SILAC with heavy arginine and lysine, the biology of which is not influenced by ATF4, we discovered that ATF4 (under nutrient replete conditions) controls a small subset of proteins specifically related to DNA replication initiation. Third, we show that, through selective regulation of amino acid, nucleotide and protein synthesis, ATF4 promotes proper origin firing and timing of DNA replication. Finally, for the first time, our comprehensive study precisely shows how ATF4 coordinates multiple anabolic processes to support cellular duplication and the differentiation blockade of leukemia cells.

Reviewer #2 (Remarks to the Author):

Huhn and colleagues conducted a significant study elucidating the role of ATF4 in coordinating the synthesis of amino acids and nucleotides, with a specific focus on the synthesis of proteins crucial for DNA replication and the G1/S transition. By knocking down ATF4, the authors observed a cell cycle arrest in G1, as confirmed by BrdU staining. They employed a transcriptomic approach to demonstrate that ATF4 maintains the basal transcription of enzymes that catalyze the biosynthesis and tRNA charging of various amino acids. Additionally, through comprehensive analyses involving steady-state metabolomics and stable isotope tracing experiments, the authors found that ATF4 supports the synthesis of several amino acids derived from glucose metabolism, glutamine anaplerosis, and de novo purine and pyrimidine synthesis. These results were further supported by proteomic labeling studies, which showed that inhibiting ATF4 selectively impairs the synthesis of proteins essential for DNA replication.

General comments: It is important to note that while these findings align with previous studies that have individually reported the involvement of ATF4 in regulating tRNA metabolism, serine/glycine synthesis, and nucleotide synthesis, this study stands out due to its ability to consolidate these distinct findings into a comprehensive framework specifically in the context of acute myeloid leukemia.

1) The regulation of tRNA aminoacylation by ATF4 is well established. To add some novelty to this part, it will be essential to show that ATF4 loss can affect tRNA charging in these AML cells.

ANSWER: We agree that confirming that ATF4 inhibition results in a reduction of specific tRNA charging is a key point of interest. We attempted to establish collaborations with multiple experts in the tRNA field, however, we were unable to do so. Therefore, we devoted our efforts to addressing the more novel aspects of our study, specifically how ATF4 influences DNA replication.

2) It is possible that ATF4 loss affects the pentose phosphate pathway; authors should present the levels of 6-phosphogluconate, ribose 5-phosphate, and PRPP derived from 13C-glucose labeling.

ANSWER: PRPP did not show any difference in labeling between experimental conditions (Supplementary Figure **5E**). We were unable to detect 6-phosphogluconate or ribose-5-phosphate in the polar metabolites from our ^{13}C-glucose labeling experiments. However, we were able to detect another Pentose Phosphate Pathway intermediate, sedoheptulose-7-phosphate, which did not show any significant difference in ^{13}C-glucose labeling between experimental conditions (Supplementary Figure **5D**).

3) The most novel aspect of this study is the data presented in Figure 7. However, the authors fail to draw significant conclusions from it. Based on the reactome pathway and WIKIPathways analysis, DNA unwinding and replication appear to be the most significant processes regulated by ATF4. It would be beneficial for the authors to delve deeper into these findings and explore the downstream processes of DNA replication regulated by ATF4. Conducting Repli-seq analysis to measure the effects of ATF4 loss or overexpression on nascent DNA replication strands would be particularly interesting.

ANSWER: We agree with the reviewer that the potential regulatory role of ATF4 in the initiation of DNA replication is the most novel and exciting aspect of our study. We therefore took the Reviewer's advice to pursue this avenue further by performing a combination of DNA fibre assays and Repli-seq analysis. Employing DNA fibre assays as we had previously shown (PMID: 34081901), we observed that ATF4 inhibition reduces the speed of DNA replication as leukemia cells with ATF4 knockdown displayed significantly shorter DNA fibers compared to control cells (Figure **7A & 7B**). As recommended by the reviewer, we also carried out Repli-seq assays to evaluate the impact of ATF4 disruption on nascent DNA replication initiation and timing. This analysis revealed that ATF4 inhibition resulted in significant alterations in replication origin firing and timing. We found evidence that ATF4 inhibition can lead to both advances and delays in replication timing (RT) depending on the region of replication (Supplementary Figure **10C**). Overall, we found that inhibition of ATF4 altered the RT across a quarter of the genome in NOMO-1 cells and about 18% of OCI-AML3 cells. In both cell lines, approximately 60% were delays (rather than advancement) in RT. The most profound impact of ATF4 inhibition on RT was observed at regions of late DNA replication, particularly at the X chromosome and chromosomes 7, 10, 12, 14 and 20 (Fig. **7D** and Supplementary Fig. **10B, 12C, 13B, 13D, 14B** and **15D**). Visually examining the genomic tracks in IGV (Figure **7H** and Supplementary Figures **10G, 11-16**), we also noticed that the width of DNA replication initiation zones tended to be shorter in cells expressing ATF4 shRNAs compared to controls. Therefore, we employed a bioinformatic pipeline adapted from PMID: 36386876, to calculate several DNA replication attributes including the number and length of initiation zones (IZs), termination sites (TSs) and constant timing regions (CTRs). While we did not observe a substantial difference in the number of IZs, TSs and CTRs between control and experimental shRNA conditions, we did find that the length of IZs, TSs and CTRs were shorter in leukemia cells expressing ATF4-targeting shRNAs compared to controls (Figure **7E-G** and Supplementary Figure **10D-F**). These data highlight that through transcriptional

regulation of anabolic enzymes and tRNA synthetases, ATF4 maintains nucleotide pools and promotes the translation of DNA replication machinery to ensure proper the proper DNA replication firing and timing of leukemia cells. We are deeply thankful to the reviewer for this suggestion as we feel that these analyses have greatly strengthened this manuscript.

4) The authors should assess the impact of ATF4 on cell cycle activity at the molecular level, including evaluating pRB (retinoblastoma protein) and ETF4 (E2F transcription factor 4) activity.

ANSWER: We did assess the steady state levels of pRB-S780 and total-RB as well as ETF4 in cells where ATF4 has been knocked down, however, we did not observe any differences in either species (See Adjacent Figure). We also tested antibodies targeting pRB-S608 and pRB-S807/811, however, we could not detect a reliable signal for these antibodies even in control cells. It is possible that RB phosphorylation outside of S780 is affected ATF4 inhibition. However, since DNA damage and replication fork stalling can promote cell cycle arrest without alterations in Rb phosphorylation, it could also be possible that ATF4 inhibition could promote cell cycle arrest without affecting Rb phosphorylation. Yet, because we are unable to make a definitive conclusion, we omitted these results from the revised manuscript.

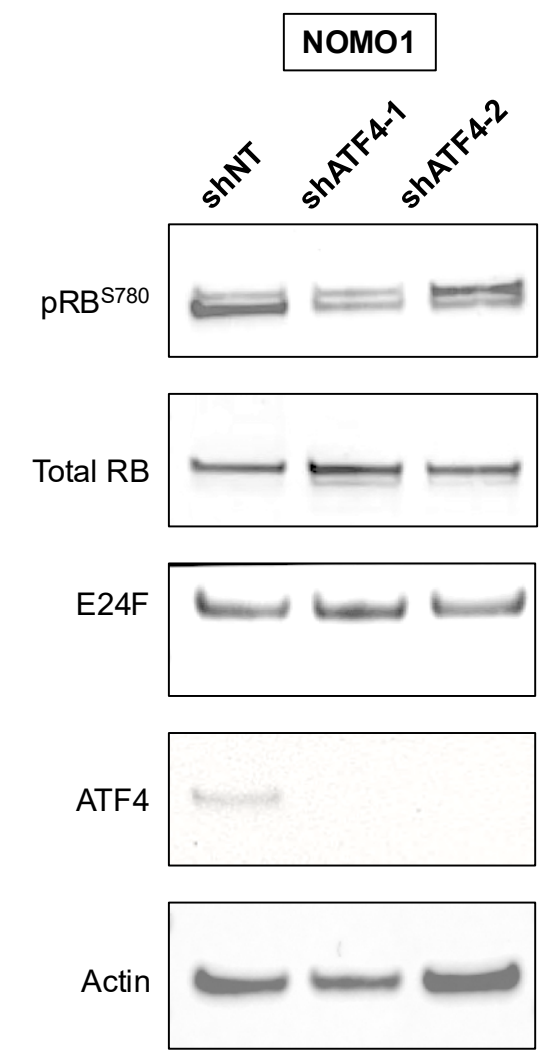

Specific comments: Figure 3D: It is observed that the knockdown of ATF4 mediated by shRNA#2 led to an increase in glycine levels, while shRNA ATF4-#1 reduced glycine levels. The authors should provide an explanation for this discrepancy.

ANSWER: While we are unable to currently provide the reviewer with a definitive explanation for the accumulation of glycine in the shATF4-2 condition relative to the control cells, we speculate that the defect in one-carbon metabolism induced by ATF4 inhibition, which appears to be more severe in the shATF4-2 condition, is the result of compensatory glycine import and/or a block in glycine decarboxylation. For example, a block in glycine production mediated by a defect in SHMT1/2 activity could result in increased uptake of glycine from the extracellular environment. Additionally, our data show that GLDC, which encodes glycine decarboxylase, is a transcriptional target of ATF4. Glycine decarboxylase plays a key role in the glycine cleavage system, which can supply carbon units to one-carbon metabolism. Given that the shATF4-2 is more effective at knocking down ATF4 protein levels and suppressing leukemia cell growth, it is possible that shATF4-2 induces a more potent impact on leukemia cell metabolism that passes a threshold leading to the activation of such compensatory pathways (whereas shATF4-1 is unable to evoke a severe enough metabolic defect). In fact, in NOMO-1 cells, shATF4-2 is more effective at reducing GLDC expression. However, we acknowledge that this explanation is purely speculative at this point.

Figure 3E: It would be valuable to present the protein abundance of PSPH, SHMT1, MTHFD1L, MTHFD2, and ALDH1L2 to support their findings further.

ANSWER: We carried out western blots on lysates from control and ATF4 knockdown lysates using antibodies targeted at the 5 proteins mentioned by the reviewer. However, we did not observe any changes in the expression of PSPH, MTHFD1L, MTHFD2 or ALDH1L2 between shRNA conditions (we were unable to detect a signal for SHMT1). Consistent with these

observations, our proteomics data also showed that there was no significant difference in PSPH, MTHFD1L, MTHFD2, ALDH1L2, or SHMT1 between control and ATF4-targeting shRNA conditions. We have included a sentence in the results section stating that: "Although ATF4 knockdown lead to a significant decrease in steady-state mRNA levels of PSPH, MTHFD1L, MTHFD2, and ALDH1L2, protein levels of each were not impacted by ATF4 inhibition (data not shown)."

Reviewer #3 (Remarks to the Author):

Summary: The authors report on how transcriptional regulators are involved in regulation of cell-cycle dependent changes in protein translation and cellular metabolism, which has not been well studied so far and is novel. Exploiting the metabolism of cancer cells therapeutically is in general a particularly interesting and important field to study. It is known from the literature that ATF4 expression is increased in leukemia-initiating cells and that inhibition of ATF4 leads to decreased disease progression in mouse models of AML harboring KMT2A rearrangements. Furthermore, in AML, data suggests a role of ATF4 in regulating the integrated stress response (ISR), a conserved mechanisms activated upon for instance unfold-protein or mitochondrial stress to halt global protein expression in order to restore cellular homeostasis (https://www.science.org/doi/full/10.1126/scitranslmed.aax2863). The authors report that ATF4 regulates metabolic processes and protein synthesis to drive the cell cycle transition from the G1 to S-phase.

Minor comments:

Figure 1

1) I would suggest being more specific in the text as highlighted in bold: "Next, a doxycycline-inducible shRNA system was utilized to determine the kinetics of anti-leukemia cell growth mediated by genetic ATF4 inhibition."

ANSWER: Thank you for the suggestion and to enhance clarity, we have revised the sentence to read as follows:

"Next, a doxycycline-inducible shRNA system targeting ATF4 was utilized to assess the time-dependent effects of ATF4 knockdown on leukemia cell growth dynamics."

We hope this revision is clearer and more specific.

2) As the authors make a statement about the impact of ATF4 perturbation playing a particular important role in KMT2A-, as well as Normal Karyotype leukemias, it would be interesting to see whether the effects observed for NOMO-1 (MLL-AF9) and MV4-11 (MLL-AF4), as well as OCI-AML3 (NPM1 mutated showing a similar gene expression signature as the MLLr acute leukemias) are not evident in acute leukemia cell lines without KMT2A or NPM1 genetic changes.

ANSWER: We appreciate the reviewer's insightful suggestion to investigate whether the effects of ATF4 perturbation extend beyond KMT2A-rearranged and NPM1-mutated leukemias. To address this, we performed additional experiments using K562 cells, which have neither a KMT2A-rearrangement or a NPM1 mutation. As shown in Supplementary Figure **1G**, ATF4 inhibition leads to a similar decline in cell expansion as in other leukemia cell lines.

3) The authors are validating two different types (inducible and non-inducible) of shRNA mediated genetic knockdown and do get sufficient gene knockdown on protein level. Do these shRNAs also leads to a

downregulation of ATF4 on mRNA expression level by qPCR? Is there a reason why the authors only focused on shRNA mediated knockdown and no sgRNA mediated CRISPR/Cas9 mediated knockout was performed, leading to potentially less off-target effects? Please advise.

ANSWER: We elected to utilize a shRNA-based approach over a CRISP/Cas9-based approach to inhibit ATF4 expression for several reasons: First, our study aimed to investigate the immediate and reversible effects of ATF4 depletion, which is more effectively achieved through RNA interference-based approaches. Second, given that ATF4 is a key regulator of cellular stress responses, complete knockout via CRISPR/Cas9 could lead to compensatory mechanisms or selection for resistant clones over time, potentially confounding our results. Third, we have previously utilized ATF4 targeting shRNAs to show that ATF4 is functionally important to human and mouse AML survival and we wanted to use a similar approach to maintain consistency. Fourth, we have used multiple shRNAs in both mouse and humans, both constitutive and inducible, and all strategies have yielded similar results indicating that our approaches are highly selective for targeting ATF4. Finally, both shRNAs and CRISPR/Cas9-based strategies have potential off-target effects that is directly related to the specific targeting sequences used in either approach. Therefore, each approach and sequence need to be specifically validated for accuracy and potential off-target effects. While we are exploring non-deleting CRISPR-based approaches, such as CRISPRi, we are reserving such approaches for future studies.

4) The authors mention that ATF4 inhibition hinders cell cycle progression. Is there a particular reason they have chosen not to employ well-established FACS analyses like nuclear DNA content distribution? Such analyses could help in assessing the relative distribution of cell cycle phases between shNT and shATF4.

ANSWER: Our BrdU incorporation assay includes a DNA intercalating dye, Propidium Iodide, to simultaneously measure nucleotide incorporation and DNA content distribution. This combination provides a more precise delineation of G0/G1, S and G2/M phases compared to an analysis of nuclear content distribution alone.

Figure label 1H reads "MV4-11 single cell clone from each"

ANSWER: We appreciate the reviewer for bringing this typographical error to our attention. We have corrected the figure label in Figure **1H** to read " MV4-11 single cell clones for each shRNA condition" to ensure clarity and accuracy.

Figure 2

5) To identify downstream pathways that play a role in ATF4 mediated leukemia the authors performed RNAseq analyses for each shRNA condition in NOMO-1 cells, the timepoint chosen was 24 hours, which should be sufficiently long for showing transcriptional changes without being too late as they would have to deal with regulatory feedback signaling effects. They chose a standard statistical cut-off and fold-chance (log2) > 1 and < -1.0. Next they performed pathway enrichment analysis using DAVID. It would be interesting and making the case stronger if using another gene set enrichment analysis platform, such as MSigDb will come up with the same enriched pathways. To statistically validate significance of the observed they performed Benjamini-Hochberg analysis, which is a common analysis performed in these kind of experiments.

ANSWER: Per the Reviewer's suggestion, we performed enrichment analyses of our RNA-seq data utilizing the Molecular Signatures Database (MSigDb), which yielded results consistent with our initial DAVID-based analysis, which further supports the strength of our findings. We have

incorporated these results as supplementary file **4** of the revised manuscript, as well as updated the manuscript text and Materials and Methods section of the manuscript appropriately. Additionally, we note that the MSigDb gene signatures for tRNA aminoacylation are biased toward a select subset of tRNA synthetases, with only 8 of 20 (ARS2, EARS2, GARS1, YARS2, SARS2, DARS2, AARS2, TARS2) being represented in the available gene sets. As a result, the overall enrichment from MSigDb is consistent with our DAVID-based analysis regarding numerous metabolic pathways, however, tRNA aminoacylation is missing from this analysis. We appreciate the reviewer's feedback as we believe that this addition enhances the clarity and rigor of our study.

6) The authors report that the three themes of the enriched pathways were amino acid metabolism, tRNA aminoacylation/charging and UPR signaling. It would also be of importance whether knockdown of ATF4 is also interfering with the MLL gene expression program (such as Hoxa9 and Meis1) leading to downregulation of previously published gene expression signatures (e.g. Günther et al. 2008 Genes and Dev). This would also shed light on the question whether observed effects on cell cycle and survival by knockdown of ATF4 is able to interfere with the MLL induced transcriptional program.

ANSWER: To address whether ATF4 knockdown interferes with the MLL gene expression program, we analyzed the expression of key MLL target genes as outlined in the MLL gene expression signature from Guenther et al. We found that only 1.19% (2/167) of significantly downregulated genes of both shATF4-1 and shATF4-2 overlapped with genes from the MLL gene expression profile. These findings suggest that while ATF4 knockdown affects cell cycle and survival, it does not appear to interfere with the MLL gene expression program. We have updated the revised manuscript to include these findings and have included the analysis as supplementary file **3**.

7) Various metabolic pathways were decreased upon ATF4 knockdown, especially enzymes that play a role in amino acid biosynthesis. The authors also mention a downregulation of multiple amino acid transporters e.g. SLC1A5, some of these reporters have been identified as potential targets to reduce cancer cell growth also in other solid tumors. As metabolic pathways are important for growth of cancer cells in general it would also be of interest for the readership to know that any of these genes (enzymes) or AA reporters account as common essential genes when you e.g. query the various cancer cell lines in the publicly available depmap dataset or are specific hits. Can you also narrow down on specific metabolic pathways where multiple of the downregulated AA transporters are involved?

ANSWER: We thank the reviewer for this valuable suggestion. To address this, we performed a DepMap analysis for the affected genes of amino acid biosynthesis and transport. We have incorporated these results in Supplementary Figure **3** of the revised manuscript, as well as updated the manuscript text and Materials and Methods section of the manuscript appropriately. Additionally, we have updated the revised manuscript to provide clarity on the specific amino acid pathways in which the multiple of the downregulated amino acid transporters as reflected in the extended data.

8) Page 6/55 merged manuscript, please correct typo in sentence: "In addition to amino acid biosynthesis enzymes, RNA-seq data showed that, compared to shNT controls, NOMO-1 cells expressing ATF4-targteing shRNAs displayed lower levels of multiple amino acid transporters"

ANSWER: Thank you for catching this typographical error. Page 6/55 of the manuscript has been corrected to accurately read: "In addition to amino acid biosynthesis enzymes, RNA-seq data showed that, compared to shNT controls, NOMO-1 cells expressing ATF4-**targeting** shRNAs displayed lower levels of multiple amino acid transporters"

9) Figure 2 appears somewhat redundant. In my opinion, it might be more effective to either display only B or C, relocating the other to the supplementary material, or perhaps create a combined plot (since most of the terms are identical, you could just stack the shATF4-1 and shATF4-2 scores). Additionally, the qPCR validations in sections D and E might be better suited for the supplementary material, as they don't seem to offer much new insight. The enrichment analyses already make it quite evident that metabolic pathways are the primary terms in this context.

ANSWER: Thank you for the suggestion. We have relegated the qPCR data from Figure **2** to Supplementary Figure **2**. We tried stacking the enrichment data but could not find an elegant way to present the combined data. We also feel that it is important to show both shRNAs to highlight the consistency of the two distinct shRNAs, and therefore, we left both enrichment analyses. However, if the reviewer feels strongly, we can move one of the comparisons to the supplementary data.

10) It would be great if authors could add some details into the RNA-seq methods section such as RIN cutoff used, Q20, Q30 percentage, the reference genome used, the design used within DESeq2 for the differential expression analysis.

ANSWER: To address this important suggestion, we have included the following additional details on our RNA-seq methods:

- RNA integrity number (RIN) cutoff: Samples with a RIN ≥ 9 were used for sequencing.
- Quality metrics: The sequencing data had an average Q20 of 98.2% and Q30 of 94.8%, ensuring high quality reads.
- Reference genome: Reads were aligned to the GRCh38 reference genome using HISAT2.
- Differential expression analysis: Differential expression analysis was performed using the DESeq2 R package (1.20.0). DESeq2 provide statistical routines for determining differential expression in digital gene expression data using a model based on the negative binomial distribution.

These details have been incorporated into the manuscript in the "*RNA-seq*" section of the Materials & Methods. We appreciate the reviewer's feedback in helping us improve the clarity and reproducibility of our study.

11) I find myself somewhat unclear on the type of analysis employed to arrive at the enrichment results. To my understanding, DAVID typically utilizes a version of a Fisher-test for overrepresentation analysis, contrasting the fraction of genes from a given set (like GO BP sets) in the significantly up- or down-regulated genes against those in the background (or non-significant genes). This leads to an adjusted p-value and a set size for each set but doesn't provide an enrichment score. On the other hand, gene-set enrichment analysis is a rank-based method that accounts for all genes, not only those marked as significantly up- or down-regulated. This ranking, often by adjusted p-value, leads to something akin to a 'normalized enrichment score' (as described in https://www.pnas.org/doi/full/10.1073/pnas.0506580102). While it's probable that the results wouldn't vary much, some clarification in the methods section would be appreciated.

ANSWER: We appreciate the reviewer's request for clarification regarding the type of analysis used to generate the enrichment results. In our study, we utilized DAVID's Functional Annotation Tool, where we input the list of significantly downregulated genes to obtain various annotation

summary results. Within specific annotation lists (Ex: Gene_Ontology) lies numerous functional annotation charts (Ex: GOTERM_BP_DIRECT), which provide the associated biological processes, the number of genes within each process, the p-value, and the Benjamini score.
While DAVID primarily employ's a Fisher's exact test for overrepresentation analysis, we also leveraged the platform's additional Fold Enrichment option, which allows for further contextualization of pathway enrichment beyond p-value significance. We have revised the Materials & Methods section to clarify our approach, and believe this clarification enhances the transparency of our methodology.

ATF4 inhibition affects selective protein translation

12) The quantification of 3000 proteins seems somewhat low, considering that in our experience SILAC-based proteomics in cell lines often yields results in the range of 6000 proteins. While this discrepancy might not directly affect the results presented in this study, it caught our attention. Perhaps the authors could provide some commentary or explanation for this.

ANSWER: We agree with the Reviewer that in large scale proteomics analyses, 3000 quantified proteins is considered a low-end result. The protein quantification from proteomics is influenced by several variables, including but not limited to: (i) sample fractionation, (ii) use of multiple cell types that express complementary proteomes, (iii) use of cell lines that express a plethora of proteins because have loose chromatin regulation, e.g. HeLa, (iv) considering all proteins, including those for which there is no detectable heavy/light quantitative ratio. The relatively low number of identified proteins in our dataset is partially due to our experimental design, in which cells were labeled in heavy isotope SILAC media for only 36 hours. Our shorter labeling period was intentionally chosen to ensure proteomic changes primarily reflected the effects of ATF4 inhibition at the 48-hour timepoint, rather than prolonged metabolic adaptation to heavy isotope incorporation. While our dataset may have fewer quantified proteins compared to other SILAC-based studies, we ensured robust biological and technical replicates to maintain the reliability of our findings. Importantly, the proteins detected in and analyzed in our study included numerous metabolic proteins of interest and showed comparable results in terms of abundance to our RNAseq data, which suggested that the depth of protein detection was sufficient. Nonetheless, we highlight this limitation in the discussion with the following: "due to the limited capabilities of current mass spectroscopy technology, the proteomic studies presented in the study were only able to detect about 15% of the proteome. Thus, it is possible that the translation and abundance of many other proteins, possibly not related to the G1/S transition, are also impacted by ATF4 inhibition."

Response to Reviewers: NCOMMS-23-18711-B

We thank the reviewers for their additional constructive and helpful comments. Each of the reviewer's comments are addressed below and we feel that by doing so, the manuscript has been strengthened. Original comments are in Times New Roman blue font and responses are in Arial black font.

REVIEWER COMMENTS

Reviewer #2 (Remarks to the Author):

The authors have addressed most of my previous comments; however, one issue remains unresolved. The manuscript states that ATF4 knockdown does not affect the protein levels of PSPH, MTHFD1L, MTHFD2, ALDH1L2, or SHMT1, despite apparent transcriptional downregulation. This finding appears inconsistent with multiple prior studies (e.g., PMID: 33646118, PMID: 34303878, PMID: 36051358), which have demonstrated that ATF4 is a key upstream regulator of enzymes involved in serine biosynthesis and the THF cycle. In particular, MTHFD2 protein levels are significantly reduced upon ATF4 depletion in various cellular contexts.

Given this strong precedent, the absence of protein-level changes reported here is unexpected and raises concerns about the sensitivity or execution of the western blot analysis. If the authors intend to retain this conclusion, they should include the supporting western blot data rather than stating "data not shown," to allow readers to evaluate the findings directly. Alternatively, if the data are inconclusive or of insufficient quality, the authors should consider revising or removing the statement to avoid contradicting well-established literature.

ANSWER: We too were surprised by the lack of change in the protein expression of several one-carbon metabolic enzymes previously shown to be reduced by ATF4 inhibition, particularly MTHFD2 – despite the clear decrease in transcript levels. Since our initial analyses were only performed in NOMO-1 cells, we aimed to ensure that these results were not an artifact of a single cell line. Therefore, we generated new stable cell lines expressing doxycycline inducible control- or ATF4-targeting shRNAs in several AML cell lines: NOMO-1, OCI-AML3, MOLM-14 and OCI-AML2. We then repeated western blots on lysates from these new cell lines using newly purchased SHMT1 antibodies as well as antibodies recognizing MTHFD1L, MTHFD2, ALDH1L2, and PSPH. We also included antibodies targeting PHGDH, PSAT1, and ASNS as positive controls. From these analyses, we made the following observations (*which are noted in the results section of the revised manuscript and depicted in Supplementary Figure 3B and 3C*):

1) Consistent with our previous analyses, all four cell lines displayed reduced protein levels of PHGDH, PSAT1, and ASNS upon ATF4 inhibition (Reviewer Figure **1A**).
2) While PSPH expression was marginally reduced by ATF4 inhibition in NOMO-1 and OCI-AML2 cells, we did find that PSPH levels were substantially lower in OCI-AML3 and MOLM-14 cells expressing ATF4-targeting shRNAs compared to controls (Reviewer Figure **1A**).
3) ATF4 knockdown did not impact SHMT1 or MTHFD1L levels in any of the cell lines analyzed (Reviewer Figure **1A** and **1B**).
4) We were unable to detect ALDH1L2 in NOMO-1 cells, however, ATF4 knockdown did lead to a substantial decrease in ALDH1L2 protein levels in OCI-AML3, MOLM-14, and OCI-AML2 cells (Reviewer Figure **1A**).
5) Consistent with our previous analyses, we found that MTHFD2 expression was largely unaffected by ATF4 inhibition in NOMO-1 cells – similar observations were made in OCI-

AML2 cells (Reviewer Figure **1A**). However, we did find that ATF4 knockdown led to an appreciable decrease in MTHFD2 protein levels in OCI-AML3 and MOLM-14 cells (Reviewer Figure **1A**).

As noted by the reviewer, ATF4 inhibition has been shown to reduce MTHFD2 protein levels in several studies (*which we have cited in the revised manuscript*). Therefore, we wanted to ensure that our MTHFD2 antibody was specifically recognizing human MTHFD2 protein. To do so, we employed a CRISPR/Cas9 approach to specifically delete MTHFD2 in NOMO-1 and MOLM-14 cells. As shown in the figure below, MTHFD2 protein levels subside following CRISPR/Cas9-mediated deletion of the *MTHFD2* gene in both cell lines, confirming the specificity of the antibody that we used (Reviewer Figure **1C**). It is possible that if we were to examine later time points post-ATF4 knockdown in our DOX-inducible AML cell lines that we may observe a more sizable decline in MTHFD2 protein (and possibly PSPH, SHMT1 and MTHFD1L). However, the time points that we have chosen for these analyses were selected because they preceded any changes in AML cell biology mediated by ATF4-inhibition. Thus, any changes in MTHFD2 expression (or any other protein) at later time points could be confounded by the alterations in AML cell biology mediated by ATF4 inhibition. We hope that these results allay the Reviewer's concern.

REVIEWER FIGURE 1

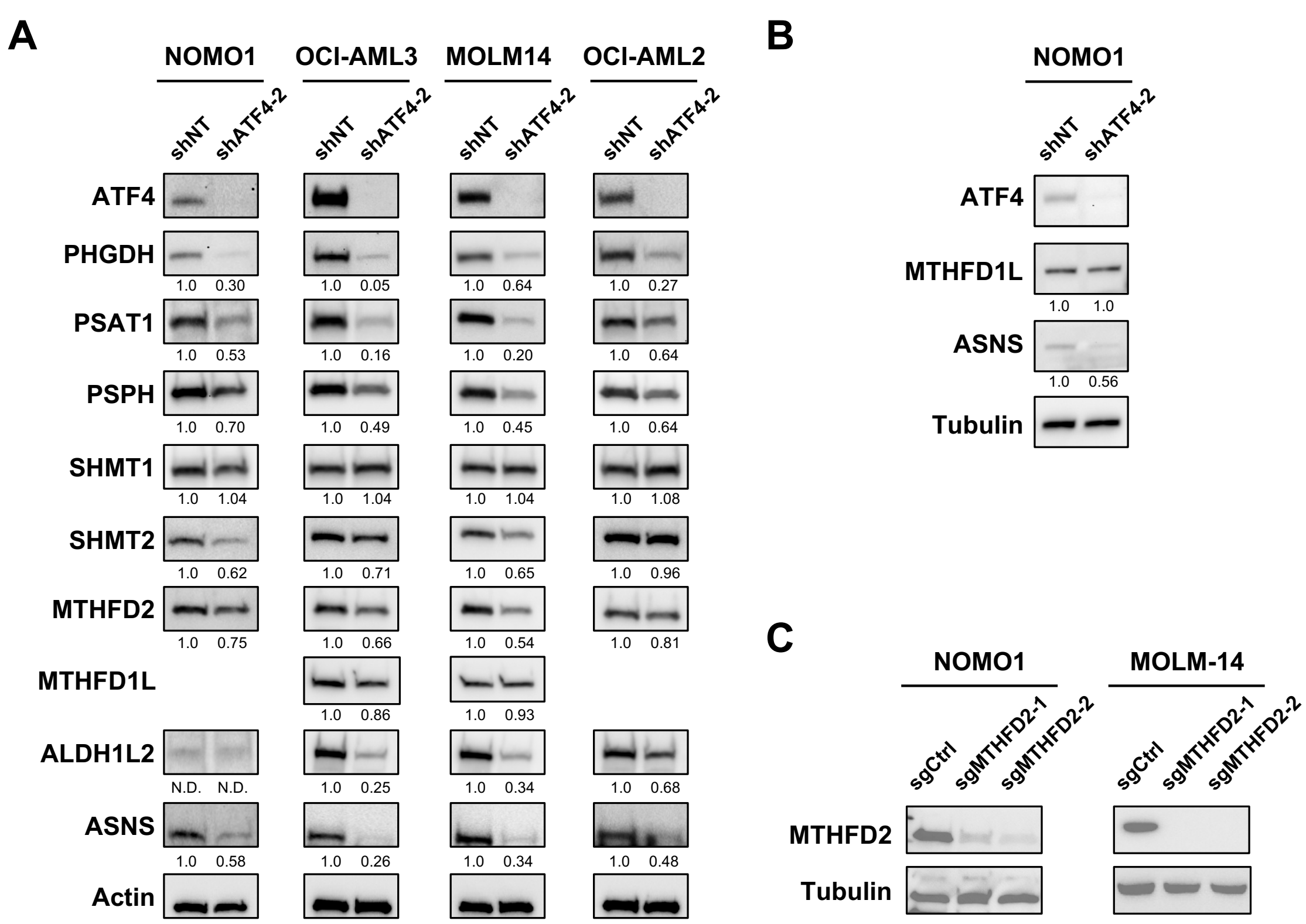

Reviewer #3 (Remarks to the Author): Huhn et al. identified the transcriptional regulator ATF4 as an important player in generating nucleotides and protein machinery needed for initiation and timing of DNA replication in acute leukemia. Characterization and understanding of deregulated metabolic pathways have previously been identified as a vulnerability in acute leukemia that can be exploited for leukemia therapy (e.g. IDH inhibition in AML, PEG-Asparaginase in B-ALL). This study focusses on an important and emerging topic that could play a role in understanding the mode of action of several drugs that target

leukemia metabolism and suggests ATF4 inhibition as a potential novel therapeutic option.

Specific Comments: The authors show in Figure 1 that by using a doxycycline-inducible shRNA approach they achieve a complete genetic knockout (at least in NOMO-1) on protein level. Furthermore, they observe a cell cycle arrest in G1, as well as apoptosis. In line with previous reviewer's comments I am wondering why the authors have not additionally employed a CRISPR/Cas9 approach and whether ATF4 is also showing a reduced expression on transcript level? The authors have not fully answered this question in the rebuttal letter. Please advise.

ANSWER: As shown in Supplementary Figures 2C-2E, steady-state levels of *ATF4* mRNA are reduced upon expression of ATF4 shRNAs. Second, in addition to the 5 reasons that we stated in our previous response, let us clarify the central reason why we elected to apply an shRNA-mediated approach over a CRISPR/Cas9 approach:

The central message of this manuscript is that ATF4 regulates genes encoding enzymes that support amino acid biosynthesis and protein synthesis (tRNA synthetases) to generate the biomass and machinery to drive the G1/S transition of AML cells. Because many cellular metabolic processes (including the synthesis of many amino acids and nucleotides) and protein synthesis are strongly impacted by cell cycle arrest, it was paramount that we carry out all ATF4 target gene expression, metabolomic and protein synthesis studies at a time point before any changes in cell cycle arrest that would result from ATF4 inhibition. As stated in the manuscript, using our DOX-inducible shRNA system, ATF4 levels begin to decline at 16-24 hours post-DOX followed by changes in cell cycle at approximately 60-72 hours post-DOX. This allowed us a reliable and predictable 24-36 hour window to assess the impact of ATF4 inhibition on gene expression, cellular metabolism and protein synthesis.

In our experience, the kinetics of CRISPR/Cas9-mediated deletion take significantly longer to reduce protein levels compared to shRNA-mediated knockdown of protein levels. Furthermore, Depmap data indicates that CRIPSR/Cas9-mediated deletion of ATF4 strongly antagonizes AML cell growth (included in Supplementary Figure 4). A central issue when employing CRISPR/Cas9 loss-of-function technology, particularly in a setting where acute deletion of the target gene negatively impacts growth, is the lack of complete deletion and/or re-emergence of undeleted cells. These CRISPR/Cas9 variables create a less predictable and reliable window for carrying out our functional studies compared to our DOX-inducible shRNA-mediated approach.

To emphasize this point and to directly address the reviewer's request, we generated lentiviral vectors co-expressing Cas9 and a ATF4-targeting sgRNA that has previously been shown to delete ATF4 in other human cells (PMID: 37286597; 33483422; 31023583; 28494858). We then used these constructs to delete ATF4 in both NOMO-1 and MOLM-14 cells. As shown in the Reviewer Figure **2** below, CRISPR/Cas9-targeting of ATF4 reduced but did not abolish ATF4 expression at early time points post-transduction. Furthermore, at later time points, we observed the re-emergence of undeleted cells. Consistent with these observations, we see that deletion of ATF4 initially reduces AML cell expansion, however, growth rates ultimately return (Reviewer Figure **2**). Thus, given the unpredictable and incomplete inhibition of ATF4, we elected to forego the use of CRISPR/Cas9 technology to target ATF4 in this study.

REVIEWER FIGURE 2

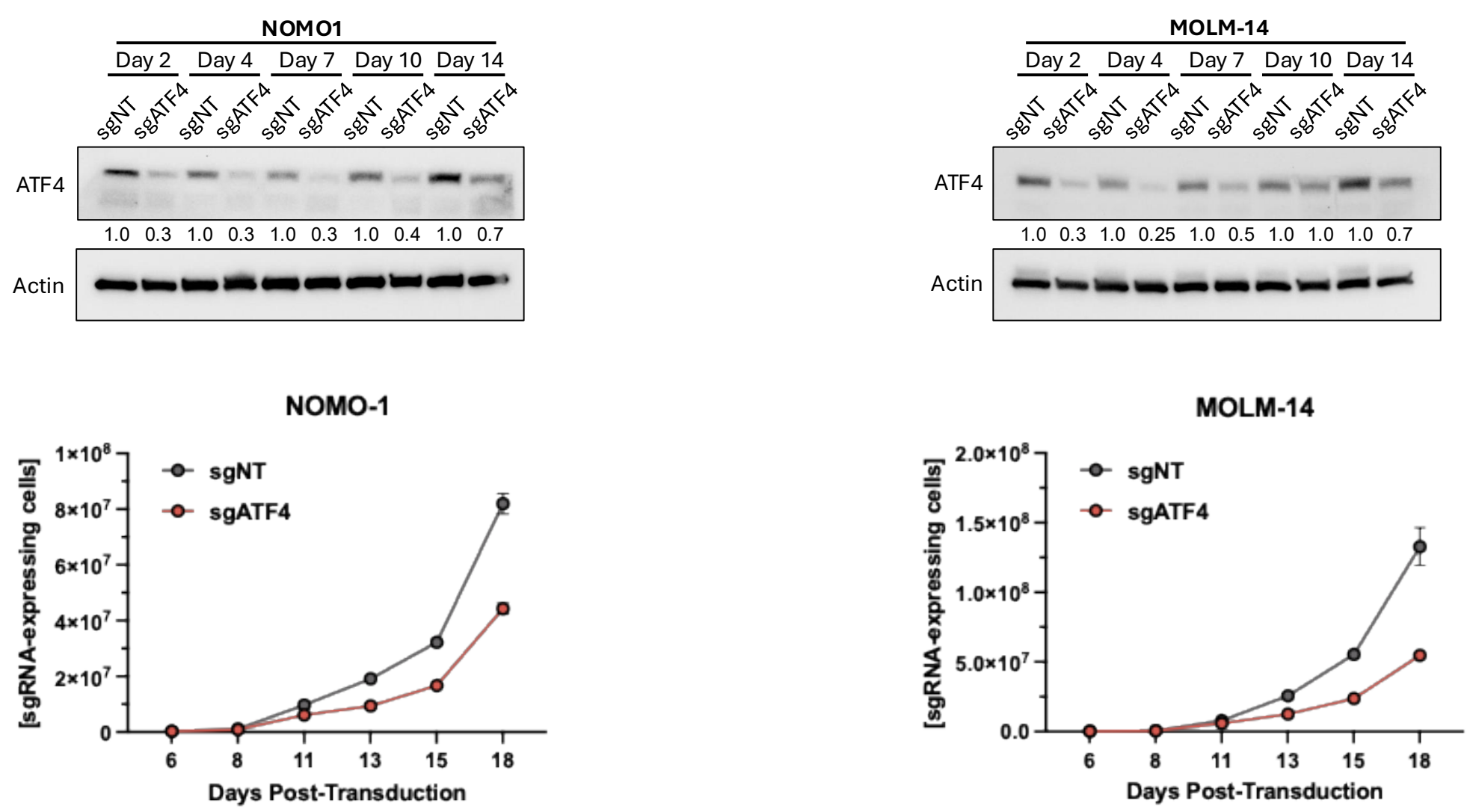

Did the authors observe a CD11b shift and cytological changes as shown in Supplementary Fig. 1 (i.e. Cytopins) in other cell lines as well or were experiments only performed in one cell line?

ANSWER: We have performed myeloid surface marker and cytological analyses in multiple cell lines that we have included in our revised submission. Briefly, upon ATF4 knockdown, we observed CD11b shifts in NOMO-1 (Figure 1J), OCI-AML3 (Supplementary Figure 1J), and MOLM-14 (Supplementary Figure 1K). We also observed increases in another marker of mature myeloid cells, CD14 in OCI-AML3 (Supplementary Figure 1J) and OCI-AML2 (Supplementary Figure 1L) upon ATF4 knockdown. Consistent with these observations, we also found that NOMO-1 (Figure 1K), OCI-AML3 (Supplementary Figure 1M), MOLM-14 (Supplementary Figure 1N), and OCI-AML2 (Supplementary Figure 1O) cells expressing ATF4-targeting shRNAs display cytological changes consistent with myeloid maturation. The results have been included in the revised manuscript in the figures designated above.

In Figure 2 the authors nicely show in a GSEA analysis of RNAseq data after shRNA mediated ATF4 knockdown that transcripts genes fell into three major categories: amino acid metabolism, tRNA aminoacylation/charging, and UPR signaling. The following experiments show that ATF4 inhibition led to a downregulation of genes involved in various pathways, e.g. one-carbon metabolism, glutamate and aspartate metabolism. It would be helpful, assessing the impact of the individual genes, if the authors could add a column with the DepMap Score to the analyzed dependency data in Supplementary Fig. 3

ANSWER: We thank the reviewer for the additional suggestion for what is now Supplementary Figure **4**. We have included the average Chronos scores for each gene across the AML cell lines, the combined hematological malignancy (lymphoid and myeloid) cell lines (Heme), the solid cancer cell lines (Solid) and the non-cancerous (NC) cell lines. We find this recommendation to be highly informative and strengthens the revised manuscript.

What method was used for the analysis you show in Figure 3 C-V. It is not mentioned in the text nor the Figure legend.

ANSWER: Our apologies for the oversight and thank you for pointing out this omission. Figures 3C-V represent bar graphs of the HILIC-MS-mediated quantification of TPMs extracted from each NOMO-1 cells expressing shNT, shATF4-1, or shATF4-2. We have now included this information in the Figure 3 legend. We have also indicated in the methods section that unpaired, two-tailed *t*-tests were applied to compare control with each experimental shRNA conditions (shNT versus shATF4-1 or shNT versus shATF4-2).

It looks like all of these low-throuput validation experiments were performed in one cell line, NOMO-1? Did you assess the impact on these metabolites in other cell lines as well to rule out that we are looking at a cell-line specific effect?

ANSWER: The Reviewer raises an important point that is a concern in many published studies. First, as part of our response to Reviewer 2, we repeated many of our western blots in multiple cell lines to confirm that changes in many ATF4 proteins are consistent across several cell lines. Second, we have seen that ATF4 inhibition promotes cell cycle arrest, myeloid maturation and subsequent cell death in multiple cell lines. Third, to ensure ATF4 inhibition consistently alters AML cell metabolism, we have conducted heavy-carbon glucose and heavy-carbon glutamine tracing studies in three cell lines (NOMO-1, OCI-AML3, and NOMO-1), the results of which are included in the revised manuscript (Figures 4 and 5 and Supplementary Figures 6-8). In all three cell lines, ATF4 inhibition consistently showed decreases in serine and glycine synthesis, increases of glucose-derived carbons in the TCA, and decreases in the biosynthesis of several amino acids arising glutamine anaplerosis. Lastly, we have conducted the DNA fibre and Repli-seq studies in two AML cell lines, NOMO-1 and OCI-AML3. While, not surprisingly, we have observed some subtle cell-line specific effects (documented throughout the revised manuscript), our central observations regarding the role of ATF4 in AML are consistent across multiple cell lines. Moreover, our results across multiple cell lines support our central conclusion that AML cells rely on ATF4 to drive the expression of genes that support amino acid and nucleotide biosynthesis as well as the selective translation of proteins that ensure proper DNA replication firing and timing.

In several paragraphs the results are purely displayed in a descriptic manner. E.g. starting from line 326. It would be important that the authors could interpret their observed results in order to draw more definite conclusions.

ANSWER: Thank you the suggestion to enhance the clarity and implications or our results. From our estimation of the previous submission, line 326 begins at subsection: "**ATF4 regulates tRNA aminoacylation**". In the revised manuscript, we have added the following conclusive statements to the indicated subsections throughout the remainder of the Results section (line numbers of the new manuscript have been estimated and included here).

A) Regarding the section: "**ATF4 regulates tRNA aminoacylation**" we have added the following to:

1. <u>Lines 343-346</u>. "Increasing concentrations of these amino acids were unable to mitigate the anti-leukemia effects of ATF4 inhibition in NOMO-1, OCI-AML3, or MV4-11 cells (Supplementary Figure 9A-9C) suggesting that ATF4 supports AML cell biology through mechanisms additional to promoting amino acid biosynthesis."

2. Lines 359-360. “These results suggest that ATF4, through regulation of tRNA synthetase expression, may regulate protein synthesis.”

B) Regarding the section: “**ATF4 inhibition affects the translation of select proteins** ” we have added the following to:

1. Lines 375-381. “Expression of 53 of the 111 differentially expressed proteins (28 that decreased) correlated with changes in mRNA expression mediated by ATF4 inhibition suggesting that these are direct transcriptional targets of ATF4 (Supplementary Figure 10C). In fact, many of the 28 proteins that were decreased at both the mRNA and protein level by ATF4 inhibition are involved in amino acid metabolism and tRNA aminoacylation (Supplementary Figure 10D). Of the 50 significantly decreased proteins, only 28 (56%) of could be explained by decreased RNA expression (Supplementary Figure 10E).”

2. Lines 390-392. “Further confirming that ATF4 does not regulate global protein translation, only 8.4% of detected labeled proteins displayed a significant change in protein turnover upon ATF4 inhibition.”

C) Regarding the section: “**DNA replication speed and timing are altered by ATF4 inhibition**”, specifically related to the DNA fibre assays, we have added the following to:

1. Lines 422-424. “In both OCI-AML3 and NOMO-1 cells, ATF4 inhibition resulted in significantly shorter fiber lengths, indicating a reduction in DNA replication speed (Figure 7A and 7B)”.

D) Regarding the section: “**DNA replication speed and timing are altered by ATF4 inhibition**”, specifically related to the Repli-seq analyses, we have added the following to:

1. Lines 433-435. “The most profound alterations in RTI by ATF4 inhibition, which were largely regions of premature DNA replication, occurred on the X chromosome of both NOMO-1 and OCI-AML3 cells (Figure 7D and Supplementary Figure 12B).”

2. Lines 449-450. “Overall, these results indicate that ATF4 inhibition perturbs regional DNA replication timing that can result in either a delay in replication timing or premature replication.”

3. Line 458. “These data indicate that ATF4 inhibition is disrupting replication origin firing.”

4. Lines 462-470. “The shorter TSs could indicate that ATF4 is required for proper termination of DNA replication possibly by regulating replication fork speed or stability. Constant timing regions (CTR) are large regions of DNA that are replicated by the coordinated firing of multiple origins of replication (i.e. IZs) and can occur in both early and late stages of DNA replication. Further highlighting the requirement of ATF4 to maintain proper DNA replication timing, ATF4 inhibition also significantly altered the length of constant timing regions (CTR) in both NOMO-1 ($shNT_{mean}$= 663298 bp, $shATF4\text{-}1_{mean}$= 550412 bp, $shATF4\text{-}2_{mean}$= 532598 bp) and OCI-AML3 cells ($shNT_{mean}$= 723622 bp, $shATF4\text{-}2_{mean}$= 664257 bp) (Figure 7G and Supplementary Figure 12F).”

5. Lines 472-475. “Collectively, these data indicate that ATF4 plays a critical role in coordinating the production of certain amino acids and nucleotides with selective protein synthesis to facilitate the G1-to-S-phase transition and ensure the proper timing of DNA replication in AML cells.”

We hope that these conclusive statements provide greater clarity and interpretation to the results section.

In Figure 6 the authors mention that downregulation of ATF4 by shRNA leads to a decreased mRNA expression of AIMP2 and EEF1E1, two proteins involved in the formation of the aminoacyl-tRNA synthetase complex. It would be interesting to find out whether formation of the tRNA synthetase complex is impacted by genetic inhibition of ATF4. As the authors have accessibility to MS, they could prove this by performing a MS-Immunoprecipitation of the complex in control and ATF4 knockdown cells to investigate whether the composition of the complex is altered after ATF4 knockdown.

ANSWER: While we did observe decreased mRNA expression of the multi-aminoacyl-tRNA synthetase complex components AIMP2 and AIMP3 (EEF1E1), our proteomic analyses did not show a significant decrease in protein levels of either component between the control and ATF4 knockdown conditions. However, as shown in Supplementary Figure 10C, we did observe that mRNA and protein levels of several tRNA synthetases, such as AARS1, CARS1, GARS1, TARS1, YARS1 and WARS was significantly reduced. This data suggests that decreases in these tRNA synthetases, and not alterations multi-aminoacyl-tRNA synthetase complex formation, that contributes to the protein translation defects mediated by ATF4 inhibition.

Figure 7

Indeed, detection of 2,918 proteins is a low-end result in comparison to our experiences with MS proteomics analyses. However, the authors have addressed this and given a possible explanation for this in their rebuttal letter.

ANSWER: We appreciate the Reviewer’s acceptance of our response, thank you.
