# Supplementary material for: ATF4 coordinates amino acid and nucleotide synthesis with selective protein translation to ensure proper DNA replication timing in leukemia cells
Source: Nat Commun. 2026 Jun 20;17:7760. doi: 10.1038/s41467-026-74324-1 (PMC13434596; doi:10.1038/s41467-026-74324-1)
Supplement: Supplementary file 3 [file 41467_2026_74324_MOESM1_ESM.pdf]

Supplementary Figures and Legends for:

ATF4 coordinates amino acid and nucleotide synthesis with selective protein translation to ensure proper DNA replication timing in leukemia cells

Jacklyn M. Huhn[1,2*], Liana Valin[3*], Mary Basse[3], Judith Sokei[3], Nishanth Gabriel[3], Esteban Martinez[1], Joice S. Kanefsky[1,2], Stephanie Stransky[4], Adrienne Dorrance[5], Ramiro Garzon[5], Daniela Di Marcantonio[1], Tomasz Skorski[2], Aaron R. Goldman[6], Hsin-Yao Tang[6], Samuele Cortellazzi[7], Orsola di Martino[7], John Krais[3], Simone Sidoli[4], Francesca Ferraro[3#], David L. Wiest[1#], and Stephen M. Sykes[3#]

Affiliations:

1- Fox Chase Cancer Center, Philadelphia, United States of America.

2- Temple University Lewis Katz School of Medicine, Philadelphia, United States of America.

3- Washington University School of Medicine, Saint Louis, United States of America.

4- Albert Einstein College of Medicine, New York, United States of America.

5- Huntsman Cancer Institute at the University of Utah, Salt Lake City, United States of America.

6- Wistar Institute, Philadelphia, United States of America.

7- University of Parma, Parma, Italy.

*Authors with equal contributions

#Co-corresponding authors

Lead contact: Stephen M. Sykes, PhD
Department of Pediatrics, Washington University School of Medicine in St. Louis
425 S. Euclid Ave, MSC 8208-16-05212B, St. Louis, MO 63110
Email: s.m.sykes@wustl.edu

Supplementary Figure 1

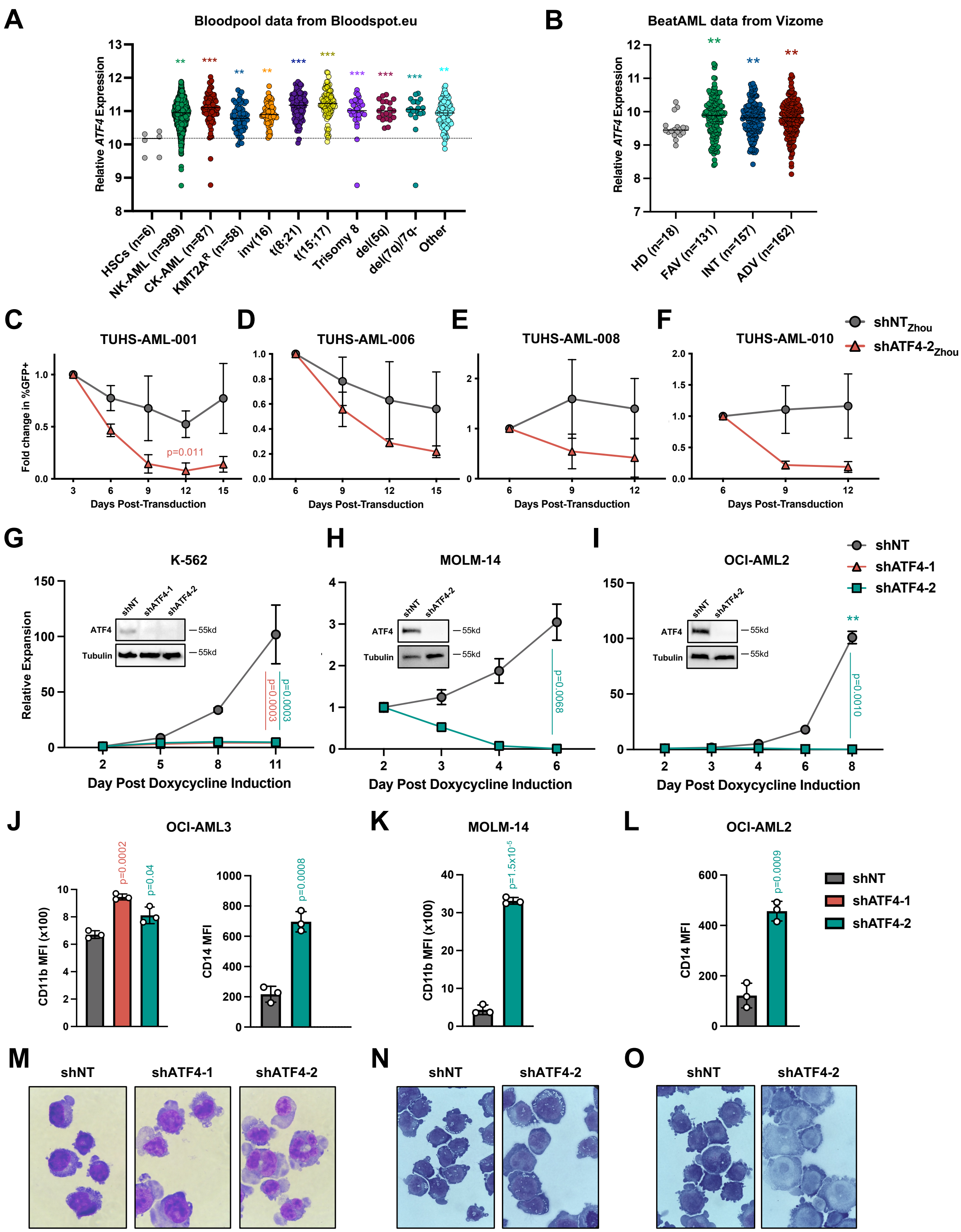

Supplementary Figure 1. A) *ATF4* expression data across healthy HSPC and the indicated genetic subtypes of AML. Data retrieved from the TCGA via bloodspot.eu. The sample size of each condition is indicated in the x-axis. Statistics are derived from a two-sided, unpaired t-test (95% CI) comparison between healthy HSCs (Control) and each AML subtype, **P ≤ 0.01; ***P ≤ 0.001; ****P ≤ 0.0001. Exact p-values are listed in the Source Data file. Adjustments for multiple comparisons were not made. (**B**) Expression data of *ATF4* (from BeatAML) in healthy bone marrow derived mononuclear cells (MNC) compared to samples from patients with a favorable, intermediated, or adverse prognosis data. Statistics are derived from a two-sided, unpaired t-test (95% CI) comparison between healthy donors (HD, control) and each AML risk category, **P ≤ 0.01. Exact p-values are listed in the Source Data file. Adjustments for multiple comparisons were not made. **C-F**) Cryopreserved patient-derived samples were thawed, transduced with lentiviruses co-expressing GFP and shNT or shATF4 and 3-6 days after transduction, samples were then monitored for changes in %GFP by flow cytometry over the following 15 days. Data represent the mean ± SD of three technical replicates from the only biological replicate performed per patient sample. Statistics are derived from a two-sided, unpaired t-test using Welch's correction (95% CI) comparing the shNT control and shATF4-2_{Zhou} conditions at each time point. Multiple comparisons were carried out using a two-stage linear Benjamini-Krieger-Yekutieli (BKY) procedure (exact p- and q- values are listed in Source Data File). **G-I**) K562 (G), MOLM-14 (H), and OCI-AML2 (I) expressing inducible shNT, shATF4-1, or shATF4-2 were administered DOX and then analyzed for RFP+, live cell counts by flow cytometry on the indicated days post-DOX. *Inset*, western blot analysis of ATF4 protein expression at 24 hours post-DOX. For panel **G-I**, data represent the mean ± SD of three technical replicates. Each experiment was repeated in three independent, biological replicates. Statistics are derived from a two-sided,

unpaired t-test using Welch's correction (95% CI) comparing the shNT control with the shATF4-1 (pink p-values) or shATF4-2 (teal p-values) conditions. Multiple comparisons were carried out using a two-stage linear Benjamini-Krieger-Yekutieli (BKY) procedure (p- and q- values are listed in Source Data File). **J**) OCI-AML3 cells from the indicated shRNA conditions were analyzed by flow cytometry for CD11b (*left panel*) or CD14 (*right panel*) MFI (median fluorescence intensity) at 72 hours post-DOX treatment. **K**) MOLM-14 cells from the indicated shRNA conditions were analyzed by flow cytometry for CD11b MFI at 48 hours post-DOX treatment. **L**) OCI-AML2 cells from the indicated shRNA conditions were analyzed by flow cytometry for CD14 MFI at 72 hours post-DOX treatment. For panels **J-L**, data represent the mean ± SD of three technical replicates. Each experiment was repeated in three independent, biological replicates. Statistics are derived from a two-sided, unpaired t-test (95% CI) comparison between the shNT control and the shATF4-1 (pink asterisks) or the shATF4-2 (teal asterisks) conditions. Adjustments for multiple comparisons were not made. **M-O**) OCI-AML3 (M), MOLM-14 (N), and OCI-AML2 (O) cells from the indicated shRNA conditions were stained with Wright-Giemsa and analyzed by light microscopy for morphological changes at 3-4 days post-DOX (100X magnification). Images represent one slide from a replicate of three independent, biological replicates. All Source data for panels **A-L** are provided as a Source Data file.

Supplementary Figure 2

A

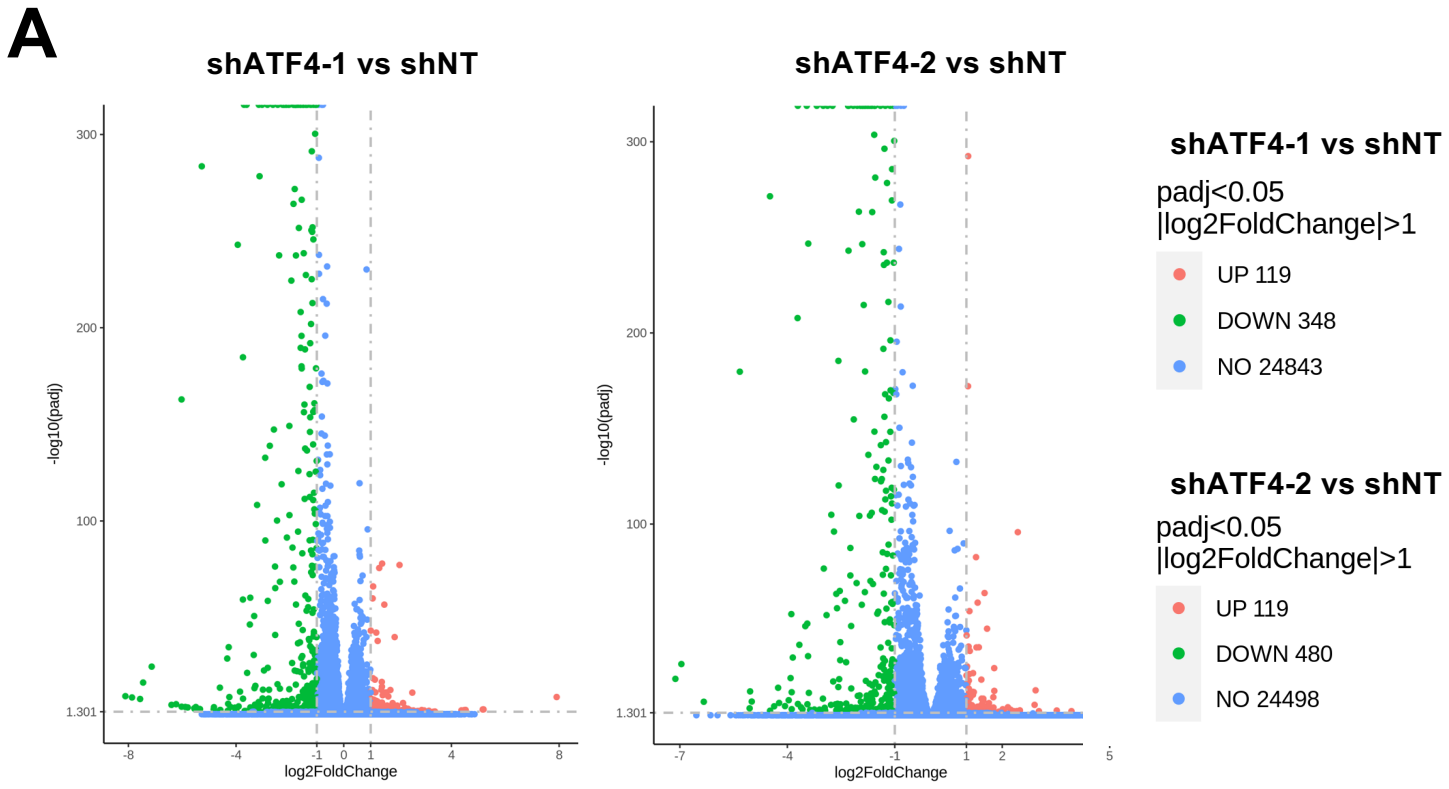

B

Gene Name	Fold Change
Atf4	-1.844
Phgdh	-1.385
Psph	-1.064
Shmt2	-1.337
Mthfd2	-1.116
Aldh1l2	-2.640
Cth	-4.654
Asns	-1.370
Bcat1	-2.177
Pycr1	-1.474

C

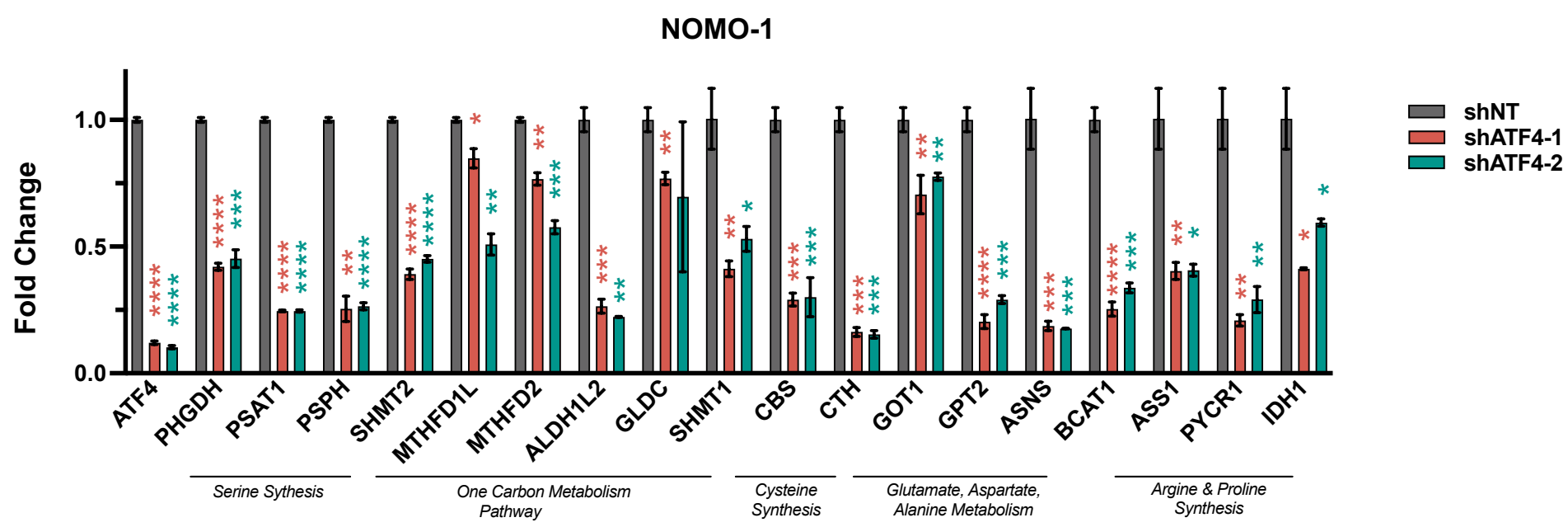

D

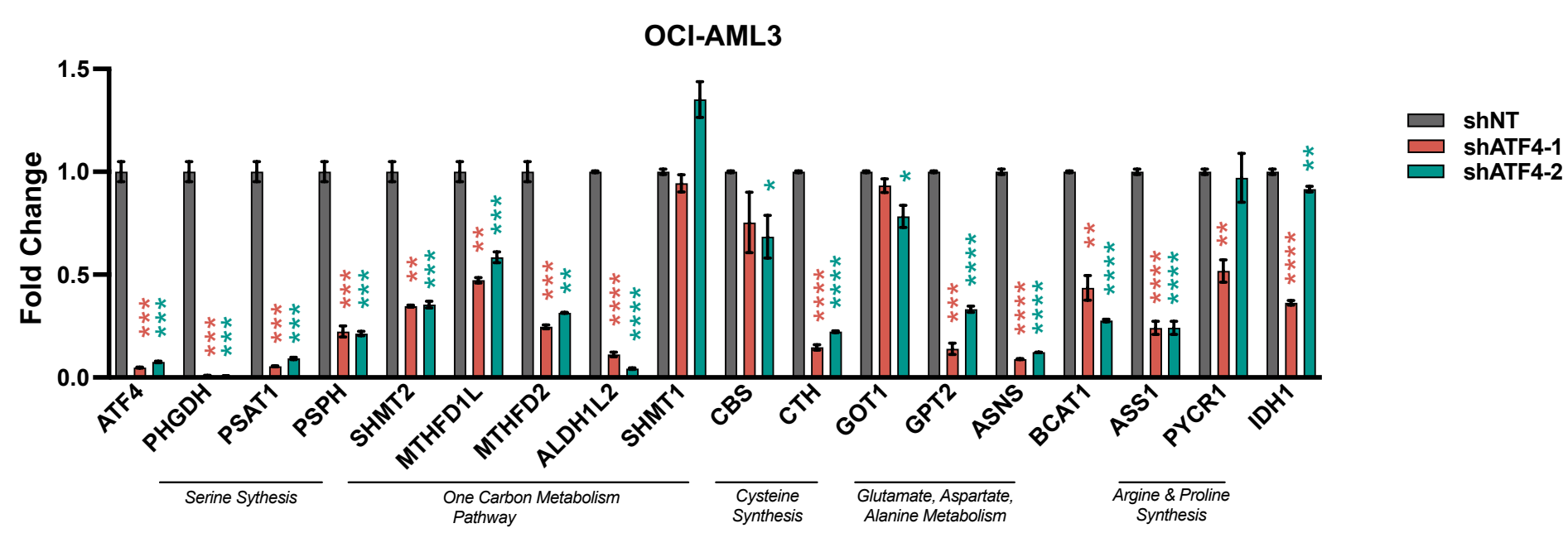

E

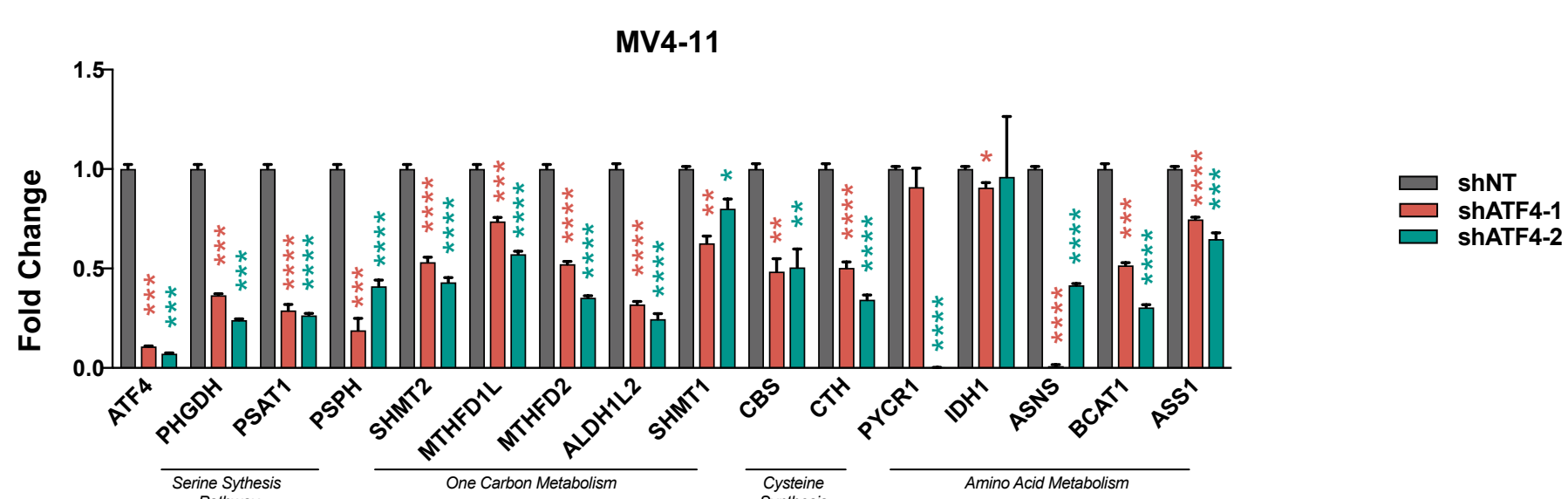

Supplementary Figure 2. A) RNA-seq volcano plot analysis of DOX-treated NOMO-1 cells expressing the indicated shRNAs. Significantly upregulated and downregulated mRNA transcripts are labeled in red and green, respectively, with a statistical cut-off of $p_{adj} < 0.05$ and fold change (log_2) > 1.0 and < -1.0. **B**) Differential expression fold change of specified genes in Atf4-shRNA-expressing MLL-AF9 cells compared with shNT controls. **C-E**) qPCR analysis of indicated metabolic enzymes in NOMO-1 (C), OCI-AML3 (D) and MV4-11 (E) expressing the indicated shRNAs. For panels **C-E**, all graphed data represent the mean ± SD of three technical replicates from one biological replicate. Three independent, biological replicates were carried out for each cell line. Statistics are derived from a two-sided, unpaired t-test (95% CI) comparison between the shNT control and the shATF4-1 (pink asterisks) or the shATF4-2 (teal asterisks) conditions. Adjustments for multiple comparisons were not made. Asterisk key: *P ≤ 0.05; **P ≤ 0.01; ***P ≤ 0.001; ****P ≤ 0.0001. All Source data are provided as a Source Data file.

Supplementary Figure 3

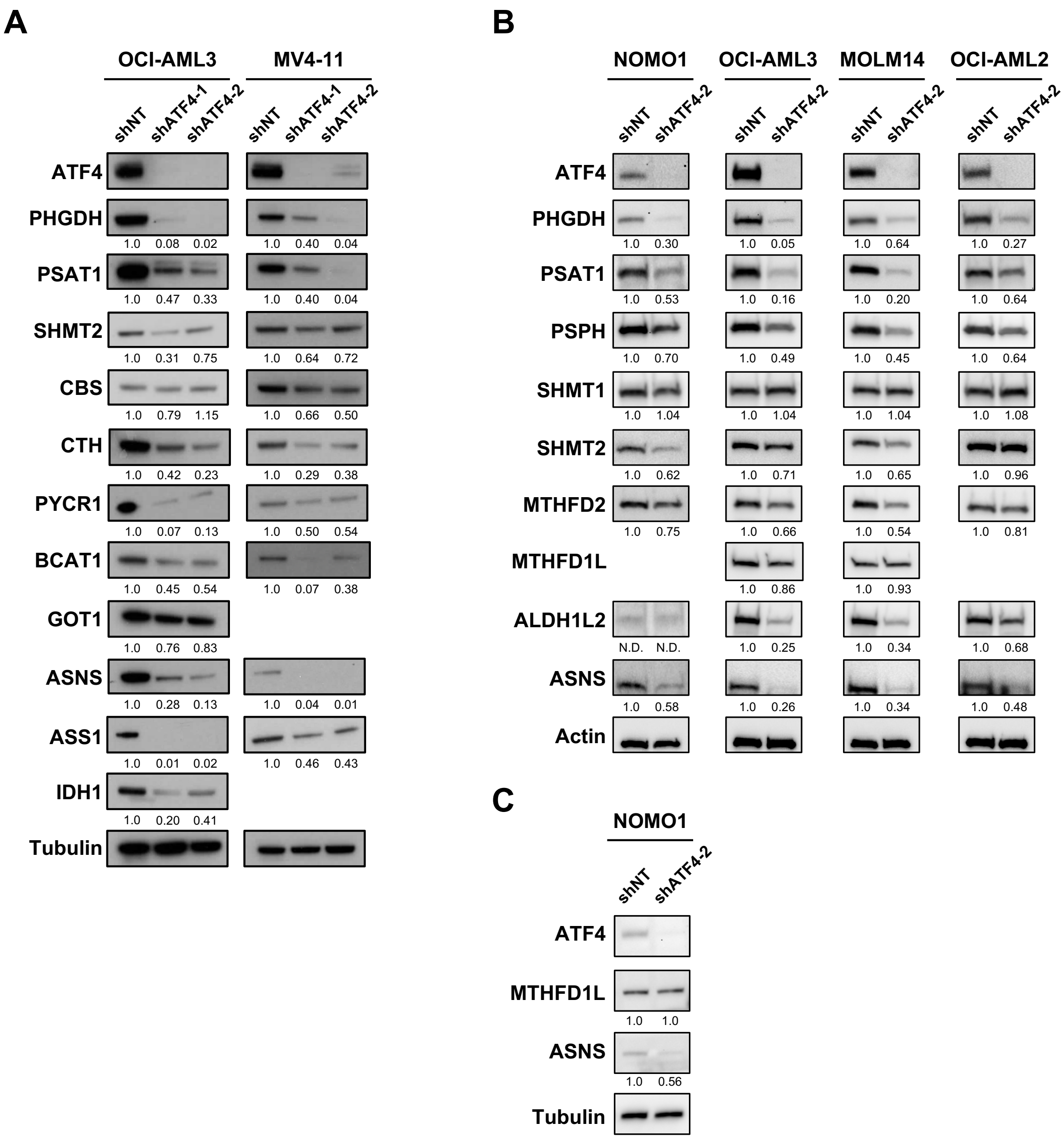

Supplementary Figure 3. A) OCI-AML3 or MV4-11 expressing control or ATF4-targeting shRNAs were subjected to western blot with the indicated antibodies. Quantified proteins levels were normalized to tubulin. Two independent, biological replicates were carried out for each cell line. **B**) NOMO-1, OCI-AML3, MOLM-14 and OCI-AML2 expressing control or ATF4-targeting shRNAs were subjected to western blot with the indicated antibodies. Quantified proteins levels were normalized to Actin. Four independent, biological replicates were carried out for each cell line. **C**) NOMO-1 cells expressing control or ATF4-targeting shRNAs were subjected to western blot with the indicated antibodies. Quantified proteins levels were normalized to tubulin. Two independent, biological replicates were carried out for each cell line. Uncropped blots are located in the Source Data File. Images represent one replicate of three independent, biological replicates. All Source data are provided as a Source Data file.

Supplementary Figure 4

Gene Name	Function	Selectivity	Chronos Score			
			AML	Heme	Solid	NC
ATF4	Unfolded Protein Response	No	-0.79	-0.82	-0.71	-0.70
PHGDH	de novo Serine Synthesis	No	-0.09	-0.06	-0.10	-0.09
PSAT1	de novo Serine Synthesis	No	-0.26	-0.23	-0.25	-0.28
PSPH	de novo Serine Synthesis	No	-0.17	-0.16	-0.15	-0.17
SHMT1	Serine-Glycine Conversion	No	-0.12	-0.11	-0.14	-0.14
SHMT2	Serine-Glycine Conversion	Selective	-0.18	-0.19	-0.10	-0.17
MTHFD1L	One-Carbon Metabolism	Selective	-0.12	-0.11	-0.05	0.00
MTHFD2	One-Carbon Metabolism	Selective	-0.14	-0.14	0.00	-0.11
ALDH1L2	One-Carbon Metabolism	No	-0.01	-0.03	-0.02	-0.04
GLDC	Glycine Decarboxylase	No	-0.03	-0.04	-0.02	-0.03
CBS	de novo Cysteine Synthesis	Selective	-0.10	-0.05	-0.02	0.01
CTH	de novo Cysteine Synthesis	No	0.10	0.10	0.08	0.08
GOT1	Glutamine--Oxaloacetate Transaminase	No	-0.05	-0.05	-0.03	-0.06
GPT2	Glutamine--PyruvateTransaminase	No	-0.05	-0.07	-0.11	-0.15
ASNS	de novo Asparagine Synthesis	Selective	-0.17	-0.17	-0.28	-0.28
ASS1	de novo Arginine Synthesis	No	-0.24	-0.19	-0.23	-0.18
BCAT1	Branched Chain Amino Acid Transaminase	No	0.03	0.01	0.03	0.01
PYCR1	de novo Proline Synthesis	No	-0.09	-0.09	-0.09	-0.08
IDH1	TCA cycle enzymes	No	-0.10	-0.08	-0.10	-0.08
SLC1A4	Transporter: Alanine, Serine, Cysteine, Threonine	No	-0.01	-0.01	0.03	0.02
SLC1A5	Transporter: Glutamine (primary), Alanine, Serine	No	-0.21	-0.25	-0.20	-0.17
SLC6A9	Transporter: Glycine	No	-0.20	-0.18	-0.17	-0.14
SLC7A1	Transporter: Arginine, Histidine, Lysine	Selective	-0.54	-0.52	-0.23	-0.32
SLC7A2	Transporter: Arginine, Lysine, Ornithine	No	0.13	0.13	0.13	0.09
SLC7A5	Transporter: Phenylalanine, Tyrosine, Leucine, Histidine, Methionine, Tryptophan, Valine, Isoleucine, Alanine	Selective	-0.42	-0.45	-0.27	-0.25
SLC7A11	Transporter: Cysteine, Glutamate	No	0.03	-0.07	0.07	0.06
SLC38A1	Transporter: Glutamine	Selective	-0.12	-0.21	-0.10	-0.08
SLC38A2	Transporter: Alanine, Serine, Proline, Glutamine	Selective	-0.57	-0.43	-0.36	-0.26
SLC43A1	Transporter: Isoleucine, Leucine, Methionine, Phenylalanine, and Valine	No	0.04	0.05	0.00	0.01

Supplementary Figure 4. Tabulation of amino acid biosynthesis enzymes and transporters whose steady-state mRNA expression is decreased by ATF4. The column third from the left lists the Depmap dependency based on CRISPR DepMap Public 24Q4+Score, Chronos. Last four columns list the average Chronos scores of cell lines considered an AML, a hematological malignancies (Heme), a solid cancer (Solid), or non-cancerous (NC).

Supplementary Figure 5

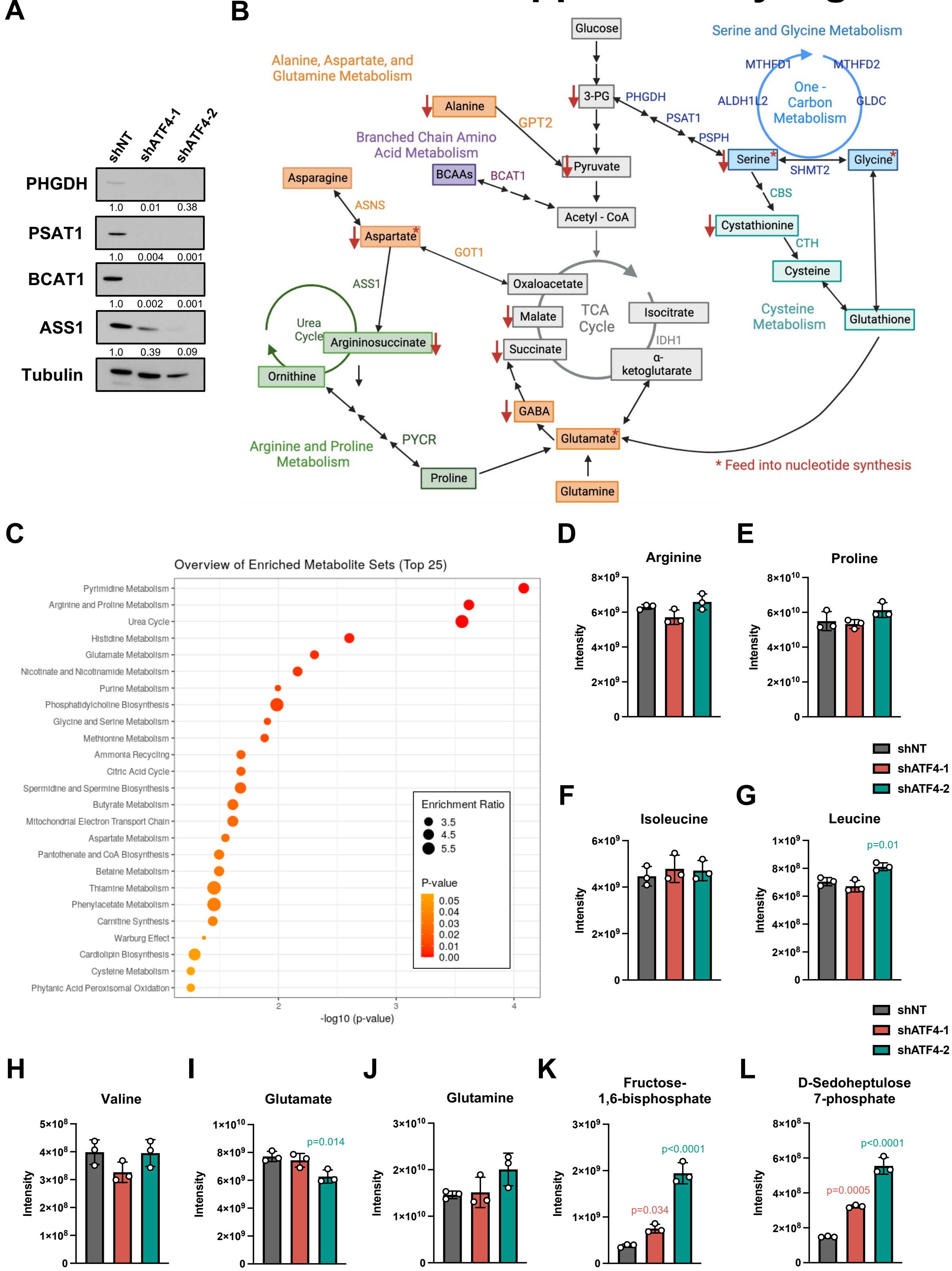

Supplementary Figure 5. A) NOMO-1 cells from each of the indicated shRNA conditions were subjected to western blot with the denoted antibodies. Quantified proteins levels were normalized to tubulin. Images represent one replicate of two independent, biological replicates. **B**) Schematic of major metabolic pathways affected by ATF4 inhibition and their interconnections (Created in BioRender. Basse, M. (2026) https://BioRender.com/35zax27). Related metabolic enzymes that were downregulated by ATF4 inhibition are marked with red arrows. **C**) Pathway enrichment analysis of metabolites that were down-regulated by shATF4-1 using the MetaboAnalyst v5.0. **D-L**) Quantification of steady-state levels from shNT-, shATF4-1, or shATF4–2 expressing NOMO-1 cells at 48 hours post-DOX induction of: Arginine (D), Proline (E), Isoleucine (F), Leucine (G), Valine (H), Glutamate (I), Glutamine (J), Fructose-1,6-bisphosphate (K), and D-Sedoheptulose-7-phosphate (L). For panels **D-L**, all graphed data represent the mean ± SD of three technical replicates from one biological replicate. Two independent, biological replicates were carried out. Statistics are derived from a one-way ANOVA between shNT (control), shATF4-1 (pink p-values), and shATF4-2 (p-values) conditions. A Dunnett's multiple comparisons test (95% CI) was also performed. Exact adjusted p-values are listed in the Source Data File. All Source data are provided as a Source Data file.

Supplementary Figure 6

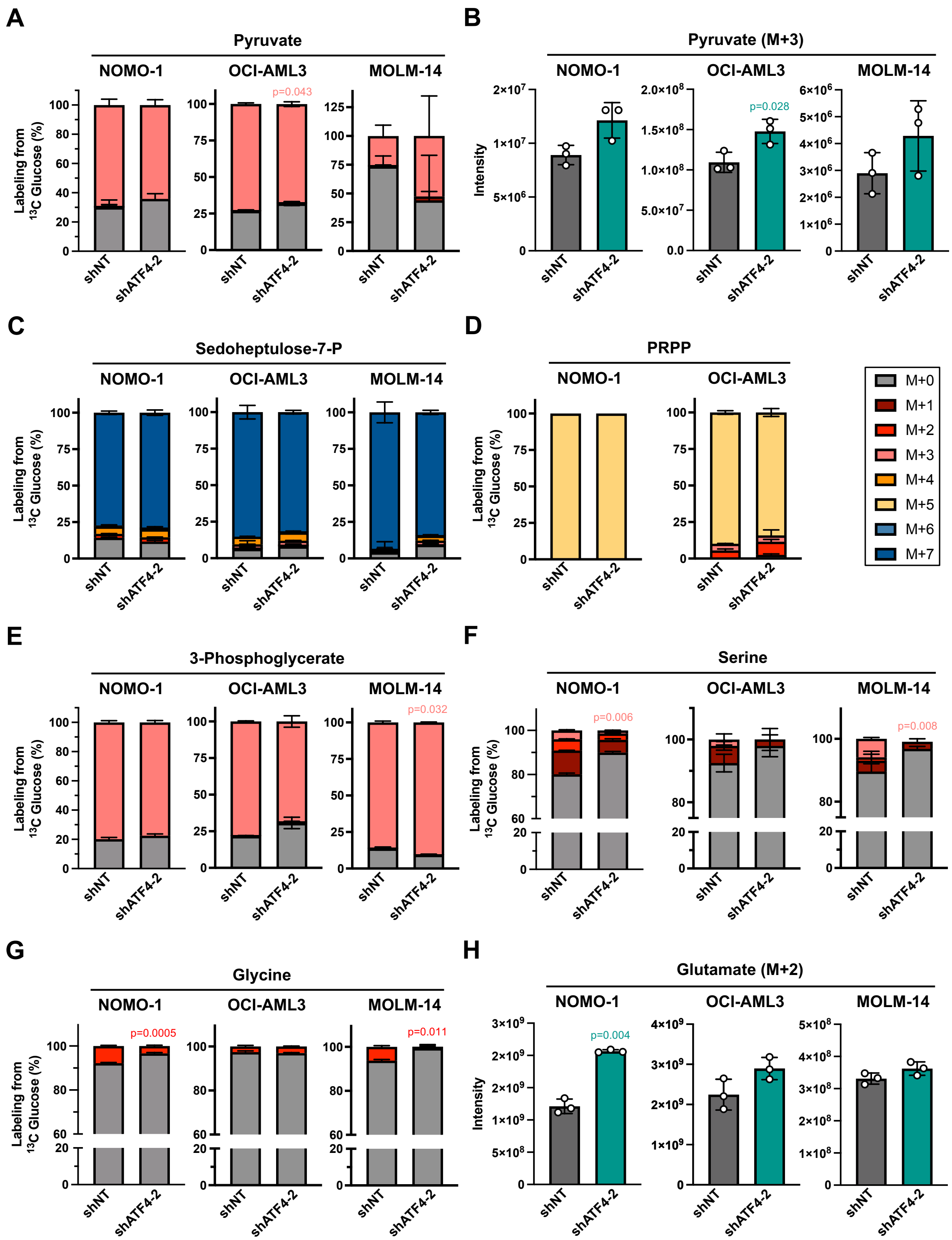

Supplementary Figure 6. DOX-treated NOMO-1, OCI-AML3 and MOLM-14 cells expressing shNT or shATF4-2 were labeled with U-^{13}C-Glucose for 16-hours and then extracted TPMs were analyzed by HILIC-MS. **A**) Percent (%) U-^{13}C-Glucose isotopologue labeling of Pyruvate. **B**) Total levels of M+3 Pyruvate labeled from U-^{13}C-Glucose. **C-G**) Percent (%) U-^{13}C-Glucose isotopologue labeling of: Sedoheptulose-7-phosphate (C), PRPP (D), 3-PG (E), Serine (F), and Glycine (G). **H**) Total levels of M+2 Glutamate labeled from U-^{13}C-Glucose. For panels, **A** and **C-G**, data represent the mean ± SD of three technical replicates. Two independent, biological replicates were performed for NOMO-1 cells and one independent, biological replicate for each OCI-AML3 and MOLM-14 cells. Statistics are derived from a two-way ANOVA (95% CI) comparing the shNT control and shATF4-2 conditions. Šídák's multiple comparisons test was applied to compare each label fraction (M+2, red and M+3, pink) between shNT (control) and shATF4-2 conditions. Exact adjusted p-values are listed in the Source Data File. For panels, **B** and **H**, data represent the mean ± SD of three technical replicates. Two independent, biological replicates were performed for NOMO-1 cells and one independent, biological replicate for each OCI-AML3 and MOLM-14 cells. Statistics are derived from a two-sided, unpaired t-test (95% CI) comparing the shNT control and shATF4-2 conditions (teal p-values). Adjustments for multiple comparisons were not made and exact p-values are listed in the Source Data File. All Source data are provided as a Source Data file.

Supplementary Figure 7

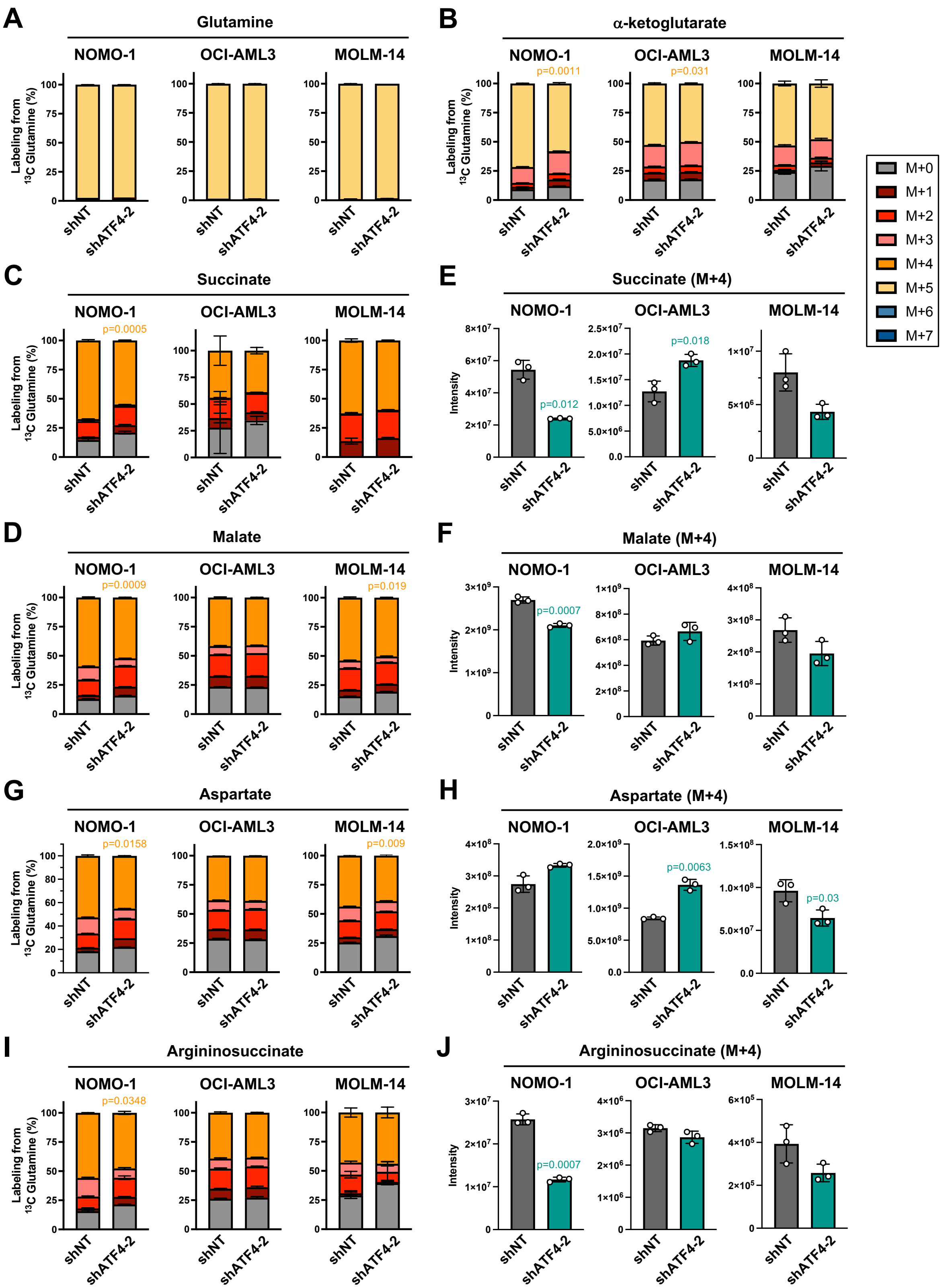

Supplementary Figure 7. NOMO-1, OCI-AML3, and MOLM-14 cells from each of the indicated shRNA conditions were labeled with U-^{13}C-Glutamine for 4 hours and extracted TPMs were then quantified by HILIC-MS. **A**) % of intracellular U-^{13}C-Glutamine. **B-D)** % U-^{13}C-Glutamine isotopologue labeling of α-KG (B), Succinate (C) and Malate (D). **E & F)** Total levels of M+4 Succinate (E) and M+4 Malate (F) labeled from U-^{13}C-Glutamine. **G)** % U-^{13}C-Glutamine isotopologue labeling of Aspartate. **H)** Total levels of M+4 Aspartate labeled from U-^{13}C-Glutamine. **I)** % U-^{13}C-Glutamine isotopologue labeling of Argininosuccinate. **J)** Total levels of M+4 Argininosuccinate labeled from U-^{13}C-Glutamine. For panels, **A-C, D, G,** and **I**, data represent the mean ± SD of three technical replicates. Two independent, biological replicates were performed for NOMO-1 cells and one independent, biological replicate for each OCI-AML3 and MOLM-14 cells. Statistics are derived from a two-way ANOVA (95% CI) comparison between shNT control and shATF4-2 conditions. Šídák's multiple comparisons test was applied to compare each label fraction between shNT and shATF4-2 conditions. P-values in panels **A** and **B** represent a comparison of the M+5 label fraction between shNT and shATF4-2 conditions. P-values in panels **D, G,** and **I** represent a comparison of the M+4 label fraction between shNT and shATF4-2 conditions. Exact adjusted p-values are listed in the Source Data File. For panels, **E, F, H** and **J**, data represent the mean ± SD of three technical replicates. Two independent, biological replicates were performed for NOMO-1 cells and one independent, biological replicate for each OCI-AML3 and MOLM-14 cells. Statistics are derived from a two-sided, unpaired t-test (95% CI) comparing the shNT control and shATF4-2 conditions (teal p-values). Adjustments for multiple comparisons were not made and exact p-values are listed in the Source Data File. All Source data are provided as a Source Data file.

Supplementary Figure 8

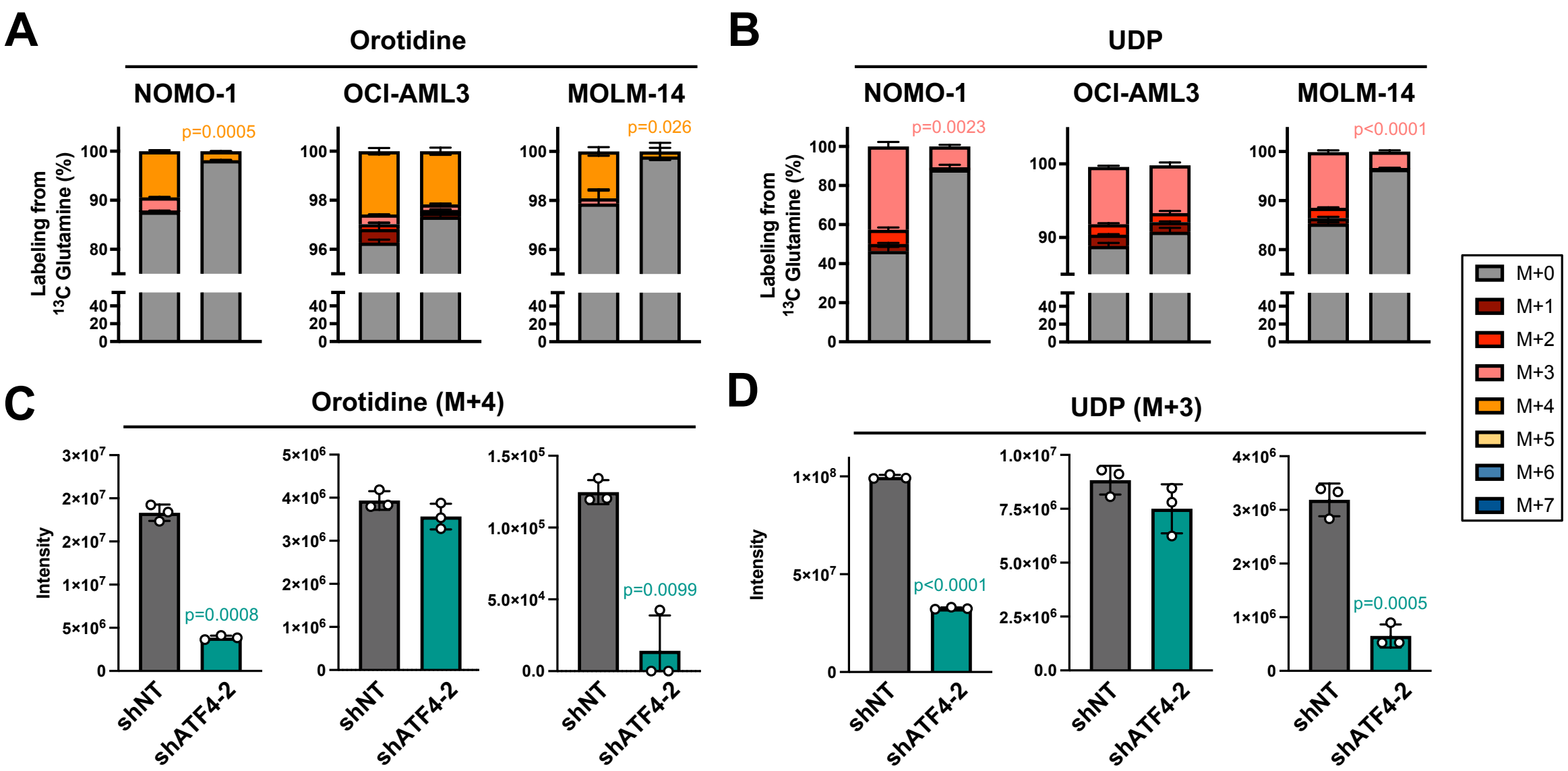

E

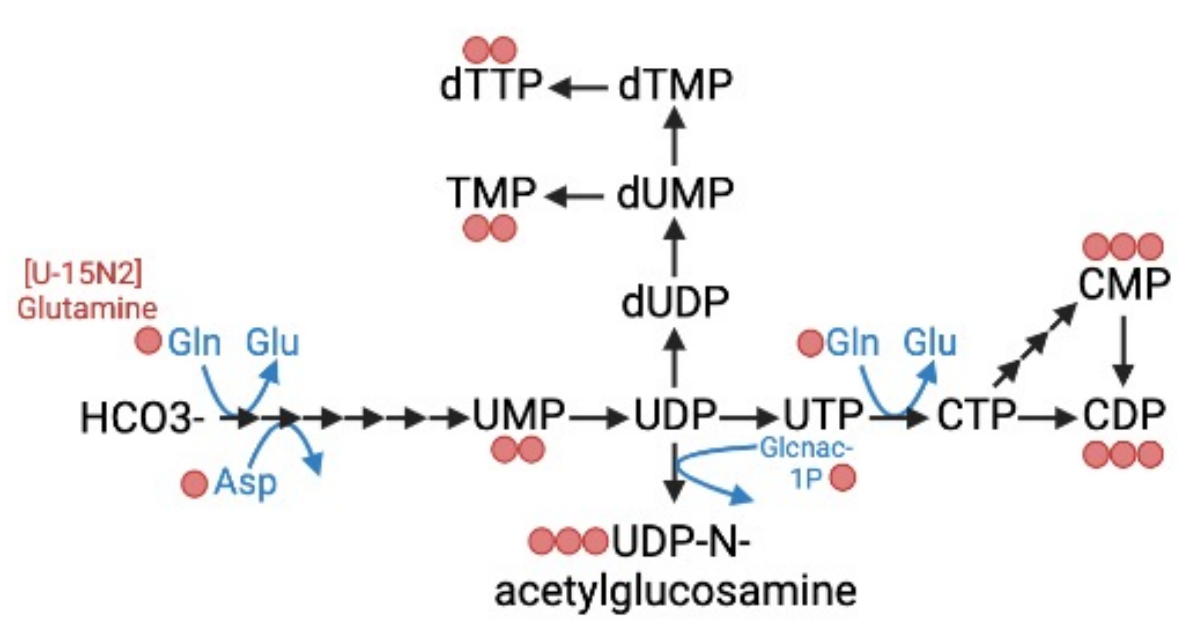

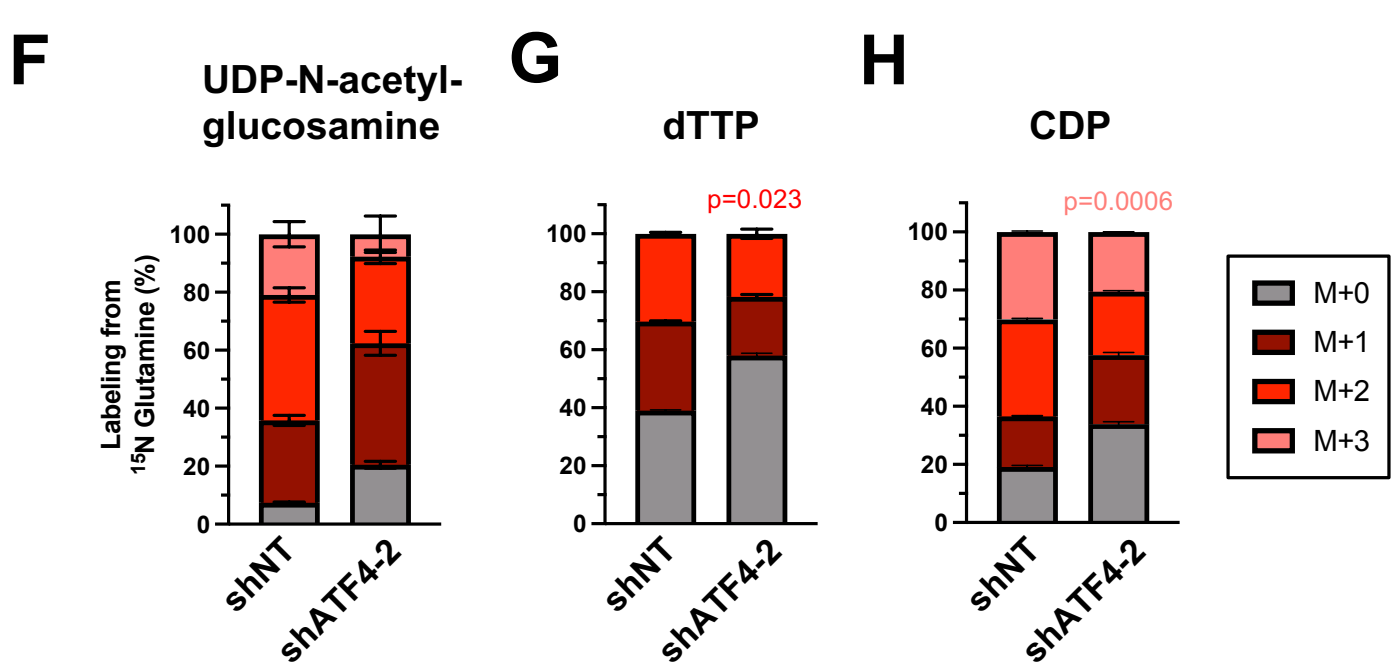

I

Gene Name	Fold Change	FDR
PPAT	-0.315	1.43 x 10^{-4}
GART	-0.775	1.88 x 10^{-74}
PFAS	-0.431	4.83 x 10^{-10}
PAICS	-0.379	1.16 x 10^{-30}
ADSL	-0.281	0.561
ATIC	-0.509	8.02 x 10^{-17}
ADSSL1	0.613	9.59 x 10^{-4}
ADSS	-0.157	7.63 x 10^{-3}
ADSL	-0.281	0.561
AK1	0.360	1
AK2	-0.429	7.33 x 10^{-33}
AK3	0.157	0.0259
RRM1	-0.361	8.61 x 10^{-17}
RRM2	-0.400	1.24 x 10^{-11}
NME1	-0.151	0.198
NME2	-0.457	0.505
NME3	0.135	0.849
NME4	0.003	0.985
NME6	0.281	0.00882
NME7	0.217	0.218
IMPDH1	0.123	0.0777
IMPDH2	-0.829	4.40 x 10^{-131}
GMPS	-0.189	6.25 x 10^{-5}
GUK1	0.002	0.989
CAD	-0.181	2.14 x 10^{-5}
DHODH	-0.419	0.00314
UMPS	-0.218	0.0422
CTPS1	0.024	0.807
CTPS2	0.164	0.415

Supplementary Figure 8. NOMO-1, OCI-AML3, and MOLM-14 cells from each of the indicated shRNA conditions were labeled with U-^{13}C-Glutamine for 4 hours and extracted TPMs were then quantified by HILIC-MS. **A-B)** % U-^{13}C-Glutamine isotopologue labeling of Orotidine (A) and UDP (B). **C & D)** Total levels of M+3 Orotidine (C) and M+3 UDP (D). For panels, **A** and **B**, data represent the mean ± SD of three technical replicates. Two independent, biological replicates were performed for NOMO-1 cells and one independent, biological replicate for each OCI-AML3 and MOLM-14 cells. Statistics are derived from a two-way ANOVA (95% CI) comparison between shNT (control) and shATF4-2 conditions. Šídák's multiple comparisons test was applied to compare each label fraction between shNT and shATF4-2 conditions. P-values in panel **A** represent a comparison of the M+4 label fraction between shNT and shATF4-2 conditions. P-values in panel **B** represent a comparison of the M+3 label fraction between shNT and shATF4-2 conditions. Exact adjusted p-values are listed in the Source Data File. For panels, **C** and **D**, data represent the mean ± SD of three technical replicates. Two independent, biological replicates were performed for NOMO-1 cells and one independent, biological replicate for each OCI-AML3 and MOLM-14 cells. Statistics are derived from a two-sided, unpaired t-test (95% CI) between shNT (control) and shATF4-2 (teal p-values) conditions. Adjustments for multiple comparisons were not made and exact p-values are listed in the Source Data File. **E)** Schematic of U-^{15}N-Glutamine utilization in pyrimidine synthesis. Red circles represent ^{15}N atoms derived from U-^{15}N-Glutamine (Created in BioRender. Basse, M. (2026) https://BioRender.com/reqi3kf). **F-H**) % U-^{15}N-Glutamine isotopologue labeling of UDP-N-acetylglucosamine (F), dTTP (G), and CDP (H). For panels, **F-H**, data represent the mean ± SD of three technical replicates of the one independent, biological replicate that was performed. Statistics are derived from a two-way ANOVA (95% CI) comparison between shNT (control) and shATF4-2 conditions. Šídák's multiple comparisons test

was applied to compare each label fraction between shNT and shATF4-2 conditions. P-values in panels **G** represent a comparison of the M+2 label fraction between shNT and shATF4-2 conditions. P-values in panels **H** represent a comparison of the M+3 label fraction between shNT and shATF4-2 conditions. **I**) Differential expression fold change of denoted purine and pyrimidine synthesis genes from ATF4-shRNA-expressing NOMO-1 cells compared with shNT controls. All Source data are provided as a Source Data file.

Supplementary Figure 9

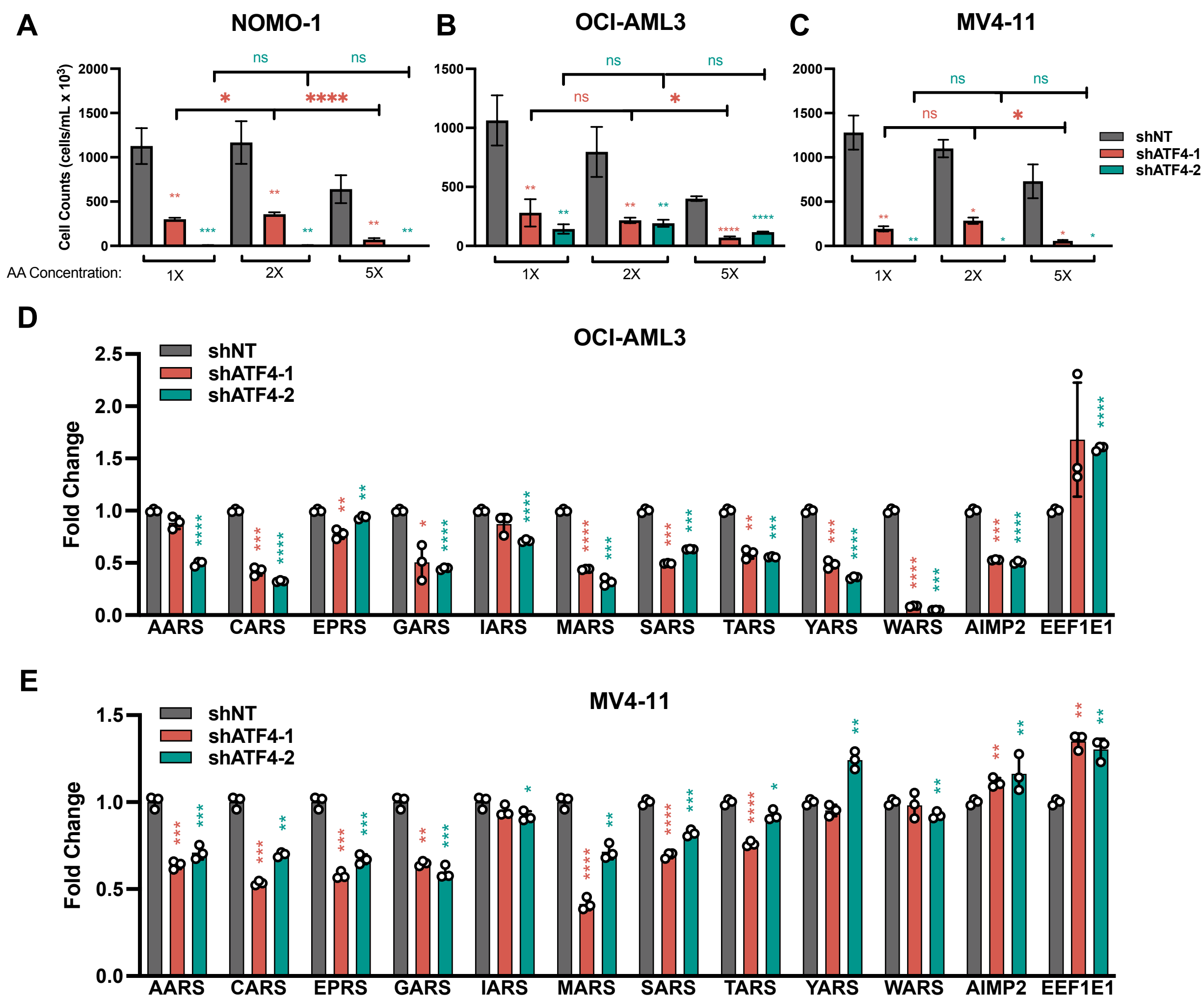

F

Gene Name	Corresponding Amino Acid(s)
AARS	Alanine
CARS	Cysteine
EPRS	Glutamate, Proline
GARS	Glycine
IARS	Isoleucine
LARS	Leucine
MARS	Methionine
SARS	Serine
TARS	Threonine
YARS	Tyrosine
WARS	Tryptophan

Supplementary Figure 9. A-C) NOMO-1 (A), OCI-AML3 (B), and MV4-11 (C) from each shRNA condition were treated with DOX and cultured in RPMI-1640-based media supplemented with standard concentrations (1x), double the concentration (2X), or five times the standard concentration (5X) of affected amino acids (arginine, asparagine, aspartic acid, cysteine, glutamic acid, glutamine glycine, proline, and serine). Cells from each condition were then assessed for cell viability by dye exclusion test with trypan blue at 7 days post-DOX treatment. Data represent the mean ± SD of three technical replicates from one biological replicate. Two independent, biological replicate was carried out for each cell line. Statistics are derived from a two-sided, unpaired t-test comparison between shNT (control) and shATF4-1 or shNT and shATF4-2 conditions, $*P \leq 0.05$; $**P \leq 0.01$; $***P \leq 0.001$. **D & E**) qPCR analysis of indicated tRNA aminoacylation mRNAs in OCI-AML3 (D) and MV411 (E) expressing the indicated shRNAs. Data represent the mean ± SD of three technical replicates from one biological replicate. Two independent, biological replicate was carried out for each cell line. Statistics are derived from a two-sided, unpaired t-test comparing the shNT control condition with either the shATF4-1 or shATF4-2 conditions. Adjustments for multiple comparisons were not made. Asterisk key: $*P \leq 0.05$; $**P \leq 0.01$; $***P \leq 0.001$; $****P \leq 0.0001$. **F**) List of tRNA aminoacyl synthetases that were downregulated in RNA-seq data from shATF4-2 cells compared to shNT controls. Amino acids that were also decreased in Fig. 3 metabolomics are highlighted in red. All Source data are provided as a Source Data file.

Supplementary Figure 10

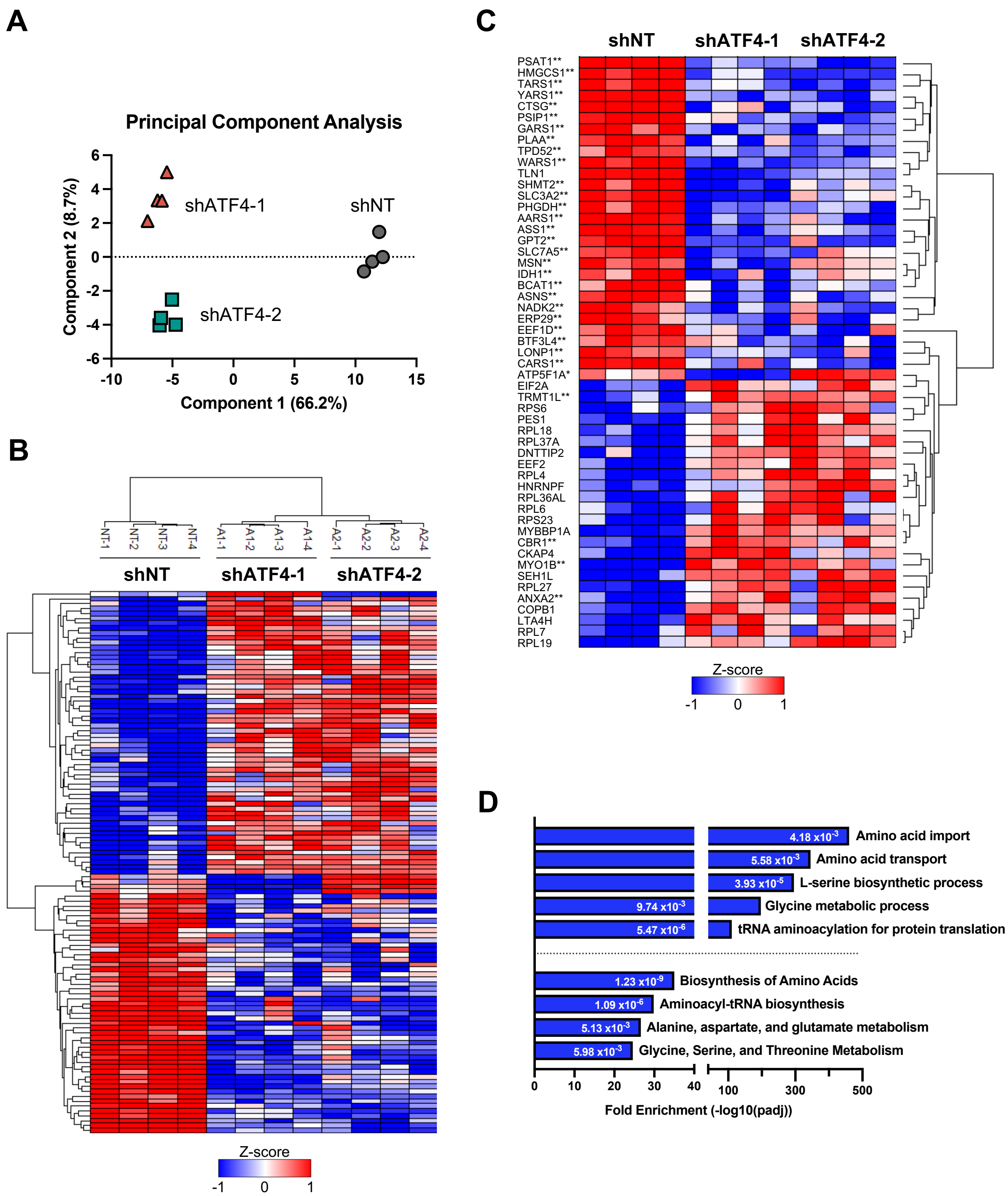

Supplementary Figure 10. A) PCA of significantly differentially abundant proteins in shNT, shATF4-1, and shATF4-2 expressing NOMO-1 cells utilizing 66.2% of data in component 1 and 8.7% of data in component 2. **B**) Heatmap of proteins that were significantly differentially abundant ($p < 0.05$) in ATF4-shRNA-expressing NOMO-1 cells compared with shNT controls. **C**) Heatmap of proteins that were significantly differentially abundant ($p < 0.05$) in ATF4-shRNA-expressing NOMO-1 cells compared with shNT controls filtered for genes that were also significantly differentially expressed ($p <0.05$) in the RNAseq dataset of ATF4-shRNA-expressing NOMO-1 cells compared with shNT controls. Asterisks indicate genes in which the protein abundance fold change and the RNA expression fold change are in the same direction in one (*) or both (**) shATF4 conditions. **D**) Pathway enrichment analysis of significantly decreased proteins filtered for RNA significance in ATF4-shRNA-expressing NOMO-1 cells compared with shNT controls using the DAVID bioinformatic resource version 6.8. The analysis was performed using GO_BP (top) and KEGG (bottom). Benjamini scores are shown in white within the bar graph. All Source data are provided as a Source Data file.

Supplementary Figure 11

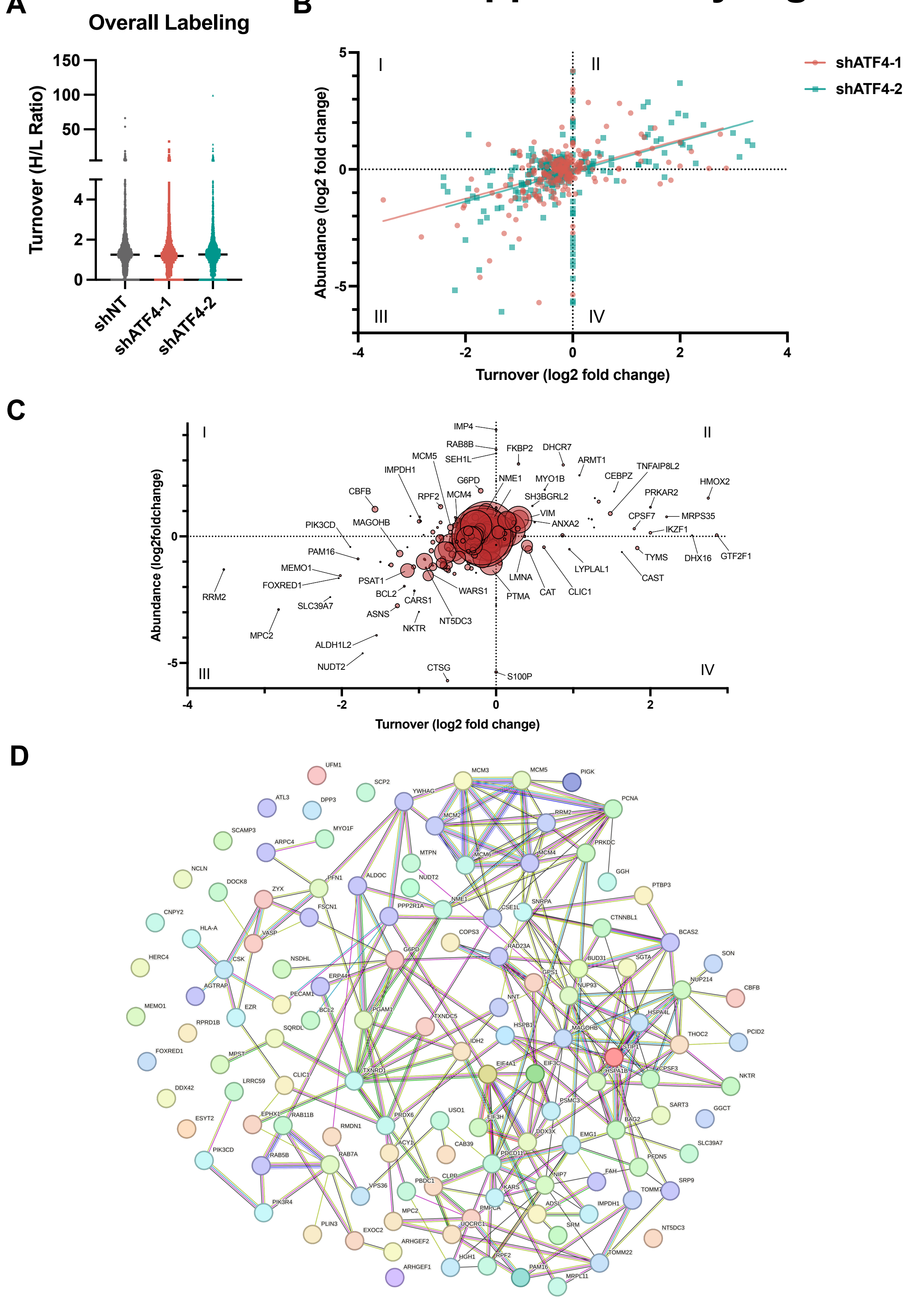

Supplementary Figure 11. A) Turnover ratio of heavy isotope (H) labeled to non-labeled (L) of all detected proteins from NOMO-1 cells of each indicated shRNA condition. **B**) Protein turnover and abundance correlation of significantly differentially labeled proteins in ATF4-shRNA-expressing NOMO-1 cells compared with shNT controls. **C**) Protein turnover and abundance correlation of significantly differentially labeled proteins in ATF4-1-shRNA-expressing NOMO-1 cells compared with shNT controls. Average abundance of each protein is represented by the size of the corresponding circle. **D**) StringDB v11.5-based clustering analysis of proteins with a significant decrease in protein turnover (but not a significant difference in RNA expression) in shATF4-expressing NOMO-1 cells compared to controls. All Source data are provided as a Source Data file.

Supplementary Figure 12

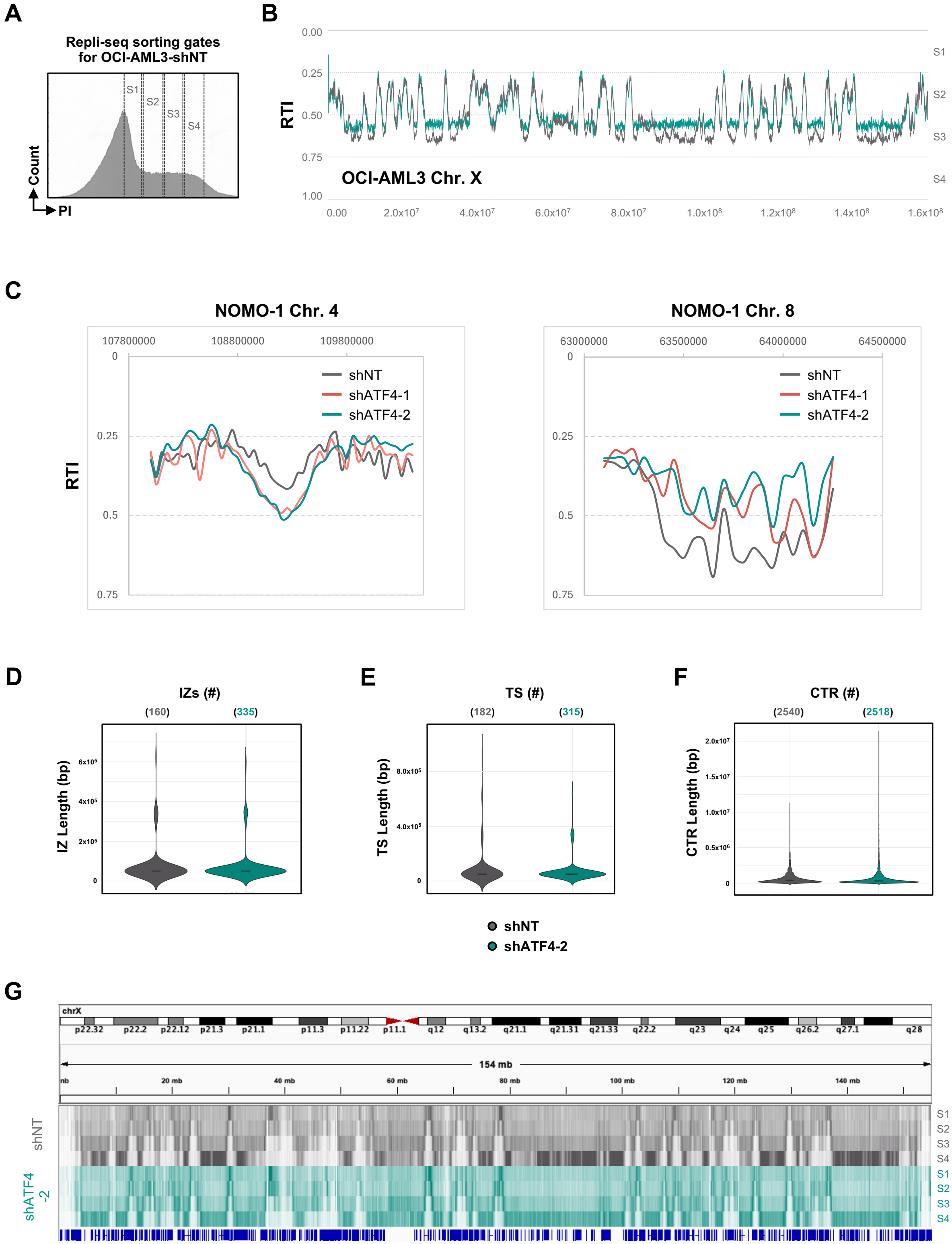

Supplementary Figure 12. **A**) Flow cytometric plot of OCI-AML3-shNT cells including gates used to sort different S-phases (S1-S4) based on PI staining. **B**) Quantitative tracks of RTI of the X chromosome from OCI-AML3 cells expressing shNT versus those expressing shATF4-2. **C**) Quantitative RTI tracks of the indicated regions of chromosome 4 (*left panel*) and chromosome 8 (*right panel*) from NOMO-1 expressing shNT, shATF4-1, or shATF4-2. **D**) Lengths of IZs calculated from Repli-seq signals from OCI-AML3-shNT and -shATF4-2 cells. **E**) TSs calculated from Repli-seq signals from OCI-AML3-shNT and -shATF4-2 cells. **F**) CTRs calculated from Repli-seq signals from OCI-AML3-shNT and -shATF4-2 cells. **G**) Representative genomic tracks of the X chromosome with S1-S4 repli-seq signals from OCI-AML3-shNT and -shATF4-2 cells. All Source data are provided as a Source Data file.

Supplementary Figure 13

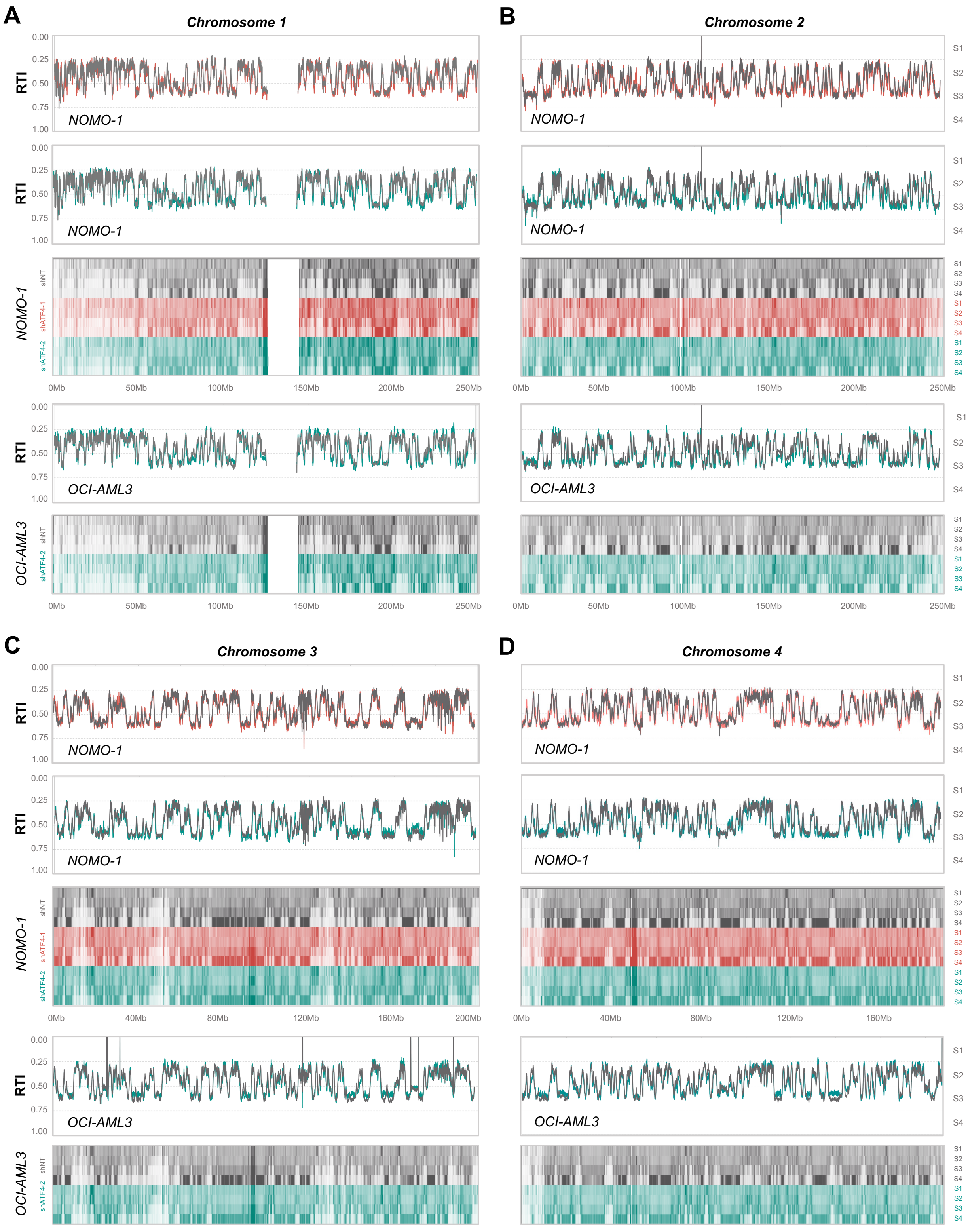

Supplementary Figure 13. A-D) For chromosome 1 (A), chromosome 2 (B), chromosome 3 (C), and chromosome 4 (D): Quantitative tracks of RTI across the indicated chromosomes for NOMO-1-shNT versus -shATF4-1 (*top panel*), NOMO-1-shNT versus -shATF4-2 (*second panel from the top*), and OCI-AML3-shNT versus -shATF4-2 (*second panel from the bottom*) as well as representative genomic tracks of the indicated chromosomes with S1-S4 repli-seq signals from NOMO-1-shNT, -shATF4-1, and -shATF4-2 cells (*middle panel*) and OCI-AML3-shNT and -shATF4-2 cells (*bottom panel*). All Source data are provided as a Source Data file.

Supplementary Figure 14

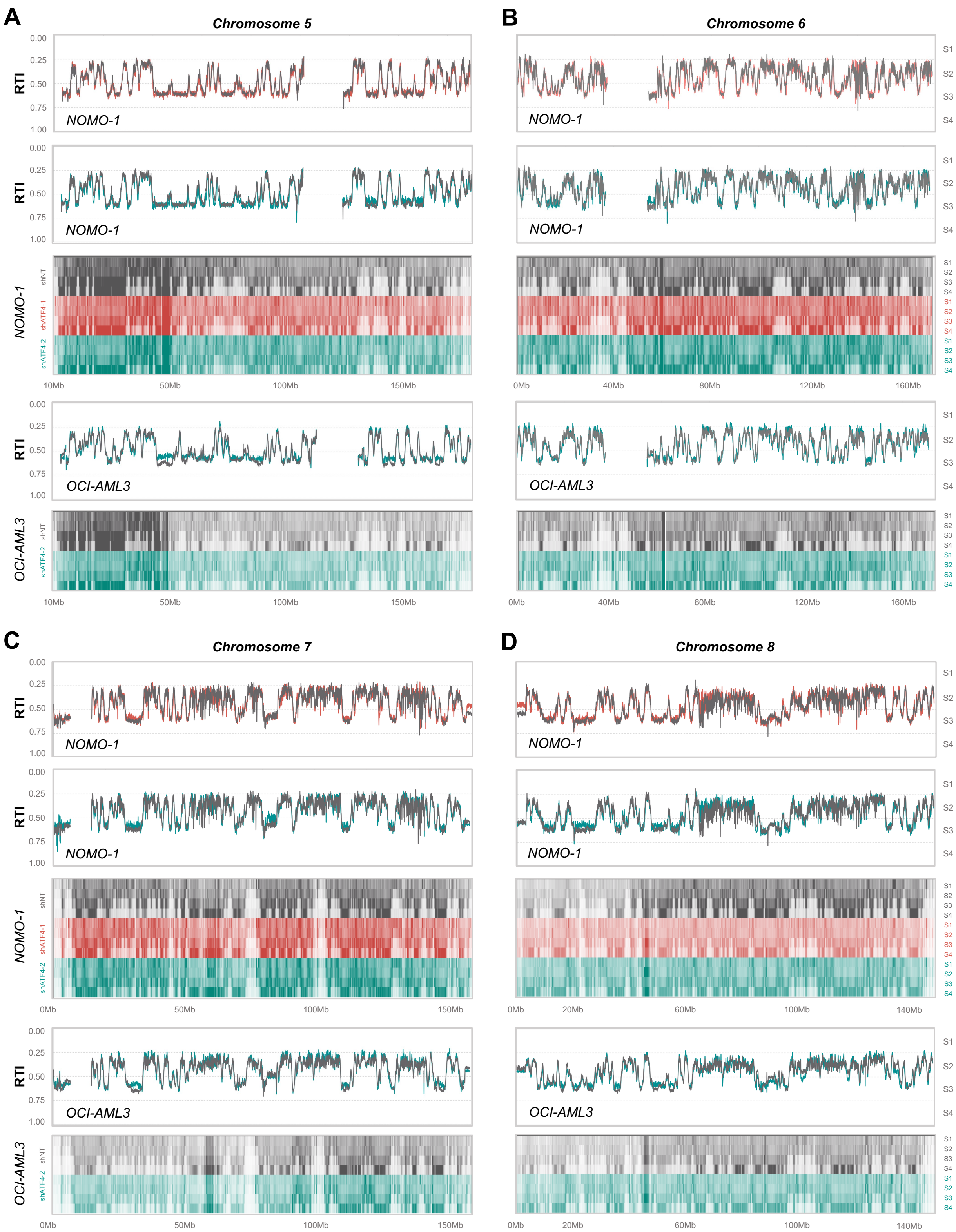

Supplementary Figure 14. A-D) For chromosome 5 (A), chromosome 6 (B), chromosome 7 (C), and chromosome 8 (D): Quantitative tracks of RTI across the indicated chromosomes for NOMO-1-shNT versus -shATF4-1 (*top panel*), NOMO-1-shNT versus -shATF4-2 (*second panel from the top*), and OCI-AML3-shNT versus -shATF4-2 (*second panel from the bottom*) as well as representative genomic tracks of the indicated chromosomes with S1-S4 repli-seq signals from NOMO-1-shNT, -shATF4-1, and -shATF4-2 cells (*middle panel*) and OCI-AML3-shNT and -shATF4-2 cells (*bottom panel*). All Source data are provided as a Source Data file.

Supplementary Figure 15

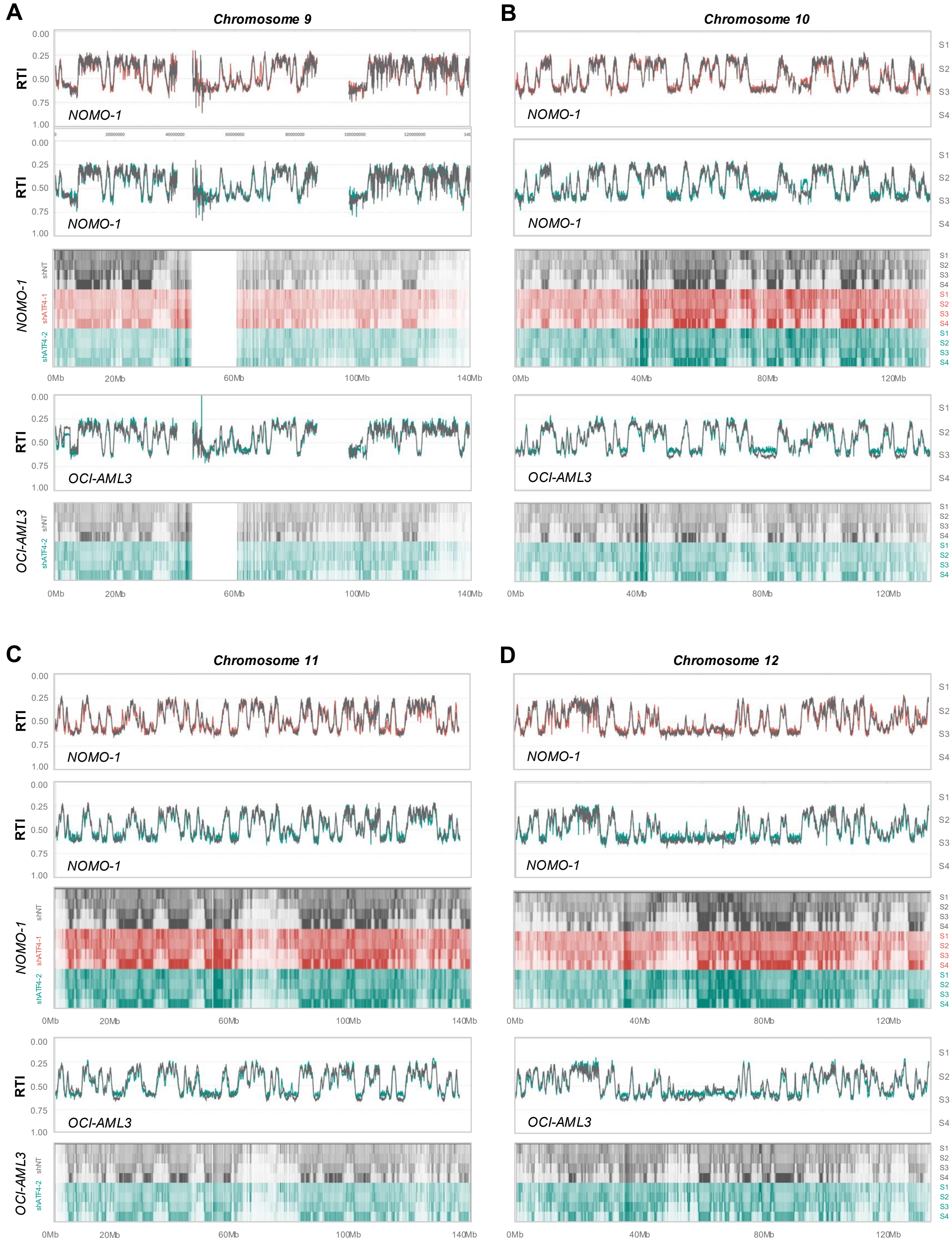

Supplementary Figure 15. A-D) For chromosome 9 (A), chromosome 10 (B), chromosome 11 (C), and chromosome 12 (D): Quantitative tracks of RTI across the indicated chromosomes for NOMO-1-shNT versus -shATF4-1 (*top panel*), NOMO-1-shNT versus -shATF4-2 (*second panel from the top*), and OCI-AML3-shNT versus -shATF4-2 (*second panel from the bottom*) as well as representative genomic tracks of the indicated chromosomes with S1-S4 repli-seq signals from NOMO-1-shNT, -shATF4-1, and -shATF4-2 cells (*middle panel*) and OCI-AML3-shNT and -shATF4-2 cells (*bottom panel*). All Source data are provided as a Source Data file.

Supplementary Figure 16

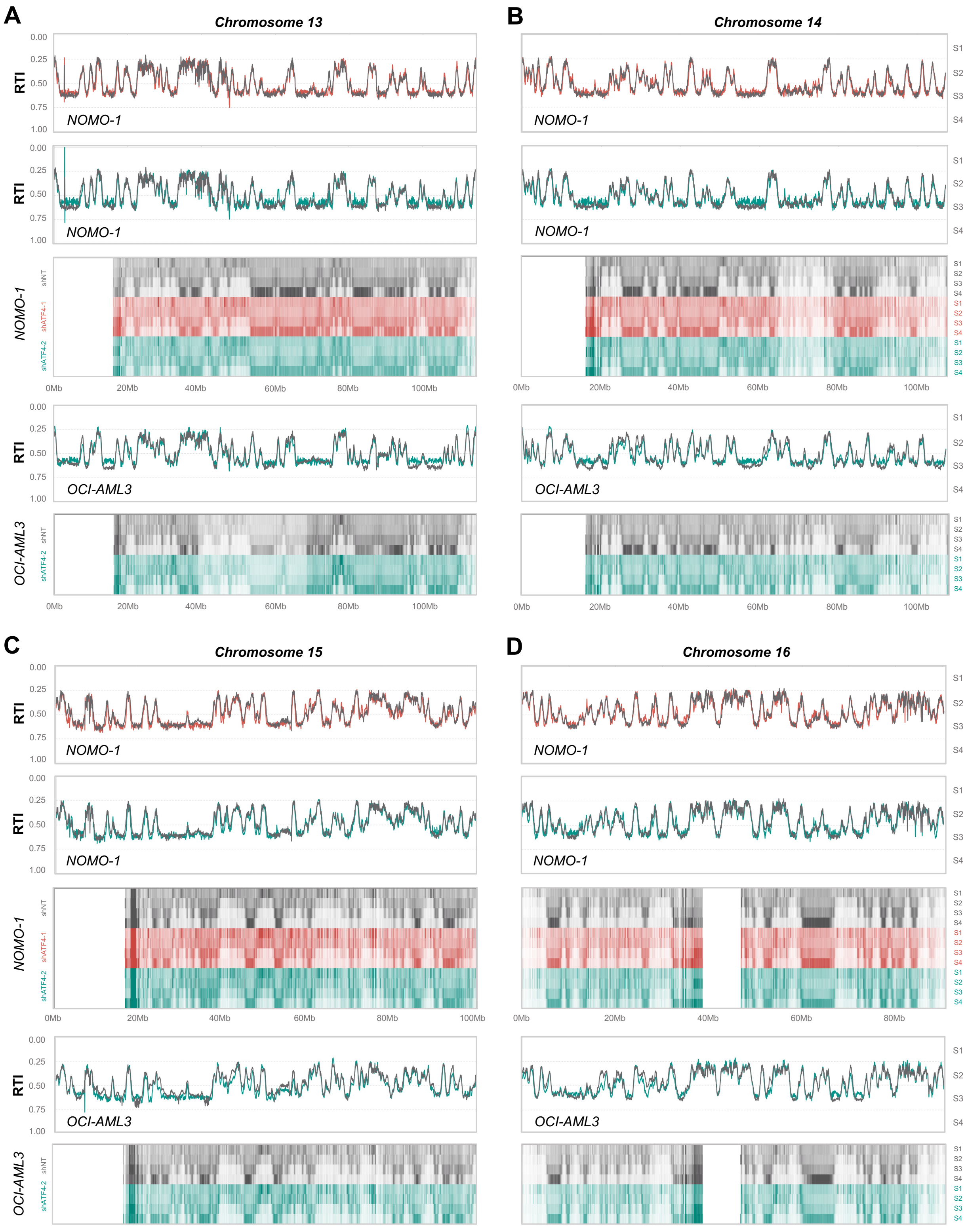

Supplementary Figure 16. A-D) For chromosome 13 (A), chromosome 14 (B), chromosome 15 (C), and chromosome 16 (D): Quantitative tracks of RTI across the indicated chromosomes for NOMO-1-shNT versus -shATF4-1 (*top panel*), NOMO-1-shNT versus -shATF4-2 (*second panel from the top*), and OCI-AML3-shNT versus -shATF4-2 (*second panel from the bottom*) as well as representative genomic tracks of the indicated chromosomes with S1-S4 repli-seq signals from NOMO-1-shNT, -shATF4-1, and -shATF4-2 cells (*middle panel*) and OCI-AML3-shNT and -shATF4-2 cells (*bottom panel*). All Source data are provided as a Source Data file.

Supplementary Figure 17

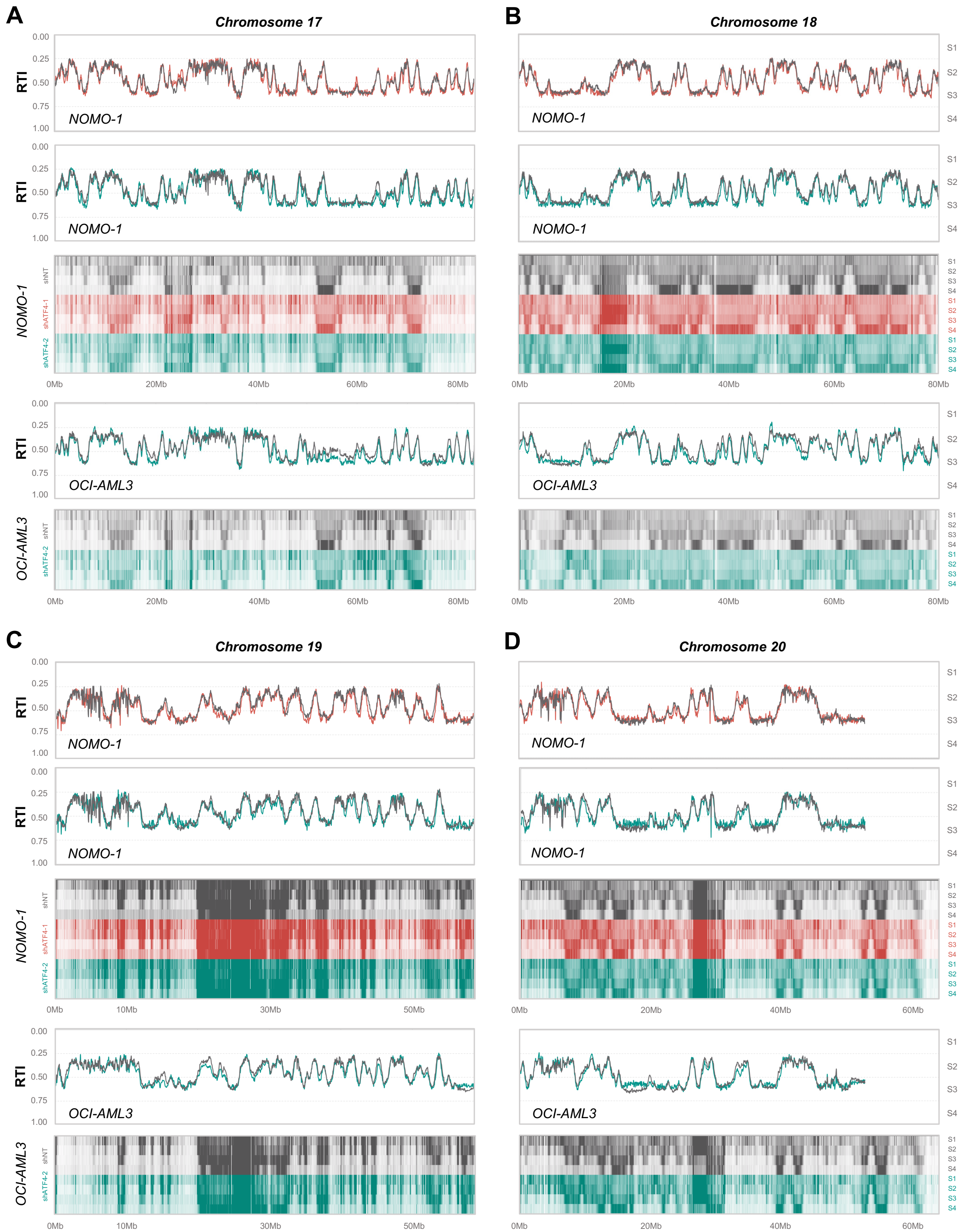

Supplementary Figure 17. A-D) For chromosome 17 (A), chromosome 18 (B), chromosome 19 (C), and chromosome 20 (D): Quantitative tracks of RTI across the indicated chromosomes for NOMO-1-shNT versus -shATF4-1 (*top panel*), NOMO-1-shNT versus -shATF4-2 (*second panel from the top*), and OCI-AML3-shNT versus -shATF4-2 (*second panel from the bottom*) as well as representative genomic tracks of the indicated chromosomes with S1-S4 repli-seq signals from NOMO-1-shNT, -shATF4-1, and -shATF4-2 cells (*middle panel*) and OCI-AML3-shNT and -shATF4-2 cells (*bottom panel*). All Source data are provided as a Source Data file.

Supplementary Figure 18

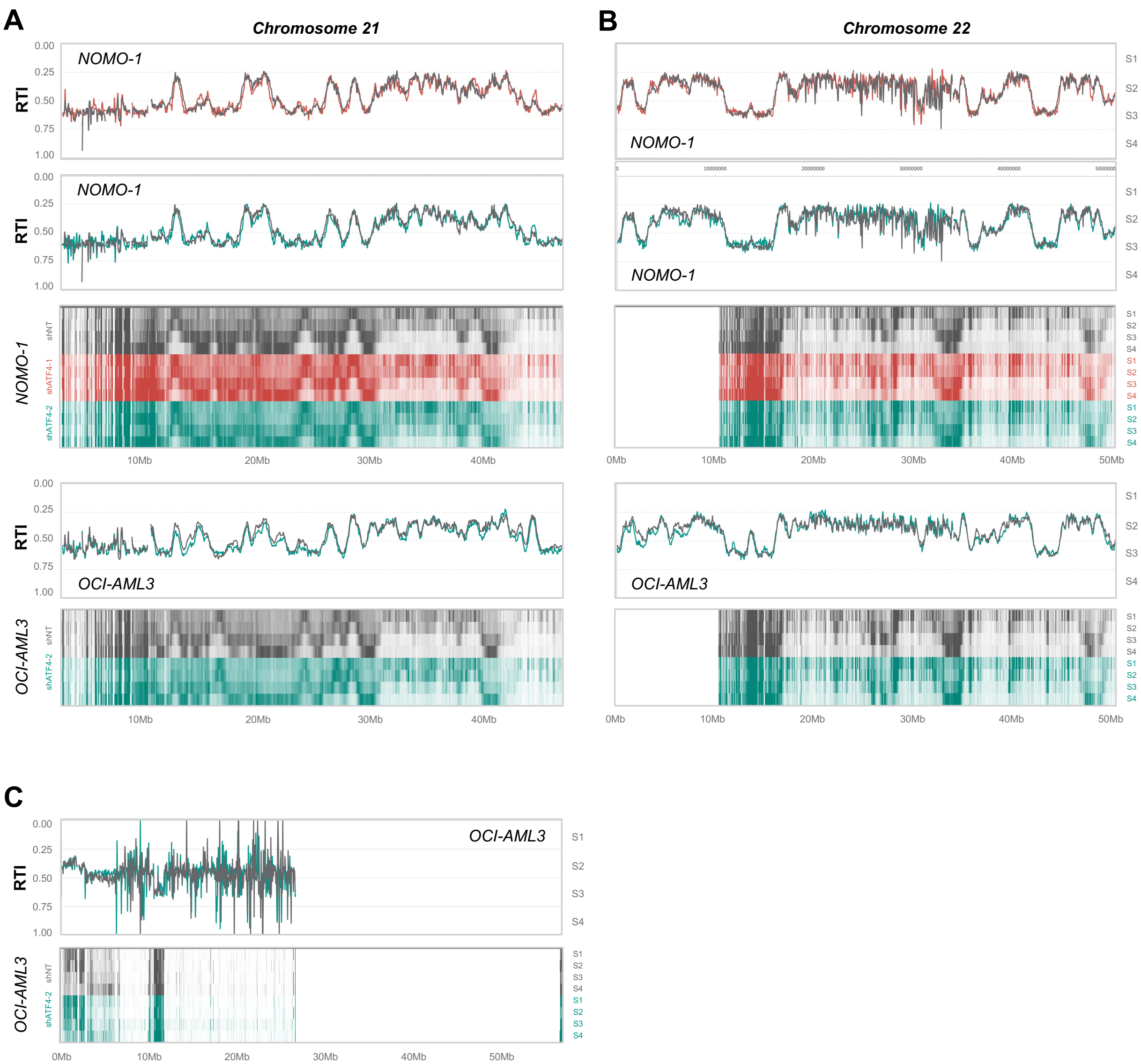

Supplementary Figure 18. A & B) For chromosome 21 (A) and chromosome 22 (B): Quantitative tracks of RTI across the indicated chromosomes for NOMO-1-shNT versus -shATF4-1 (*top panel*), NOMO-1-shNT versus -shATF4-2 (*second panel from the top*), and OCI-AML3-shNT versus -shATF4-2 (*second panel from the bottom*) as well as representative genomic tracks of the indicated chromosomes with S1-S4 repli-seq signals from NOMO-1-shNT, -shATF4-1, and -shATF4-2 cells (*middle panel*) and OCI-AML3-shNT and -shATF4-2 cells (*bottom panel*). **C**) Quantitative tracks of RTI across the Y chromosome for OCI-AML3-shNT versus -shATF4-2 (*top panel*) as well as representative genomic tracks of the Y chromosome with S1-S4 repli-seq signals from OCI-AML3-shNT and -shATF4-2 cells (*bottom panel*). All Source data are provided as a Source Data file.
