# Supplementary material for: ATF4 coordinates amino acid and nucleotide synthesis with selective protein translation to ensure proper DNA replication timing in leukemia cells
Source: Nat Commun. 2026 Jun 20;17:7760. doi: 10.1038/s41467-026-74324-1 (PMC13434596; doi:10.1038/s41467-026-74324-1)
Supplement: Supplementary file 4 [file 41467_2026_74324_MOESM2_ESM.pdf]

Description of Additional Supplementary Files

File name: Supplementary Data 1
Description: Description of the Patient-Derived AML sample population characteristics including genetic subtypes and covariate-relevant population characteristics.

File name: Supplementary Data 2
Description: Functional clustering analyses results of differentially expressed genes (DEGs) mediated by ATF4 inhibition using Database for Annotation, Visualization and Integrated Discovery (DAVID).

File name: Supplementary Data 3
Description: Comparison of differentially expressed genes (DEGs) between ATF4 inhibition in NOMO-1 cells and KMT2A-driven transcriptional programs.

File name: Supplementary Data 4
Description: Results of gene set enrichment analysis of ATF4 transcriptional targets using the Molecular Signatures Database (MSigDb).

File name: Supplementary Data 5
Description: Functional clustering analysis of proteins that are differentially translated upon ATF4 inhibition using Database for Annotation, Visualization and Integrated Discovery (DAVID).

File name: Supplementary Data 6
Description: List of oligonucleotides for qPCR analyses throughout the study.

File name: Supplementary Data 7
Description: Information of antibodies used throughout the study including vendors, catalogue numbers, clone numbers, and dilution factors.
